# Surface temperatures drive strong seasonality in urban reactive carbon emissions

**DOI:** 10.1126/sciadv.aef9622

**Published:** 2026-10-07

**Authors:** Michael P. Vermeuel, Dylan B. Millet, Róisín Commane, Timothy J. Griffis, Trey A. Maddaleno, Emily B. Franklin, Katelyn L. Richard, Rose K. Rossell, Jeff Peischl, Delphine K. Farmer

**Affiliations:** ^1^ Department of Chemistry, Colorado State University, Fort Collins, CO, USA.; ^2^ Department of Soil, Water, and Climate, University of Minnesota, Twin Cities, St. Paul, MN, USA.; ^3^ Department of Earth, Atmospheric, and Planetary Sciences, Purdue University, West Lafayette, IN, USA.; ^4^ Department of Earth and Environmental Sciences, Columbia University, Palisades, NY, USA.; ^5^ Environment, Commonwealth Scientific and Industrial Research Organization, Aspendale, VIC, Australia.; ^6^ Global Monitoring Laboratory, National Oceanic and Atmospheric Administration, Boulder, CO, USA.; ^7^ Cooperative Institute for Research in Environmental Sciences, University of Colorado Boulder, Boulder, CO, USA.

## Abstract

Urban air quality is affected by diverse volatile organic compounds (VOCs), and prior studies reveal a need to understand their seasonal sources. We combine high-resolution mass spectrometry with eddy covariance to quantify VOC fluxes from an urban/suburban New York site during summer and winter. Emissions are strongly seasonal: During summer, twice as many VOCs undergo surface-atmosphere exchange, and molar and reactivity-based fluxes are 2 to 4× higher than in winter. Temperature-dependent emissions from volatile chemical products (VCPs), vegetation, and residences dominate during summer. Ethanol alone accounts for ∼25% of the total flux. During winter, temperature-dependent emissions are reduced and traffic sources dominate. An updated inventory agrees with summer observations within 40% but overestimates winter fluxes by >2.5×. The winter discrepancy reflects overestimated VCP/cooking emissions and missing temperature-dependent volatilization. Results highlight the need to account for seasonal and temperature-dependent urban emissions to support pollution and mitigation assessment in the context of global change.

## INTRODUCTION

Volatile organic compounds (VOCs) control the atmosphere’s oxidative capacity via their interactions with HO*_x_* (OH + HO_2_) and NO*_x_* (NO + NO_2_), and are precursors of ozone (O_3_) and secondary organic aerosol (SOA). This chemical cascade affects Earth’s climate through a range of direct and indirect pathways (*1*). VOCs also have both direct (*2*, *3*) and indirect impacts on human health, the latter through the cardiovascular and respiratory toxicity of O_3_ and SOA (*4*, *5*). Globally, VOCs have both biogenic and anthropogenic sources, whereas urban emissions have historically been dominated by fossil fuel combustion—in particular from mobile sources (*6*, *7*). However, successful emission regulations and technological improvements have reduced fossil fuel emissions (*8*, *9*), potentially shifting the balance of urban VOC sources to nontraditional, nonfossil sectors (*10*). The relative importance of these emerging VOC sources has not yet been rigorously evaluated against flux observations in the modern American megacity.

Concentration-based measurements and model analyses have identified several nonfossil VOC sources as potentially major contributors to the urban reactive carbon budget. Volatile chemical products (VCPs; such as personal care products, pesticides, adhesives, cleaning agents, and architectural coatings) have been estimated to make up 40 to 80% of VOC emissions in US and European cities (*11*, *12*), and scale with population density (*13*, *14*). Natural emissions from urban vegetation have been found to drive O_3_ levels on hot days, even in dense cities like New York (*15*). Commercial and residential cooking also emits VOCs and was estimated to account for 20% of the anthropogenic source in Las Vegas, with a large contribution from ethanol and >C7 aldehydes (*16*). Building materials, including lumber, plywood, and fiberboard, are potential sources of monoterpenes and oxygenated compounds (e.g., <C7 aldehydes, methanol, acetone, and acetic acid) in indoor environments and near industrial sources (*17*, *18*) but have not historically been considered as drivers of urban VOCs. Decomposition of those building materials is accelerated by the high temperatures in residential attics, with additional oxygenated emissions (e.g., furfural) (*19*) that can exfiltrate outdoors and contribute to urban chemistry. More broadly, there is a growing consensus that indoor sources are relevant to outdoor air quality (*19*, *20*), but evidence for these effects has generally been limited to order-of-magnitude estimates based on indoor concentrations and air change rates.

These nontraditional VOC sources are poorly constrained by observations and inadequately understood in terms of drivers. One clear challenge for understanding and modeling urban reactive carbon is its temperature dependence. Surface temperature is a well-studied and clearly parameterized driver of biogenic emissions (*21*–*26*), but its influence is rarely considered for anthropogenic VOCs, despite their often semivolatile nature and the well-established temperature dependence for many urban processes, including evaporation [e.g., VCPs (*27*–*29*) and gasoline (*30*–*32*)], surface degradation [e.g., asphalt (*33*) and building materials], building ventilation rates, and combustion-related energy use for indoor climate control. Characterizing and accounting for the temperature dependence of these emissions is crucial for robust air quality predictions and planning (*28*, *34*).

Bottom-up inventory-based estimates of urban VOC emissions extrapolate incomplete information on the above processes across highly heterogeneous city landscapes, which leads to inherent uncertainties (*35*, *36*). Meanwhile, top-down assessments commonly rely on species:species concentration correlations, either directly (*37*, *38*) or via positive matrix factorization (PMF), a statistical model that interprets those correlations in terms of underlying emission sector profiles. Such approaches have provided critical community tools for evaluating and improving inventories (*39*). However, in relying on ambient concentration data they suffer from their own uncertainties due to the confounding factors that affect emission:concentration relationships, including meteorology (*40*), chemical production and loss (*41*), and variable regional backgrounds.

By contrast, eddy covariance (EC) enables direct measurements of surface-atmosphere exchange to quantify VOC emission sources (*35*, *36*, *42*–*44*). Such measurements are challenging in urban areas, and only a few datasets exist to compare with urban VOC inventories. Those few studies have largely focused on summer, limiting the ambient temperature range. The less-studied winter conditions offer a contrasting minimum in both temperature and biogenic activity that can inform source attribution. For example, one multiseason urban flux study in Innsbruck, Austria showed that terpene emissions varied by over an order of magnitude between early spring and summer, driven by a shift from mainly anthropogenic to mainly biogenic sources (*45*).

Here, we combine high-resolution proton-transfer mass spectrometry (PTRMS) with the EC technique to directly quantify VOC emissions over a densely populated urban/suburban site on Long Island, NY during both summer and winter. We use the resulting seasonal data to understand the sectoral and chemical composition of the emissions and elucidate the factors controlling their variability. Our results clarify the importance of different source sectors for urban atmospheric chemistry and identify priorities for inventory improvement.

## RESULTS

### Strong seasonality in urban VOC fluxes

The EC measurements (see Materials and Methods for details) reveal strong seasonality in urban reactive carbon fluxes ($F_{C}$). Figure 1 shows the VOC-carbon (VOC-C) fluxes and concentrations measured at 32.5 and 60 m above ground level on the Mineola tower during summer (12 July to 12 August 2023) and winter (1 to 20 February 2024). For clarity, we focus our analyses on the 32.5-m data and specify when referring to the 60-m observations. The VOC-C exchange is predominantly upward in both seasons, with mean summer fluxes (2.8 × 10^12^ atoms C cm^−2^ s^−1^) more than double those in winter (1.3 × 10^12^ atoms C cm^−2^ s^−1^). Species with predominantly downward exchange have a cumulative flux amounting to just 8% of the total emission magnitude, whereas for VOCs with predominantly upward exchange the downward contribution is <1%. Uncertainties associated with calibrations and the flux calculation could increase the computed $F_{C,\text{summer}}$ by up to 80% or decrease it by 31% and could increase $F_{C,\text{winter}}$ by 41% or decrease it by 18% (fig. S1, A and B; Materials and Methods) but would not alter the overall seasonal patterns. Like the fluxes, the mean VOC-C concentrations are 2× higher during summer [58 ppbC (parts per billion carbon)] versus winter (29 ppbC). However, within each season, the diel flux and concentration patterns differ strongly (Fig. 1, C and D), showing that concentration fluctuations do not primarily reflect changes in emission strength or timing.

**Fig. 1.**
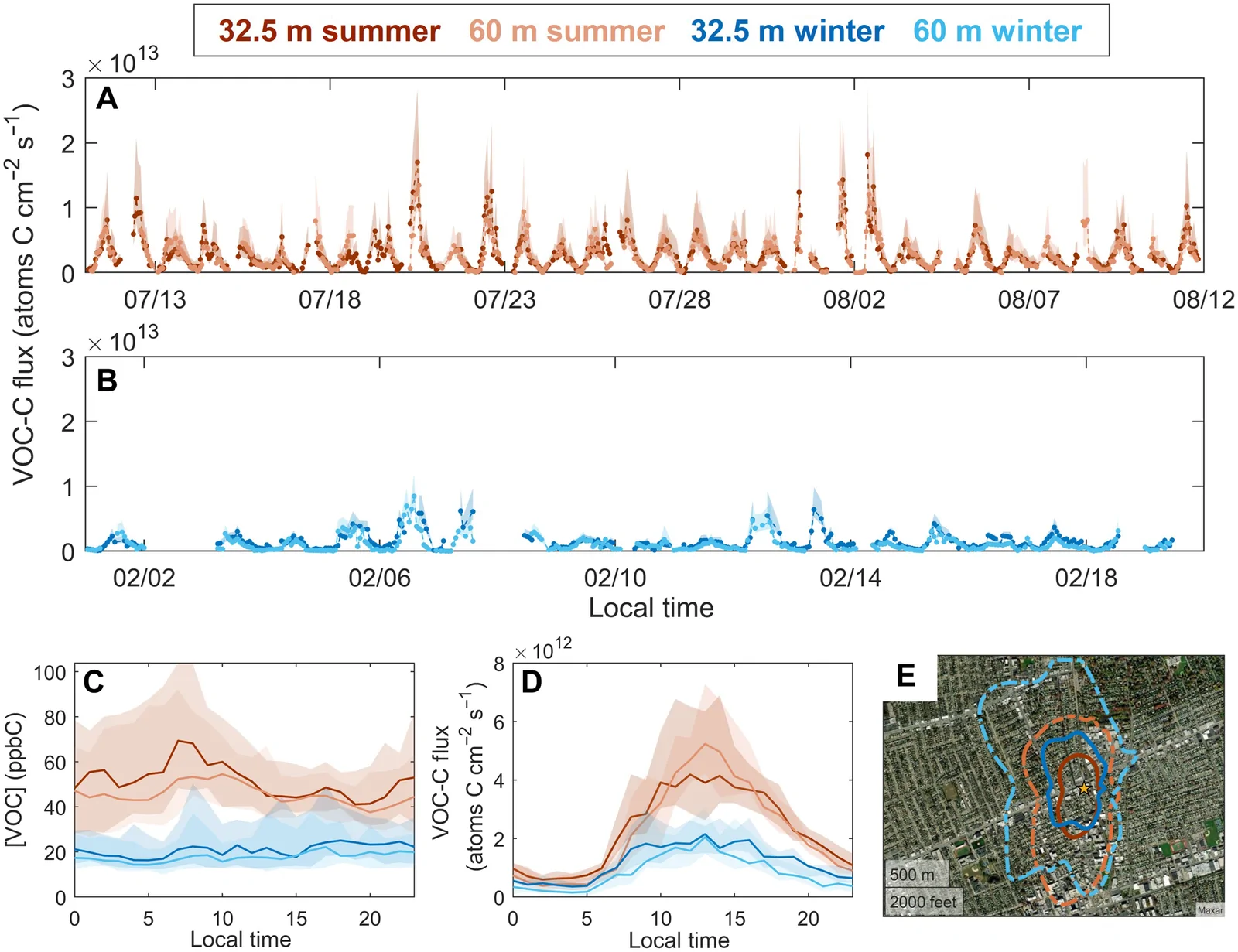
Seasonal and diel patterns in urban VOC fluxes. Plotted are the VOC-carbon (VOC-C) fluxes measured from the observatory in Mineola, NY at 32.5 and 60 m during (**A**) summer and (**B**) winter. (**C** and **D**) Median diel concentration and flux profiles, respectively. (**E**) 70% cumulative flux footprints by height (solid: 32.5 m; dashed: 60 m) and season (*81*) overlaid on the Esri World Imagery basemap (*88*). Shaded regions in (A) and (B) show propagated uncertainty bounds, whereas those in (C) and (D) show interquartile ranges about the diel median. The map image is the intellectual property of Esri and is used herein under license. Copyright 2026 Esri and its licensors. All rights reserved.

The total VOC-C fluxes measured at 60 m are lower (by an average of 8% in summer and 27% in winter) than those at 32.5 m. This modest amount of vertical flux divergence mainly arises from the differing footprints between the two heights (Fig. 1E), spatial heterogeneity in emissions in the surrounding landscape, and possible transport/advection effects. The flux differences by height thus provide a measure of representativeness for the Mineola observations within the urban/suburban mosaic. Chemical flux divergence is unlikely to be important for the measured VOCs as the characteristic mixing timescale (given by *H*/u_*_, where *H* is the height and u_*_ is the daytime friction velocity) (*35*, *46*) between 32.5 and 60 m is just ∼55 s.

We explore the implications of the above VOC exchange ($F_{\text{VOC},i}$) for urban oxidant chemistry by also quantifying the OH reactivity fluxes ($F_{\text{ROH}}=\sum k_{\text{OH}+\text{VOC},i}F_{\text{VOC},i}$; cm s^−2^) (*22*). The derivation applies literature-based VOC + OH rate coefficients (*47*–*49*) to the corresponding VOC fluxes; for species with known molecular formulae but unknown structure, we estimate $k_{\text{OH}+\text{VOC}}$ using a published parameterization that is based on carbon and oxygen number (*50*). By applying the same parameterization to species with known structures and rate coefficients, we infer a factor of 3 uncertainty in the derived $k_{\text{OH}+\text{VOC}}$ values (*21*) for structurally unresolved compounds. This could shift the $F_{\text{ROH}}$ values by up to 2.2× but would not fundamentally change their seasonal cycle (fig. S1, C and D).

Figure 2 shows that the primary contributors to the net molar and reactivity fluxes are distinct and vary by season. Ethanol (C_2_H_6_O) is the largest individual component of the VOC-C emissions, accounting for 22% of the total summer flux and 28% of the total winter flux. The top 5 emitted species are consistent across seasons and include (in addition to ethanol) methanol (CH_4_O), toluene (C_7_H_8_), acetone (C_3_H_6_O), and C8 aromatics (C_8_H_10_). Overall, the 10 most-emitted species account for 69% (summer) and 88% (winter) of the overall VOC-C exchange, reflecting the greater number of species with detectable fluxes in summer (126) than in winter (50). Biogenic VOCs are prominent during summer, particularly on a reactivity basis. Isoprene (C_5_H_8_; corrected for interferences as described in Materials and Methods) comprises the largest reactivity flux during summer but is absent during winter. As with the molar fluxes, most of the measured reactivity flux is provided by a relatively small number of compounds, with just 10 species accounting for 73% of the total during summer and 84% during winter. Thus, most of the urban VOC-C and OH reactivity emissions derive from species that are commonly represented in chemical transport models (CTMs) and in emission inventories, indicating the potential for computationally tractable model schemes that are nonetheless capable of accurately representing urban VOCs.

**Fig. 2.**
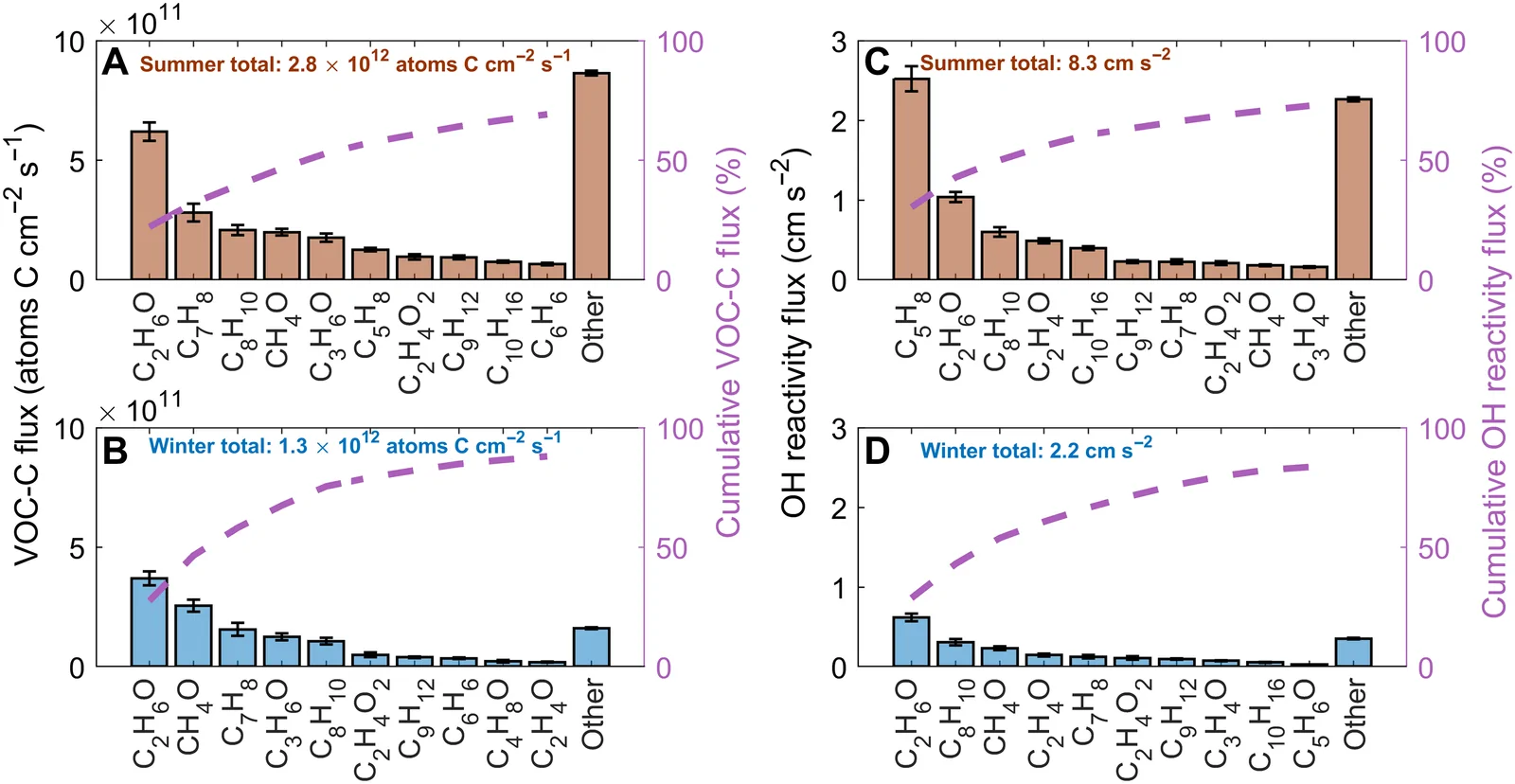
Composition of observed urban VOC fluxes. Top VOCs contributing to the total observed molar fluxes (**A** and **B**) and OH reactivity fluxes (**C** and **D**). Values reflect measurement period averages, with error bars indicating 95% confidence intervals. Dashed lines (scaled to the right-hand axes) show the cumulative contribution of the top 10 VOCs to the respective summed flux.

Although PTRMS measurements have low sensitivity to linear and branched alkanes, the above conclusions are unlikely to be substantially altered by any resulting VOC-C or OH reactivity flux underestimates. In particular, by combining summertime CO fluxes from this study with previously reported alkane:CO emission ratios (*51*), we estimate that unresolved acyclic alkanes (<C12) could increase the summertime VOC-C fluxes by ∼25%, with over half of this addition arising from just three compounds (*i*-pentane, propane, and ethane) that are themselves widely represented in inventories and air quality models. Meanwhile, the estimated alkane source changes the total OH reactivity fluxes by just ∼5% because of their comparatively low reaction rates.

### Evaporative and temperature-dependent processes drive VOC flux variability

We use nonnegative matrix factorization (NNMF; see Materials and Methods) to characterize the underlying sectors contributing to the measured fluxes. The resulting factor assignments are interpreted as dominant emissions categories rather than pure source profiles as some degree of mixing is expected when sources covary in space and time. Attribution is based on multiple lines of evidence including compound composition, temporal behavior, co-emitted tracers, and spatial footprints. During summer, the analysis reveals five separate factors (Fig. 3) that are dominated by (i) traffic, (ii) biogenic and residential emissions, (iii) generalized area-source VCPs, (iv) point-source VCPs, and (v) cooking sources. The emission “fingerprint” (Fig. 3A) quantifies the contribution of each VOC (plus CO and CO_2_) to the total variance for each factor and shows a chemically distinct profile in each case. Additional NNMF details are provided in the supporting information, which also delineates the top species-level contributors to the VOC-C and OH reactivity fluxes (figs. S2 to S5).

**Fig. 3.**
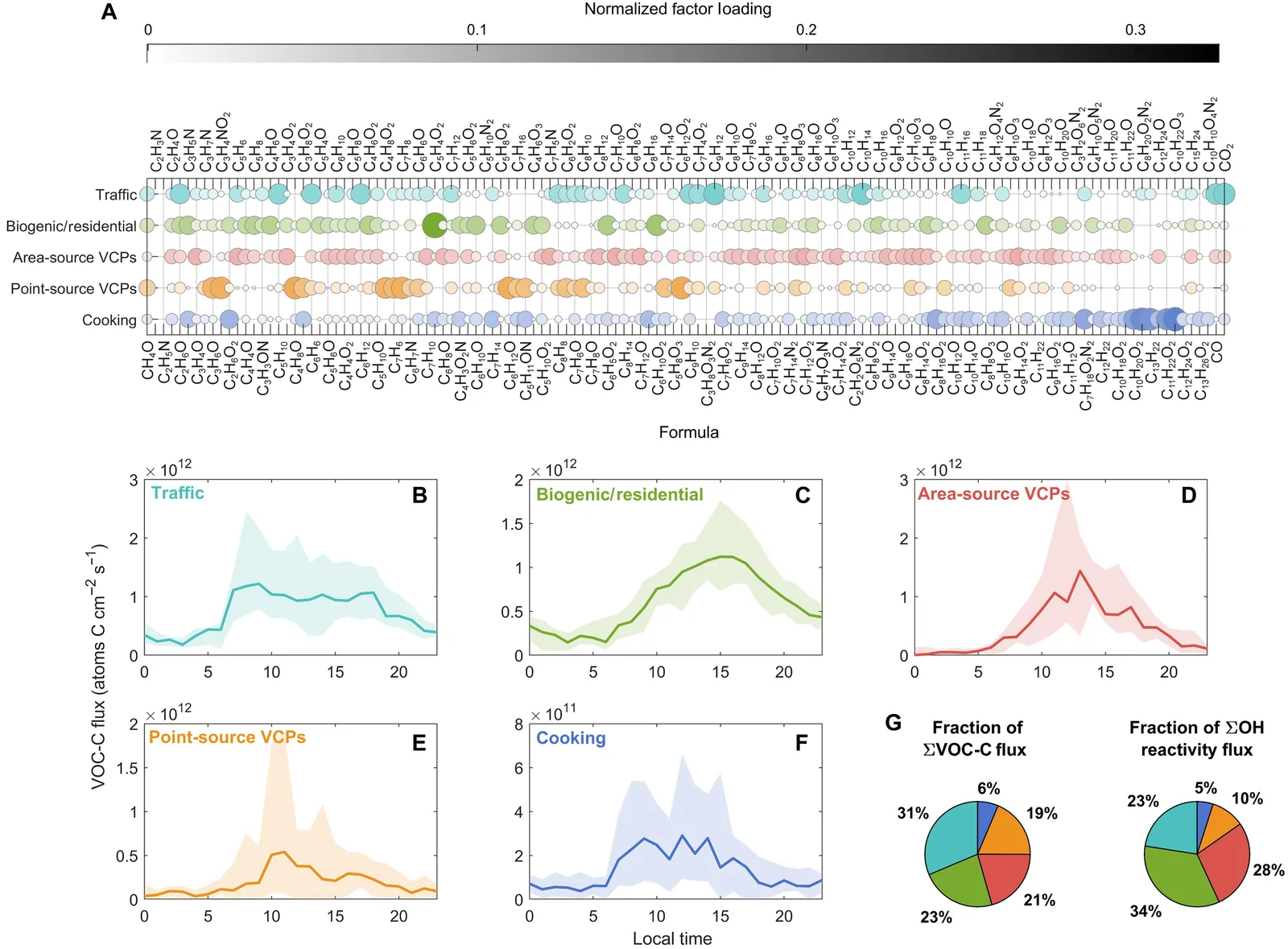
Composition of urban VOC fluxes during summer. Plots show sectoral contributions to the measured fluxes as determined by NNMF for summer 2023. (**A**) Contribution of each species to the total variance for each factor. Circle radius and shading are proportional to the NNMF factor loading (*H*) for each species within each factor. (**B** to **F**) Diel flux profiles for each factor (median and interquartile range). (**G**) Fractional contribution of each flux factor to the total VOC-C and OH reactivity fluxes.

The top factor driving summer VOC fluxes is dominated by traffic-related fossil fuel combustion ($F_{C,\text{traffic}}$) and has a characteristic diel profile with two rush hour peaks near 8 and 17 LT (local time) (Fig. 3B). Key contributors include known traffic tracers such as benzene, ethanol, styrene, C8 to C11 aromatics, and cycloalkanes. Twenty-three percent of the monoterpene VOC-C flux was attributed to the traffic factor, consistent with a previous study showing that traffic emissions can make a substantial contribution to urban monoterpene concentrations (*52*). The toluene:benzene flux ratio for this factor (2.2) falls within the range of concentration ratios (0.9 to 3) that have been reported for mobile sources (*53*, *54*). This factor exhibits a larger oxygenated VOC:BTEX ratio than the mobile source factor reported by Gkatzelis *et al.* (*12*) (44% versus 22%), likely reflecting some degree of mixing from sources that covary with traffic activity within the tower footprint. The $F_{C,\text{traffic}}$ factor also has the highest CO and CO_2_ fluxes of any source sector, supporting its fossil fuel attribution. Flux footprints during peak $F_{C,\text{traffic}}$ influence periods were concentrated to the northwest (fig. S6, A to D), encompassing high-traffic and idling areas near the Jericho Turnpike and Willis Avenue intersection. The timing of the $F_{C,\text{traffic}}$ morning increase and evening decrease matches traffic volume data from the closest Department of Transportation’s Travel Monitoring Analysis System site (DOT TMAS Site 341, 5.8 km NW of the tower), but $F_{C,\text{traffic}}$ displays more prominent rush hour peaks (fig. S7A). The sharp weekend decline in traffic emissions (when $F_{C,\text{traffic}}$ is ∼2× lower; fig. S8A) further indicates that the roads within our flux footprint function to a substantial extent as commuter corridors.

Landscaped residential areas contribute substantial VOC emissions through both biogenic sources and emissions from homes, and are collectively encompassed in the second NNMF factor ($F_{C,\text{bio}/\text{res}}$). Lines of evidence supporting this combined biogenic/residential attribution include (i) fluxes that were greatest from the residential area to the immediate southwest (fig. S6, E to H); (ii) the inclusion of known biogenic VOCs such as isoprene, monoterpenes, and methyl vinyl ketone + methacrolein (C_4_H_6_O); and (iii) the inclusion of furanoids such as furan (C_4_H_4_O), furfural (C_5_H_4_O_2_), furfuryl alcohol (C_5_H_6_O_2_), and 5-methylfurfural (C_6_H_6_O_2_), which are known tracers of building material degradation. Furfural is the strongest individual contributor to $F_{C,\text{bio}/\text{res}}$ variance, consistent with observations from residential attics and with its previous identification as an abundant indoor VOC (*19*, *55*). Although furans and furfural can also be associated with biomass burning, residential wood combustion is regulated in New York City and is not a major heating source in the surrounding region. More broadly, the overall temporal behavior, source footprint, and species composition of this factor are more consistent with combined biogenic and residential emissions than with combustion sources such as biomass burning or cooking-related activity.

The third NNMF factor has a chemical composition consistent with a source dominated by generalized VCP emissions (area-source VCPs; $F_{C,\text{AVCP}}$). The contributors to this factor span a wide range of VOCs (Fig. 3A), including oxygenated terpenoids and correlating species (C_10_H_16_O, C_10_H_18_O, and C_7_H_10_O), monoterpenes (C_10_H_16_), sesquiterpenes (C_15_H_24_), cyclic ketones (C_5_H_8_O and C_7_H_12_O), furaneol (C_6_H_8_O_3_), vanillyl alcohol (C_8_H_10_O_3_), octanal (C_8_H_16_O), and nonanal (C_9_H_18_O). The emissions likely arise from multiple sources, including personal care products, cleaning products, paints, and flavor additives. Although octanal and nonanal are not generally considered direct tracers of VCP formulations, their inclusion in this factor likely reflects covariance with VCP emissions and other built-environment sources, including indoor surface chemistry (*55*), emissions from building materials and furnishings (*56*, *57*), and temperature-dependent emissions from urban surfaces (*33*). We find that isoprene and monoterpene emissions are associated with both the area VCP and biogenic source factors (fig. S9).

Evaporation drives much of the $F_{C,\text{AVCP}}$ variance, as expected for VCPs. The factor correlates strongly with measured latent heat fluxes when the footprint is over urban hardscapes (pavement, asphalt, and rooftops), whereas that correlation is much weaker over suburban landscapes (lawns and trees) (Fig. 4A). The difference arises because latent heat is dominated by evaporation over hardscapes but reflects both evaporation and transpiration over suburban landscapes with plants. The hardscape-dominated region to the northwest of the tower exhibits stronger area VCP fluxes (fig. S6, I to L) and also has enhanced surface-atmosphere temperature gradients as inferred from the dimensionless temperature scale ($T_{*}$, calculated as the ratio of sensible heat flux to u_*_, which approximates $T_{\text{surface}}-T_{\text{air}}$) (*58*). Specifically, $T_{*}$ values are higher (by 19% on average) over the urban hardscape than they are over the suburban region, and the area VCP flux factor correlates more strongly with $T_{*}$ (*r*^2^ = 0.35; fig. S10) than it does with $T_{\text{air}}$ (*r^2^* = 0.19; Fig. 4B). Normalizing $F_{C,\text{AVCP}}$ by u_*_ does not strengthen this $F_{C,\text{AVCP}}-T_{*}$ relationship, indicating that the fluxes are not limited by transport but instead are primarily driven by surface temperature–dependent volatilization. The apparent temperature dependence of the area-source VCP factor (β = 0.08 to 0.16; Fig. 4B) is greater than would be expected solely from equilibrium vapor-pressure effects, suggesting that the observed response reflects factor-level flux behavior and coupled surface-atmosphere exchange processes rather than a pure volatilization signal.

**Fig. 4.**
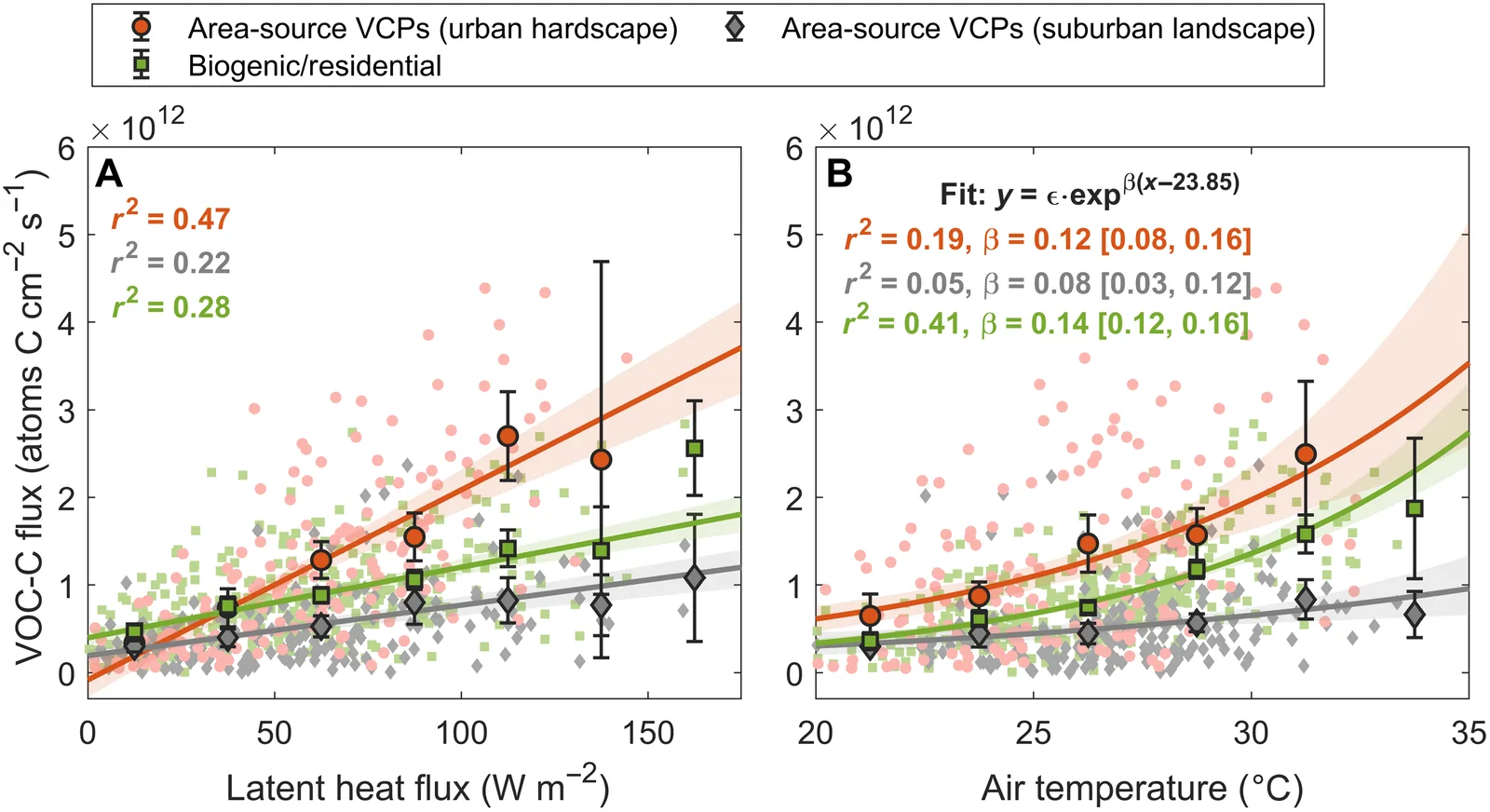
Environmental drivers of area-source VCP and biogenic/residential fluxes during summer. Area VCP ($F_{C,\text{AVCP}}$) and biogenic/residential ($F_{C,\text{bio}/\text{res}}$) VOC emissions are plotted as a function of (**A**) latent heat flux and (**B**) air temperature. Data are plotted separately for urban hardscapes and for suburban landscapes. Solid lines shows the fitted linear (A) or exponential (B) relationship between fluxes and latent heat or air temperature, with the 95% bootstrapped confidence intervals plotted as the shaded area and the corresponding parameters listed inset. Binned data points include 95% confidence intervals around the mean.

By comparison, the biogenic/residential fluxes vary exponentially with air temperature, and the strength of that dependence (β = 0.11 to 0.19; Fig. 4B) is in line with standard biogenic emission parameterizations (*26*) and with prior biogenic and attic emission studies (*19*, *22*–*24*, *59*). Emissions for this factor do not vary by day-of-week (fig. S8B) because they are controlled by temperature and surface characteristics (e.g., leaf/house area) rather than by day-to-day human activity. $F_{C,\text{bio}/\text{res}}$ correlates with *T*_air_ (*r*^2^ = 0.41) but shows little relationship with T_*_ (*r*^2^ < 0.1) or with latent heat, in contrast to $F_{C,\text{AVCP}}$. Thus, air temperature more closely reflects the processes driving biogenic/residential VOC emissions, which include warming of leaf or attic surfaces and indoor-outdoor transport.

The fourth factor ($F_{C,\text{PVCP}}$) is attributed to point-source VCPs and has strong contributions from common solvent species including acetone (C_3_H_6_O), acetic acid (C_2_H_4_O_2_), toluene (C_7_H_8_), xylenes (C_8_H_10_), phenol (C_6_H_6_O), and *n*-butyl acetate (C_6_H_12_O_2_) (Fig. 3A). These fluxes originated primarily from the north, aligning with industrial businesses including plumbing, auto repair, metal restoration, and lawn care operations (fig. S6, M to P). The toluene:benzene molar flux ratio for this factor (14) is consistent with reported source concentration ratios for solvent-use emissions (>8.8) (*54*, *60*–*62*). The point-source VCP emissions are highly variable and drive the sporadic maxima in the total $F_{C}$ time series seen in Fig. 1A. These occasional $F_{C,\text{PVCP}}$ peaks do not correlate with any meteorological variables, reflecting episodic anthropogenic activity without clear diel or weekend-weekday patterns (fig. S8D).

The fifth summer flux factor ($F_{C,\text{cooking}}$) is tied primarily to commercial cooking emissions, in particular of frying-related compounds that include octanoic acid (C_8_H_16_O_2_), decanoic acid (C_10_H_20_O_2_), undecanoic acid (C_11_H_22_O_2_), and linear alkenes (heptene, C_7_H_14_; octene, C_8_H_16_) (*35*). Emitted compounds also include food preservatives (benzoic acid, C_7_H_6_O_2_; propylene glycol, C_3_H_8_O_2_) and nitrogen-containing compounds (e.g., C_3_H_5_N, C_5_H_11_ON, and C_7_H_18_O_2_N_2_) that can originate from roasting or frying of plants or animals (*63*–*65*). The $F_{C,\text{cooking}}$ factor features a larger contribution from high-molecular-weight compounds than is the case for the other emission sectors (fig. S11). Although octanal and nonanal are commonly reported cooking emissions (*16*, *66*), this source only accounted for 15% of their total emitted molar flux during our study (fig. S9). Enhanced octanal and nonanal concentrations did occur during periods with elevated cooking organic aerosol (fig. S12), consistent with a measurable but secondary contribution from cooking emissions (*67*). The low proportion may reflect specific restaurant facilities and cooking practices or the fact that these *n*-aldehydes also have substantial VCP and residential sources. The flux footprint during peak $F_{C,\text{cooking}}$ periods centered over a cluster of restaurants to the north-northwest, which include a diner, a Peruvian restaurant, a Chinese restaurant, a churrascaria, and a fast-food burger restaurant (fig. S6, Q to T). The cooking emissions are sustained throughout the daytime (Fig. 3F), with weekday breakfast (∼8 LT) and lunch (∼12 LT) peaks and a single afternoon peak (∼15 LT) on weekends, reflecting regional dining patterns (fig. S8E).

Measurements from winter 2024 also reveal five flux factors, reflecting the same VOC-C source sectors seen during summer (Fig. 5). However, whereas VCPs are the largest summertime source, traffic predominates during winter when it accounts for roughly half of the emitted VOC-C mass. This $F_{C,\text{traffic}}$ factor is characterized by many of the same tracers seen during summer (e.g., benzene, C9 to C10 aromatics, alkenes, and cycloalkanes), and it also features the highest CO_2_ contribution across sectors (Fig. 5A). Flux footprints during peak $F_{C,\text{traffic}}$ periods are similar between seasons, as are the $F_{\text{toluene},\text{traffic}}:F_{\text{benzene},\text{traffic}}$ ratios (winter 2.9; summer 2.2). The total traffic VOC-C emissions are ∼28% lower during winter (fig. S13), which is similar to the seasonal traffic decrease measured at the nearby TMAS site (22%). The wintertime $F_{C,\text{traffic}}$ diel profile lacks distinct rush hour peaks, and this may reflect the seasonal reduction in commuter activity observed at the nearby TMAS site.

**Fig. 5.**
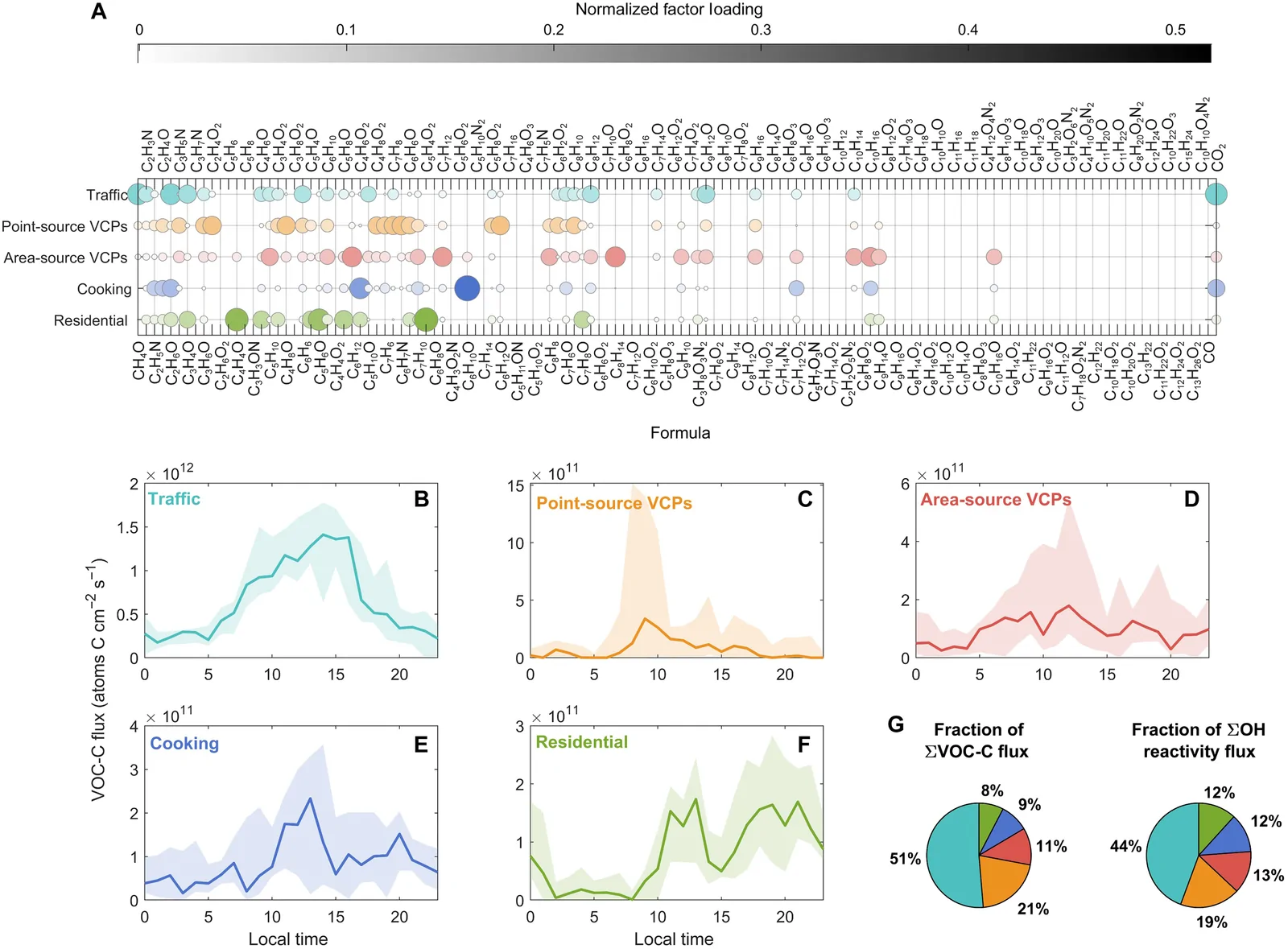
Composition of urban VOC fluxes during winter. Plots show sectoral contributions to the measured fluxes as determined by NNMF for winter 2024. (**A**) Contribution of each species to the total variance for each factor. Circle radius and shading are proportional to the NNMF factor loading (*H*) for each species within each factor. (**B** to **F**) Diel flux profiles for each factor (median and interquartile range). (**G**) Fractional contribution of each flux factor to the total VOC-C and OH reactivity fluxes.

Nontraffic VOC-C emissions during winter are associated with (in order of importance) point-source VCPs ($F_{C,\text{PVCP}}$), area-source VCPs ($F_{C,\text{AVCP}}$), cooking ($F_{C,\text{cooking}}$), and residences ($F_{C,\mathrm{res}}$). Compared to summer, the wintertime VCP and cooking emissions contain fewer high-mass compounds, reflecting the temperature-driven reduction in evaporation. $F_{C,\text{PVCP}}$ retains many of its associated species from summer (toluene, acetic acid, phenol, and C8 aromatics), along with its northerly footprint (fig. S14, E to H) and a $F_{\text{toluene},\text{PVCP}}:F_{\text{benzene},\text{PVCP}}$ ratio indicative of solvent use (11, versus 14 in summer). As before, the area VCP emissions are distributed over a broad range of VOCs (including monoterpenes, monoterpene oxides, styrene, and <C9 alkenes) but with total emissions less than half those in summer.

Cooking and residential emissions are chemically consistent with the corresponding summer sources and are characterized by nitrogen-containing species and by furanoids, respectively. Afternoon and evening increases in $F_{C,\mathrm{res}}$ (Fig. 5F) likely reflect periods of occupancy and home heating, including emissions associated with indoor surface chemistry. With winter winds, residential influences peak to the northwest, whereas the strongest cooking and fragrance emissions originate from southern and southwestern residential areas rather than from the mixed residential/commercial footprint seen in summer (fig. S14). This spatial shift away from restaurant emissions aligns with the absence of frying VOCs in winter and with a diel profile marked by midday and evening peaks that match home cooking patterns.

Our measurements show a pronounced seasonal shift in the dominant sources of urban VOC emissions. During summer, the temperature-dependent VCP and biogenic/residential fluxes contribute 63% of the total VOC-C fluxes and 72% of the reactivity fluxes, compared to just 40 and 44% in winter. Conversely, traffic and cooking sources increase from 37% of VOC-C fluxes and 28% of OH reactivity in summer to 60 and 56% in winter. A recent study has found that temperature-dependent fluxes account for most of the emitted reactivity in urban regions, and this is typically attributed to biogenic sources (*28*). Here, we find that surface temperature changes also modulate the volatilization of anthropogenic VCPs, and this has major impacts on the seasonality of urban atmospheric chemistry.

### Anthropogenic emission inventories do not account for VOC flux seasonality

Next, we use the observed fluxes to test state-of-the-art inventory estimates from the GReenhouse gas And Air Pollutants Emissions System (GRA^2^PES) and assess implications for air quality prediction. GRA^2^PES provides harmonized anthropogenic flux estimates for CO_2_ and air quality gases on a 4-km grid across the contiguous US (*68*); for this analysis, we focus on VOCs and include only those GRA^2^PES species that are detectable by PTRMS (see Materials and Methods). We note that the observed flux footprint (∼2 km^2^) is smaller than the 4 km–by–4 km GRA^2^PES grid cell (fig. S16) and thus places greater emphasis on the commercial, transportation, and residential land uses near the tower. Comparison of the tower grid cell to the surrounding 3 × 3 GRA^2^PES neighborhood shows that the local VOC-C flux differs by only 3% from the neighborhood mean and is thus broadly representative of the region. Neighboring grid cells nevertheless exhibit moderate spatial variability (coefficient of variation = 42%), consistent with the notion that inventory spatial heterogeneity may contribute to some inventory-observation differences.

Because GRA^2^PES is purely anthropogenic, we remove the biogenic influence from the summertime observations by excluding the combined biogenic/residential flux factor and by omitting isoprene, whose attribution to biogenic versus area VCPs is uncertain. Removing the biogenic contribution has a small effect on the VOC-C fluxes, but it reduces the mean summertime anthropogenic OH reactivity fluxes (without isoprene) from 5.8 to 4.2 cm s^−2^. For consistency, we also remove the residential contribution from the observed winter fluxes.

The resulting comparison (Fig. 6) shows that GRA^2^PES fails to capture the observed VOC emission seasonality: Measured anthropogenic VOC-C and reactivity fluxes decrease by 1.6× and 2.3×, respectively, from summer to winter, whereas the corresponding inventory predictions change by just ∼4% (Fig. 6 and fig. S17). As a result, the GRA^2^PES VOC-C emissions agree with the summer observations to within 40% but overestimate the winter fluxes by ∼2.5×. On the other hand, we find that the bulk chemical composition of emitted VOCs is well represented in GRA^2^PES, with the observed O/C (summer: 0.29; winter: 0.42) and H/C (summer: 2.0; winter: 2.4) flux ratios bracketing the corresponding inventory values (O/C = 0.36; H/C = 2.3). Previous work showed that oxygenated species accounted for most emitted VOCs in Innsbruck, Austria, with a fractional contribution (0.56) far exceeding inventory predictions (0.27) (*35*). In Mineola, this fraction is even higher (0.81 to 0.90) but is well represented in GRA^2^PES (0.94), underscoring the importance of regionally specific emission information.

**Fig. 6.**
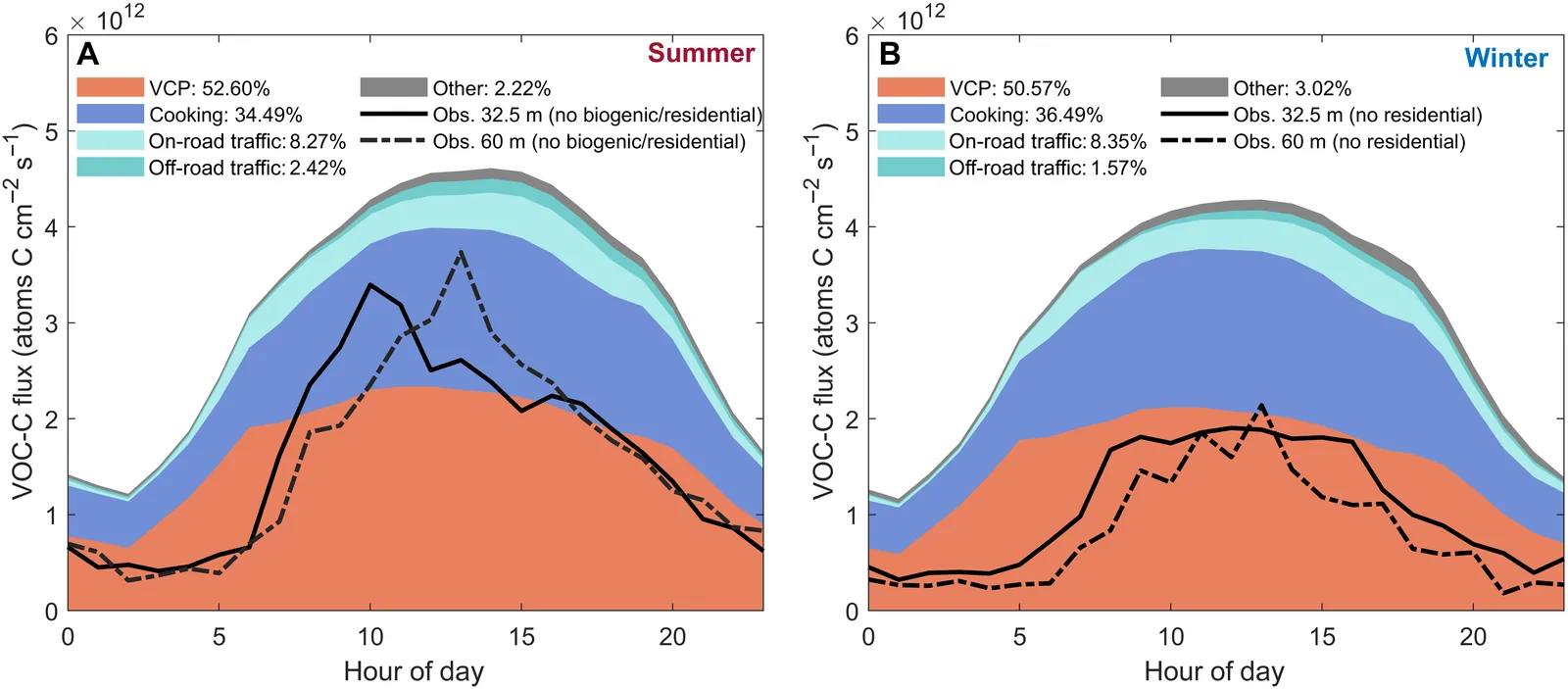
Measured versus modeled urban VOC fluxes. Plotted are the VOC-C diel flux profiles as predicted and observed for Mineola, NY during summer (**A**) and winter (**B**). In each case, GRA^2^PES inventory estimates (stacked colors) are compared to fluxes measured at 32.5- and 60-m height with biogenic/residential contributions removed.

Sector-specific comparisons reveal variable inventory performance across sources. VCPs and cooking together account for >88% of the GRA^2^PES VOC-C fluxes in both seasons, but this is primarily driven by emissions of ethanol and acetone, which are dramatically overestimated (collectively by >1 × 10^12^ atoms C cm^−2^ s^−1^ or 3×). Although the inventory VCP emissions agree with the observed values ($F_{C,\text{AVCP}}$ + $F_{C,\text{PVCP}}$) to within 42% during summer, they are 3.5× too high in winter. Cooking VOC-C emissions in the GRA^2^PES inventory are up to 9.1× larger than we observe, reflecting an order-of-magnitude overestimate of cooking-related ethanol fluxes. This discrepancy may partly arise from the fact that the model emissions are scaled by population density rather than by restaurant density (*68*). Ethanol emissions also depend strongly on cooking ingredients: Vegetables and tofu release more ethanol during cooking than does meat [e.g., tofu stir-fry emitted 3–4× more ethanol than chicken stir-fry during the HOMEChem study (*69*)]. These results suggest that inventories should consider the spatial distribution of cooking activity and regional differences in cooking style and ingredients. Meanwhile, traffic VOC-C emissions (on-road + off-road) are underestimated by ∼3× in both seasons. The discrepancy arises primarily due to an underestimate in the traffic contributions for species that are included in the inventory (e.g., ethanol, xylenes, and toluene) rather than from missing compounds. The lack of seasonality in the observed (and predicted) traffic fluxes is consistent with the modest winter reduction in traffic volume reported by TMAS and suggests that the inventory bias above reflects a systematic underestimate of emission factors, rather than a misrepresentation of their temperature-dependence or of underlying activity levels.

We further investigate the above model-measurement differences by examining the species-level biases in terms of their sectoral drivers. For each compound, we quantify the contribution of VCPs, cooking, or traffic to the total inventory error as

$$f_{\text{bias},i,j_{0}}=\frac{|F_{i,\text{obs},j_{0}}-F_{i,\text{inv},j_{0}}|}{\sum_{j=1}^{N_{\text{sectors}}}|F_{i,\text{obs},j}-F_{i,\text{inv},j}|}$$ (1)

where $F_{i,\text{obs},j}$ and $F_{i,\text{inv},j}$ are the observed and inventory VOC-C fluxes for compound *i* and sector *j*, respectively. The fractional bias $f_{\text{bias},i,j0}$ thus partitions the total inventory disparity across sectors and identifies the dominant source of error for each compound. Results plotted in Fig. 7 show that VCPs (red circles) and cooking emissions (blue squares) dominate the model-measurement flux discrepancies in both seasons. Toluene emissions are underestimated in GRA^2^PES due to a misrepresentation of the VCP source, whereas ethanol emissions are overestimated because of the cooking bias mentioned earlier. Methanol emissions are up to 13.5× too low in GRA^2^PES; the discrepancy is primarily attributed to traffic and is more pronounced during winter, possibly reflecting cold start emissions at low temperatures (*70*).

**Fig. 7.**
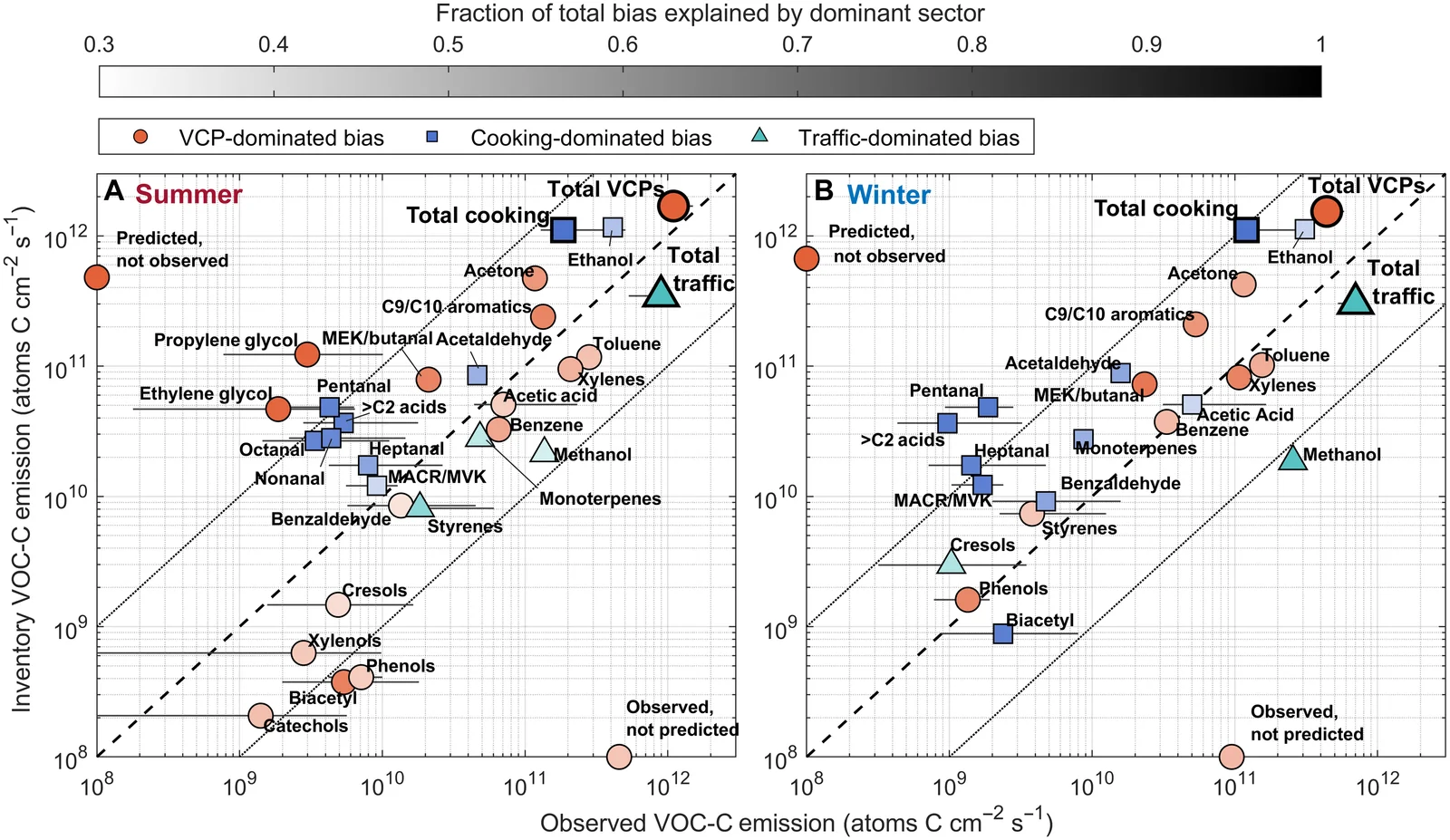
Predicted versus measured urban VOC fluxes by species and sector. Results are plotted for summer (**A**) and winter (**B**) and encompass the 27 VOCs that are both included in the GRA^2^PES inventory and detected by PTRMS. Marker shapes and colors indicate the dominant source of inventory bias, and the shading denotes the fraction of the total bias that is due to that source. Observed species that are missing from the inventory are shown along the *x* axis, whereas inventory species that are detectable by PTRMS but were not present are shown along the *y* axis. Dashed lines indicate the 1:1 relationship, and dotted lines indicate order of magnitude differences. Species-specific uncertainty bars show propagated measurement uncertainties. Uncertainty bars for the total VCP, traffic, and cooking sources indicate the 5th to 95th percentile uncertainty range (Materials and Methods).

We find that discrepancies between observed and inventory VOC-C fluxes arise from two distinct categories: (i) compounds with measurable fluxes that are not included in GRA^2^PES and (ii) compounds included in the inventory but without detectable emissions. Specifically, 22 and 9% of the total measured summer and winter anthropogenic VOC-C flux, respectively, arises from compounds absent in GRA^2^PES, whereas 25% of the total inventory flux arises from compounds that do not have detectable emissions. In both categories, VCPs dominate, underscoring the large uncertainties in modeling their emissions. A range of VCP species with modest (e.g., catechols and xylenols, bias ≤ 10^9^ atoms C cm^−2^ s^−1^) to large (e.g., ethylene glycol and propylene glycol, bias = 10^10^ to 10^11^ atoms C cm^−2^ s^−1^) summertime inventory biases are not observed during winter (because lower surface temperatures suppress volatilization) but continue to be emitted in GRA^2^PES. Similar seasonal behavior is seen for the C8 to C9 *n*-aldehydes; here, the observed fluxes are primarily attributed to VCP sources, whereas wintertime emissions are undetectable due to the reduced volatilization. However, wintertime fluxes persist in GRA^2^PES where these compounds are assigned to cooking. Overall, the above results reveal a need in current inventories for better representation of seasonal and regional patterns of VOC emission variability, including the effects of surface temperature volatilization, cold start emission factors, and sector-specific activity patterns.

## DISCUSSION

Urban air quality forecasts require an accurate understanding of VOC sources and the environmental factors driving those emissions. We show that the VOC-C fluxes in an urban/suburban region vary strongly by season, with summer emissions more than twice (and OH reactivity fluxes >3.5×) those in winter. NNMF identified five main emission categories, with VCPs and traffic dominating the summer and winter VOC fluxes, respectively. By combining direct EC flux measurements with NNMF, footprint quantification, and meteorological analysis, this work provides a source attribution framework that offers a substantial advance over concentration-based methods.

Temperature-dependent processes drive the seasonal variability in urban VOC sources but are not well captured in inventories (*28*). During summer, elevated surface temperatures enhanced the evaporative emissions of VCPs and the biogenic emissions from plants, and these sources then accounted for ∼63% of the VOC-C flux and ∼72% of the emitted OH reactivity. This picture parallels recent results from Los Angeles where temperature-sensitive emissions accounted for ∼60% of summertime O_3_ and SOA formation potential (*28*). In wintertime New York, these sources were substantially reduced, and traffic became the dominant contributor because this flux was relatively stable across seasons (∼28% summer-to-winter decrease). Total VOC-C fluxes predicted by the GRA^2^PES inventory agree with the summertime observations to within 25% but are more than 2× too high during winter. Discrepancies arise from (i) uncertainties in the spatial scaling of commercial cooking emissions, which are allocated using population density rather than restaurant density, (ii) emitted species that are not accounted for in the inventory, (iii) inventory species that are detectable but not observed to be emitted, and (iv) the lack of a temperature-dependent volatilization framework that contributes to a VCP overestimate. Sector-level analysis further indicates that VCPs and cooking dominate inventory biases and that traffic VOC emissions are systematically underestimated. Residential and building material sources emit urban VOCs but are excluded from inventories and warrant further study.

This work adds to the emerging EC literature demonstrating that modern urban VOC emissions differ substantially from the transportation-dominant scenario represented in many historical inventories. Oxygenated compounds accounted for 81 to 90% of the emitted VOC flux in Mineola NY, in line with results from Innsbruck and Beijing where such species also comprised most of the measured VOC source (*35*, *43*, *71*). Ethanol was the most-emitted VOC at our site (22 to 28% of the total VOC-C flux) and was likewise among the dominant species detected in Innsbruck, Beijing, and Los Angeles (*28*, *43*). Source apportionment analyses indicate that these nontraditional emissions originate from a common set of source categories across cities. For example, VCPs in Beijing accounted for ∼50% of the measured VOC emissions compared to 24% from vehicle-related sources (*71*), whereas our NNMF analysis similarly identified VCPs as the largest summertime source category (∼40% of VOC-C emissions versus 31% from traffic). We further demonstrate here that urban VOC sources vary substantially by season, with traffic accounting for 51% of the Mineola total during winter as temperature-dependent VCP and biogenic/residential emissions declined.

Accurate VOC source attribution is essential for evaluating the air quality implications of different control strategies. For example, an inventory overestimate of cooking VOC emissions (as found here) might exaggerate the potential efficacy of restaurant exhaust scrubbers for reducing secondary pollutant formation. Meanwhile, accounting for the temperature dependence of anthropogenic VOC emissions should be a priority to enable more accurate O_3_ and aerosol predictions during urban heat waves (*67*). Incorporating temperature-driven parameterizations of evaporation into CTMs [e.g., (*34*)], and improving the representation of cold start emissions, are avenues for progress in this regard. The observed dependence of VCP emissions on surface temperature also suggests that urban heat-mitigation strategies, such as urban greening, may provide ancillary air quality benefits by reducing evaporative VOC emissions.

We focused here on anthropogenic VOC sources, but emissions from the urban biosphere are also important and poorly constrained (*15*, *28*). For example, a comparison of the FROG-NY biogenic VOC fluxes with GEOS-Chem/MEGAN predictions (*72*) (fig. S18) shows that, although MEGAN captures the observed seasonal pattern, it strongly overestimates the summertime biogenic isoprene, monoterpene, and sesquiterpene fluxes. The disparity highlights large remaining uncertainties in urban biogenic VOCs and the need for inventories that can better account for the land cover and environmental conditions encountered in cities.

The conclusions presented here reflect flux measurements and inventory comparisons from a single urban/suburban footprint in the New York City metropolitan area during one year. VOC sources, chemical speciation, temperature-volatility relationships, and factors that influence the release of indoor-origin VOCs (e.g., building ventilation and air exchange rates) vary across urban environments, and interannual variability in meteorology and activity patterns can modify the magnitude of seasonal contrasts. Our analysis is limited to VOCs that are detectable by PTRMS; the inventory comparisons used the same set of species for consistency, but findings therefore exclude some species that can be important contributors to urban reactivity (e.g., formaldehyde and alkanes). The degree to which the mechanisms driving flux variability is consistent across cities and years remains uncertain, highlighting the need for chemically comprehensive VOC flux measurements across a wider range of urban environments. Such efforts will be important for characterizing the dominant urban VOC species and sectors and for understanding their changes through time. Overall, our work demonstrates gaps in our current understanding of urban VOC emissions and identifies temperature as a key driver of seasonality in urban organic carbon sources.

## MATERIALS AND METHODS

### Field site

We made chemical and meteorological measurements from a cellular communications tower (*73*, *74*) during summer (9 July to 14 August 2023) and winter (1 to 20 February 2024) during the Fluxes of Reactive Organic Gases in New York (FROG-NY) study. The tower is sited in Mineola, NY (40.750°N,73.638°W; 33-m elevation), a densely populated urban/suburban area (4338 people km^−2^) ∼28 km downwind of New York City that is influenced by both local and regional emissions (*67*). The local footprint includes landscaped residential areas, a major highway, restaurants, and other commercial infrastructure; the average building height is ∼14 m. Medium and high-intensity developed land makes up 95% of the surrounding terrestrial area, comparable to or exceeding borough-scale averages for Manhattan (85%), Queens (81%), the Bronx (73%), and Staten Island (54%) (*75*) (fig. S19).

Gas-phase species measured during FROG-NY included VOCs (see the Supplementary Materials), O_3_, NO*_x_*, CO, CO_2_, and water vapor. An infrared gas analyzer (LI-7000, LI-COR Biosciences) was used to measure CO_2_ and water vapor concentrations at 10 Hz and was calibrated (every 3 days) via dynamic dilution of a CO_2_ cylinder standard [408 parts per million (ppm)] into zero air and (three times) with a dewpoint generator (LI-610 Portable Dew Point Generator, LI-COR Biosciences). CO was measured at 2 Hz with a cavity enhanced absorption spectroscopy instrument (OA-ICOS, Los Gatos Research), which was calibrated weekly via dynamic dilution of a CO cylinder standard (10 ppm) into zero air.

We sampled air at 32.5- and 60-m heights using custom-built, identical PFA inlets (Clayborn Lab; ID: 0.5″, length: 112 m) held at 40°C to minimize wall interactions and avoid condensation. The sampling inlets were mounted at the end of 1.8-m booms, directly below sonic anemometers (CSAT 3B, Campbell Scientific) measuring three-dimensional (3D) winds and air temperatures at 10 Hz. O_3_, NO*_x_*, aerosol composition and size distributions, and basic meteorological parameters were measured at the base of the tower as described elsewhere (*67*).

Chemical instrumentation was housed in an air-conditioned station at the tower base. We used a custom-built automated sampling manifold that cycled between the 32.5- and 60-m inlets every 45 min. Each inlet was backed by a rotary vane pump (model 1023, Gast Manufacturing), which maintained continuous air flow at 38 SLPM and an inlet pressure of 94 kPa immediately downstream of all instrumentation. All wetted sampling surfaces were either PFA or passivated stainless steel (Inertium, AMCX). The VOC, CO_2_, and water vapor measurements were sampled from the switching manifold, whereas the CO measurements were sampled continuously from the 32.5-m inlet. The above sampling system enabled direct quantification of net fluxes by EC for VOCs, CO_2_, latent heat, and sensible heat (at 32.5 and 60 m) and for CO (at 32.5 m).

### EC flux calculations

EC fluxes were calculated from the covariance of a given scalar (*X*) with vertical wind (*w*) for an ensemble of *n* measurements over an averaging period of 40 min (at 32.5 m) or 45 min (at 60 m) (*58*)

$$F=\bar{w^{\prime }X^{\prime }}=\frac{1}{n}\sum_{i=1}^{n}\left(w_{i}-\bar{w}\right)\left(X_{i}-\bar{X}\right)$$ (2)

where overbars indicate temporal averaging and primes indicate deviations from the mean. Scalar concentrations and winds were first despiked using the median absolute deviation method (*76*) and detrended by subtracting a linear fit for each averaging period. Wind fields underwent double coordinate rotation so that $\bar{w}$ = 0 for each averaging period (*77*). The lag time between the wind measurement (at the sonic anemometer) and downstream species detection was quantified on the basis of the maximum *w*-*X* cross-covariance (fig. S20); time-resolved lags derived in this way for ethanol (∼21 s) were applied to all VOCs due to the high signal-to-noise for this species.

We applied standard QA/QC procedures to ensure robust EC flux derivation. Flux uncertainties were calculated as 1.96× the SD of the outer 30 points within a 600-point window around the peak *w*-*X* cross-covariance (*78*). The resulting uncertainties averaged 10% for ethanol, 9% for benzene, 9% for isoprene, and 33% for toluene. Compounds with campaign-mean fluxes below the corresponding uncertainty level were removed from the analysis, leading to 126 and 50 VOCs with detectable fluxes for summer 2023 and winter 2024, respectively. Systematic errors associated with high-frequency attenuation from inlet damping, sensor separation, and instrument response were quantified via cospectral analysis and the empirical model of Horst (*79*), leading to cumulative positive corrections of 13% (32.5 m) and 6% (60 m) for the total VOC flux. Figure S21 visualizes these corrections based on the normalized *w*-*X* cospectra for sensible heat and select VOCs spanning a range of volatilities. The Supplementary Materials section describes other flux quality control measures used to reject periods with low shear-driven turbulence and nonstationarity (*80*).

Total propagated flux uncertainty accounted for uncertainties in instrument calibration and flux derivation. For directly calibrated species, the associated uncertainty was propagated on the basis of the certified accuracy of the calibration standard, the variability in calibration response, and the mass flow accuracy. Compounds without direct calibrations used uncertainty bounds from the observed range of PTRMS sensitivities across calibrated VOCs. Uncertainties in the EC calculations were derived fractionally by species and propagated with the calibration uncertainties before VOC-C flux summation.

We used a 2D flux footprint prediction model to assess the spatial contributions to observed fluxes (*81*). The model was constrained with assimilated meteorological parameters from the Goddard Earth Observation System Forward Processing product [GEOS-FP; (*82*)] based on local estimates of the urban canopy height (14 m) and roughness length (1 m).

### Nonnegative matrix factorization

We used NNMF (as implemented in MATLAB R2023b) to systematically investigate patterns of variability in the observed EC fluxes and to identify VOC emission sources. The method was applied to a time-series matrix ($\overset{⃡}{A}$) of VOC, CO, and CO_2_ fluxes at a given height, with each flux first normalized by its SD to prevent any individual species from dominating the solution. This *n* (number of timepoints) × *m* (number of species) matrix was apportioned into two nonnegative matrices: $\overset{⃡}{W}$ (*n* × *j*), representing a time series of length *n* for *j* factors, and $\overset{⃡}{H}$ (*j* × *m*), representing the contributions of all *m* species to each factor *j*, such that

$$\overset{⃡}{A}=\overset{⃡}{W}\times \overset{⃡}{H}+\overset{⃡}{E}$$ (3)

where $\overset{⃡}{E}$ is the residual error matrix. For each NNMF solution, the initial guesses for $\overset{⃡}{W}$ and $\overset{⃡}{H}$ were obtained from 50 replicate runs with a maximum of five iterations each. The resulting matrices initialized the final NNMF calculation, which used 100 iterations and 50 replicates. We selected the minimum number of factors (*j*) based on two validation metrics: (i) the root mean square residuals (*D*) between $\overset{⃡}{A}$ and $\overset{⃡}{W}\times \overset{⃡}{H}$ and (ii) the Bayesian information criterion (BIC) (*35*)

$$\text{BIC}=\text{log}\left({\left\|\overset{⃡}{W}\times \overset{⃡}{H}-\overset{⃡}{A}\right\|}^{2}\right)+j\frac{m+n}{\mathrm{mn}}\text{log}\left(\frac{\mathrm{mn}}{m+n}\right)$$ (4)

Both metrics imply six possible factors in each study period (based on a plateau in the derivative of *D* and the minimum in BIC; fig. S22), but because we observe splitting in the top 2 factors to produce a mixed-source category (fig. S23), we restrict the analysis to the five-factor solution.

We performed multiple analyses to characterize uncertainty in the NNMF solution. We first accounted for initialization sensitivity by generating 500 NNMF solutions with identical input matrices but different random seeds. Factors from each solution were matched using the species-composition matrix $\overset{⃡}{H}$ to account for differences in factor ordering. The base-case solution was then selected as the one with factor diel profiles that most closely aligned with the ensemble median (figs. S24 and S25). We next performed a bootstrap analysis to quantify uncertainties associated with the temporal sampling of our dataset. To this end, 500 NNMF solutions were generated on the basis of randomized nonoverlapping 24-hour block resamples of the original dataset, each using the base-case initialization selected via the seed analysis. Factors were again matched using $\overset{⃡}{H}$, and the resulting distributions were combined with those from the seed ensembles (1000 total realizations) to define an overall 5 to 95% uncertainty range for our source attribution.

The NNMF positivity requirement was met by only including VOCs with predominantly upward fluxes; downward fluxes amounted to 7% of the upward magnitude and were excluded. Most downward fluxes were carried by six species with the following formulae: CH_4_N_2_H^+^, C_3_H_7_NH^+^, C_2_H_4_O_3_H^+^, C_3_H_8_O_3_N_2_H^+^, C_5_H_4_O_4_H^+^, and C_3_H_6_O_4_N_2_H^+^.

From the NNMF results, we calculated the total VOC-C flux for each factor j ($F_{C,j}$)

$$F_{C,j}=\sum_{i=1}^{i=m}\sigma_{i}\times W_{j}\times H_{j,i}\times C_{i}$$ (5)

where $\sigma_{i}$ is the flux SD and *C* is the carbon number for VOC species *i*. We use observed meteorological data, flux footprints, and inferences of human activity patterns to assign a source identify to each factor.

### GReenhouse gas And Air Pollutants Emissions System

We obtained monthly, day-of-week, and diel anthropogenic VOC emission estimates by sector from the NOAA/NIST GRA^2^PES v1.0 inventory (*68*, *83*). GRA^2^PES provides data at a 4 km–by–4 km resolution and includes 67 individual and lumped VOCs across 18 emission sectors. It integrates prior inventories including NEI 2017 (*84*), the Fuel-based Oil and Gas (FOG) inventory (*85*), the Fuel-based Inventory of Vehicle Emissions (FIVE) (*86*), the Volatile Chemical Products inventory (*10*), and observationally informed estimates for cooking emissions (*16*). All comparisons use the GRA^2^PES emissions for the grid cell containing the measurement site during February and July. The GRA^2^PES estimates used here were developed for year 2021, but we do not apply any subsequent scaling as New York’s anthropogenic VOC emissions are estimated to have decreased by only ∼1% from 2021 to 2023 (*87*). Of the 96 inventory species, we analyze the 46 VOCs that are measurable by PTRMS (table S1) and exclude alkanes, short-chain alkenes/alkynes, formaldehyde, and glycerol.

## Supplementary Materials

### This PDF file includes:

Supplementary Text Figs. S1 to S25 Tables S1 and S2 References
