# Supplementary material for: Surface temperatures drive strong seasonality in urban reactive carbon emissions
Source: Sci Adv. 2026 Oct 7;12(41):eaef9622. doi: 10.1126/sciadv.aef9622 (PMC13644763; doi:10.1126/sciadv.aef9622)
Supplement: Supplementary file 1 [file sciadv.aef9622_sm.pdf]

Science Advances
AAAS

Supplementary Materials for

Surface temperatures drive strong seasonality in urban reactive carbon emissions

Michael P. Vermeuel *et al.*

Corresponding author: Dylan B. Millet, dbm@umn.edu; Delphine K. Farmer, delphine.farmer@colostate.edu

Sci. Adv. **12**, eaef9622 (2026)
DOI: 10.1126/sciadv.aef9622

This PDF file includes:

Supplementary Text
Figs. S1 to S25
Tables S1 and S2
References

Supplementary Text

Volatile organic compound (VOC) measurements

VOCs were measured with a proton-transfer reaction mass spectrometer (PTRMS; PTR-QiTOF, Ionicon Analytik). The time-of-flight mass analyzer collected ions with a 35 μs extraction period (m/z 0-390.85) with spectra then coadded to 10 Hz and recorded. The mass axis was continuously calibrated using peaks for water vapor (m/z 21.022, $H_3{}^{18}O^+$), acetone (m/z 59.049, $C_3H_7O^+$), and an internal diiodobenzene standard (m/z 203.943, $C_6H_5I^+$ and 330.848, $C_6H_5I_2{}^+$). The drift tube was held at 3.8 mbar, 80°C, and 970 V to maintain E/N = 133 Townsend (Td). The resulting sensitivity and mass resolution averaged 2500 cps ppb^{-1} (cps: counts per second) and 4300 m/Δm for acetone, and 1100 cps ppb^{-1} and 3900 m/Δm for benzene.

The PTR-MS sampling followed a duty cycle with: 1) 45 min of sampling at 60 m, 2) 40 min at 32.5 m, 3) a 3 min instrument zero, and 4) a 2 min single-point calibration via dynamic dilution of a compressed VOC standard. Background PTR-MS spectra were generated by passing ambient air through a platinum bead catalyst (3.2 mm diameter; Shimadzu Corp.) heated to 380 °C. Four compressed gas cylinders containing ppm-level VOC mixtures (34 total VOC standards; **Table S2**) in zero air were used for calibration, with one cylinder used per calibration period. Four multi-point calibration curves were collected during each campaign: before and after deployment and twice during the measurement intensive. Sensitivities derived using the single-point field calibrations remained within 6% of the multi-point values, and we interpolated between these points to obtain 10 Hz background signals and calibration factors. All ion signals were normalized to 2×10^5 cps of $H_3{}^{18}O^+$ (normalized cps, ncps). These directly calibrated compounds accounted for 73% and 88% of the total observed VOC-C flux during summer and winter, respectively. Uncalibrated VOCs employed the measured sensitivity for toluene (1450 ncps ppb^{-1}), which had the median sensitivity and humidity dependence across all calibrated compounds. Similar generalized PTRMS quantification approaches for compounds lacking direct calibrations have been described previously (*21, 22*). We then applied the 10th and 90th percentiles of measured sensitivities for all VOCs (440–2300 ncps ppb^{-1}) to derive lower and upper uncertainty bounds for the sum of uncalibrated species.

We corrected the $C_5H_9{}^+$ signal (normally attributed to isoprene) for interferences from heptanal, octanal, and nonanal following Coggon et al. (*16*). This correction reduced the summertime isoprene signal by 47% (36% for the flux), and completely removed it in the winter. We also accounted for acetaldehyde ($C_2H_5O^+$) and benzene ($C_6H_7{}^+$) artifacts arising respectively from the fragmentation of ethanol and ethylbenzene, again following Coggon et al. (*16*). These corrections reduced the derived concentrations and fluxes by approximately 2% for acetaldehyde and by 7–8% for benzene. Humidity dependencies for the species-specific calibration factors were quantified three times (before, during, and after the study) by diluting VOC standards into zero air humidified to span the observed dew points (5–22 °C). The resulting corrections were propagated to the field data based on the measured $H_2O{\cdot}H_3O^+$ (m/z 37.028) to $H_3{}^{18}O^+$ ratios, and led to modest (<20%) changes in the derived calibrations.

The Ionicon Data Analyzer v2.2 (IDA; Ionicon Analytik) (*90*) and custom season-specific tables of ~1,350 peaks (m/z 13–372) were used to fit and integrate the observed mass peaks for the summer 2023 (43 measurement days) and winter 2024 (21 days) campaigns. Molecular formulae

were assigned to 780 of the 1,350 quantified ions using PTRwid (*91*). All subsequent data processing was performed in MATLAB (R2023b, MathWorks).

PTRMS sampling configuration
The PTRMS inlet was teed into a 1 m bypass (1/8” ID, PFA) that subsampled from a manifold switching between the 32.5 m and 60 m inlets. The bypass pressure was held at 880 mbar, allowing the instrument drift tube to draw a 90 sccm mass flow, and a supplemental bypass was used to draw an additional 250 sccm through the heated (80°C) 1.5 m PTRMS inlet (1/16” ID, PFA). An inline 47 mm diameter, 5-6 µm pore-size filter (Savillex™) was installed within the sampling bypass, with replacements every ~3-4 days. Filtration assemblies holding 90 mm PFA filters of 5–6 µm porosity were also installed at the end of each inlet, downstream of the PTRMS.

Flux quality control
The overall eddy covariance processing methodology follows our previously described PTRMS flux workflow (*22*) with modifications specific to the present urban sampling configuration. We calculated fluxes from the eddy covariance (EC) between the vertical wind velocity (*w*) and scalar concentrations (*X)* over each averaging interval (40 min at 32.5 m and 45 min at 60 m) (*58*):

$$F = \overline{w'X'} = \frac{1}{n}\sum_{i=1}^{n}(w_i - \overline{w})(X_i - \overline{X}) \quad \textbf{(ES1)}$$

where overbars denote temporal means and primes are departures from those means.

Prior to covariance calculations, wind and concentration time series were despiked using the median absolute deviation method (*76*) and detrended by subtracting a linear fit for each averaging period. Wind vectors then underwent double rotation to enforce $\overline{w} = 0$ (*92*). Scalar lag times were determined from maxima in *w*-*X* cross-covariance. Ethanol, which exhibited the highest and most robust *w*-*X* signal was used to define the representative lag time for routine processing (21 s; **Fig. S20**). This value agreed closely with the expected residence time of air within the sampling system and was representative of the dominant high-volatility VOCs. Slightly longer lags were seen for lower-volatility species like sesquiterpenes. To assess uncertainty associated with these lag differences, we additionally computed representative species-specific lag times from the median daytime cross-covariance maximum in each case. For sesquiterpenes, this yielded a 24 s lag (**Fig. S20**) and a 4% difference in the calculated flux. The median impact across all species was 1.9%. These species-specific sensitivities were incorporated as an additional uncertainty term in the reported VOC-C and OH reactivity fluxes, but contributed just 0.6% to the total VOC-C flux uncertainty.

Fluxes were excluded when any of the following quality control criteria were not satisfied:

1. Friction velocity (u_*) exceeded 0.10 m s^{-1},
   $$u_* = \left(\overline{w'u'}^2 + \overline{w'v'}^2\right)^{\frac{1}{4}} \quad \textbf{(ES2)}.$$
   with *u* and *v* respectively denoting the zonal and meridional wind speeds.
2. A stationarity test (*80*) indicated agreement between the mean flux of five equal-duration subperiods and the corresponding averaging period within 30%:

$$\left|1-\frac{\frac{1}{5}\sum_{i=1}^{5}\overline{w'X'}_{sub-period,i}}{\overline{w'X'}_{full\,period}}\right| \leq 0.3 \quad \textbf{(ES3)}$$

3. The campaign-mean flux for a given compound exceeded the corresponding campaign-mean flux uncertainty.

Spectral corrections

Attenuation of flux by inlet damping, instrument response, and sensor separation was corrected using an empirical model transfer function formulation (*79*). The measured flux (F_m) was converted to the corresponding unattenuated flux (F) using:

$$\frac{F_m}{F} = \frac{1}{1+(2\pi n_m \tau_c U/z)^{\alpha}} \quad \textbf{(ES4)}$$

where U is the mean horizontal wind speed (m s^{-1}), z is the measurement height (m) minus the displacement height (2/3 of the mean building height), τ_c is the characteristic system response time, and n_m and α are empirical constants for an unstable boundary layer (0.085 and 7/8, respectively).

Response times τ_c were determined directly from the measured cospectra by comparing normalized *w-X* cospectra with corresponding normalized sensible heat (*w-T*) cospectra, the latter of which were assumed to experience negligible attenuation. The response time corresponds to the frequency (cutoff frequency, f_c) where the measured *w-X* cospectrum decreases to $1/\sqrt{2}$ of the *w-T* cospectrum, and τ_c is then calculated as $\tau_c = 1/(2\pi f_c)$. Representative examples are shown in **Fig. S21A**. For benzene fluxes measured at 32.5 m during the summer, the observed response time was $\tau_c \approx 1$ s under mean daytime conditions ($\overline{U}$ = 3.8 m s^{-1}; z = 23.2 m) (**Fig. S21A**), corresponding to an average correction of 12%. Across the complete VOC dataset, spectral corrections increased the calculated net VOC fluxes by an average of 13%.

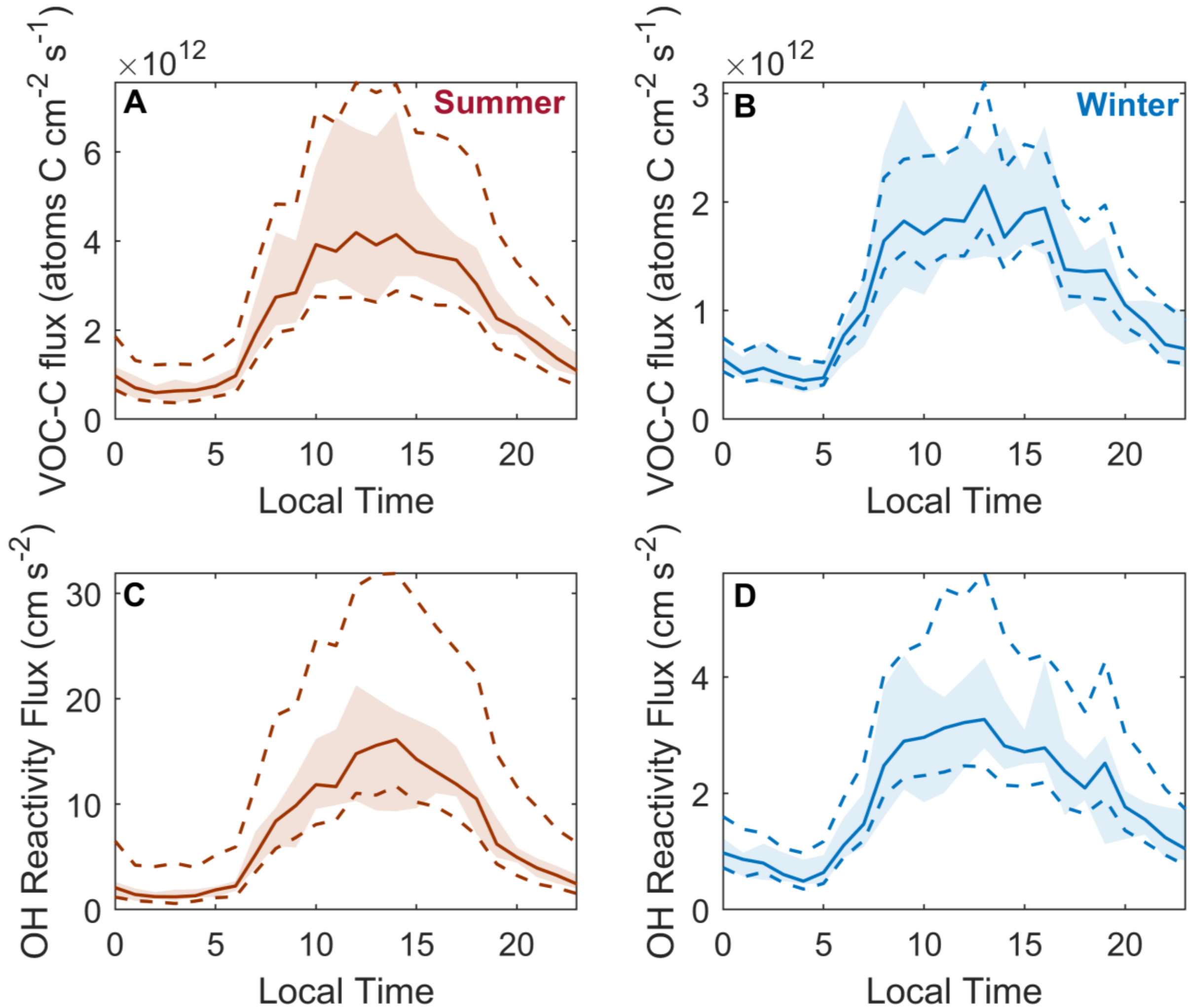

Figure S1: Median diel profiles for the VOC-C fluxes (A, B) and OH reactivity fluxes (C, D) as measured during summer and winter at 32.5 m. Shaded regions show the interquartile range about the median. Dashed lines indicate upper and lower calibration uncertainties.

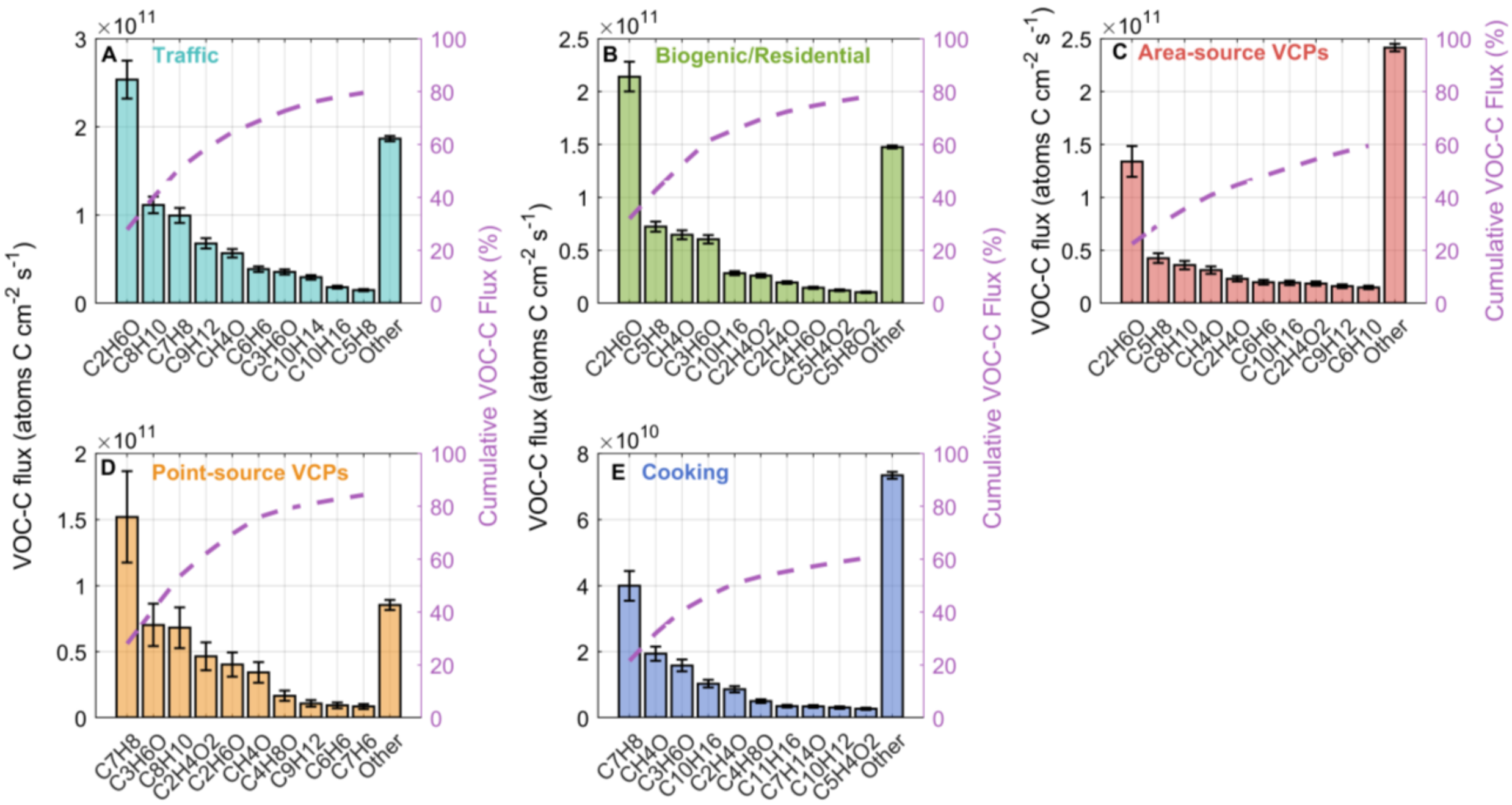

Figure S2: Top 10 VOCs contributing to the total carbon flux for each NNMF source factor during summer 2023. Values represent measurement period averages, with error bars indicating 95% confidence intervals. The dashed line shows the cumulative contribution of the top 10 VOCs to the respective summed flux.

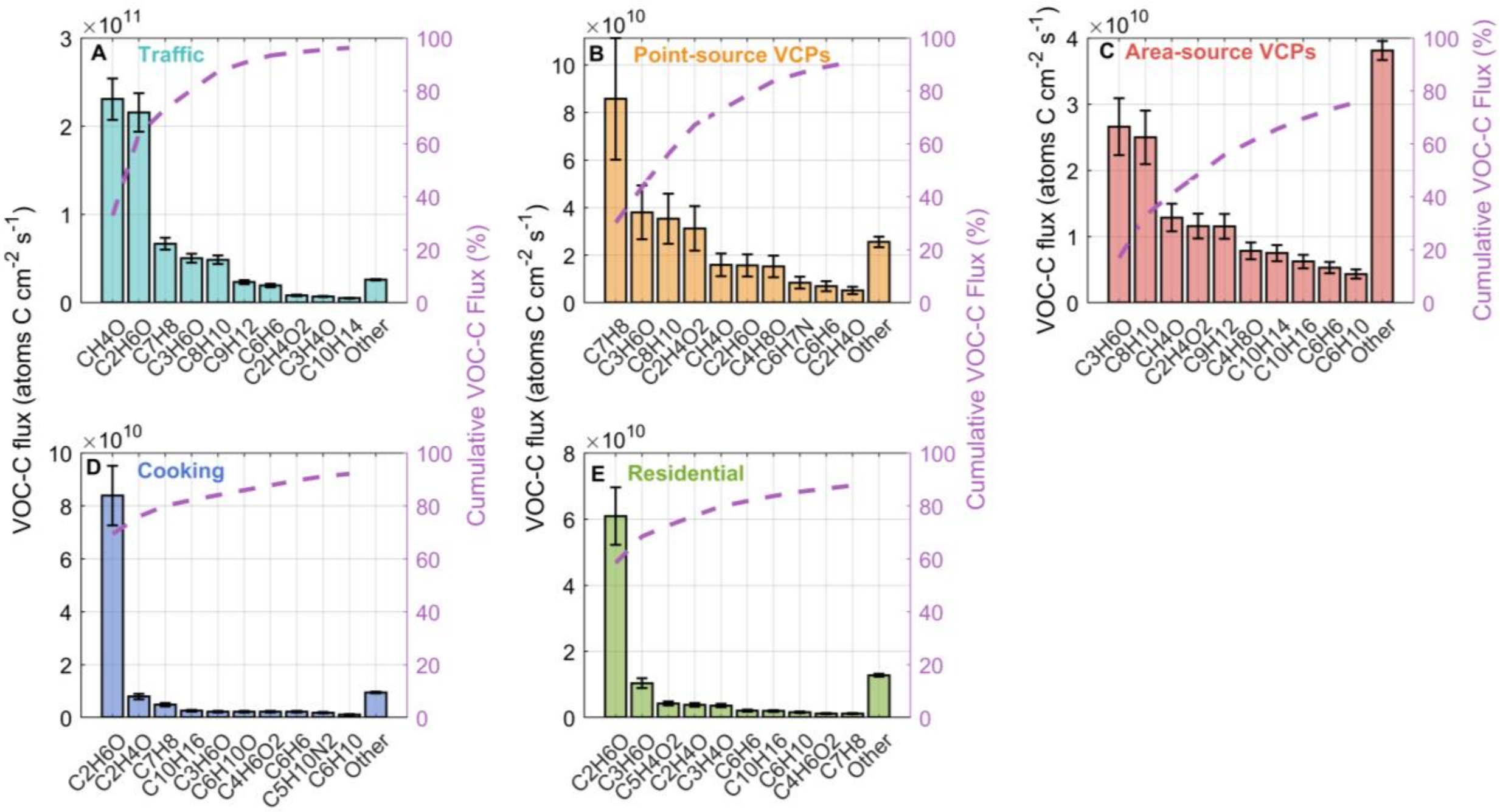

Figure S3: Top 10 VOCs contributing to the total carbon flux for each NNMF source factor during winter 2024. Values represent measurement period averages, with error bars indicating 95% confidence intervals. The dashed line shows the cumulative contribution of the top 10 VOCs to the respective summed flux.

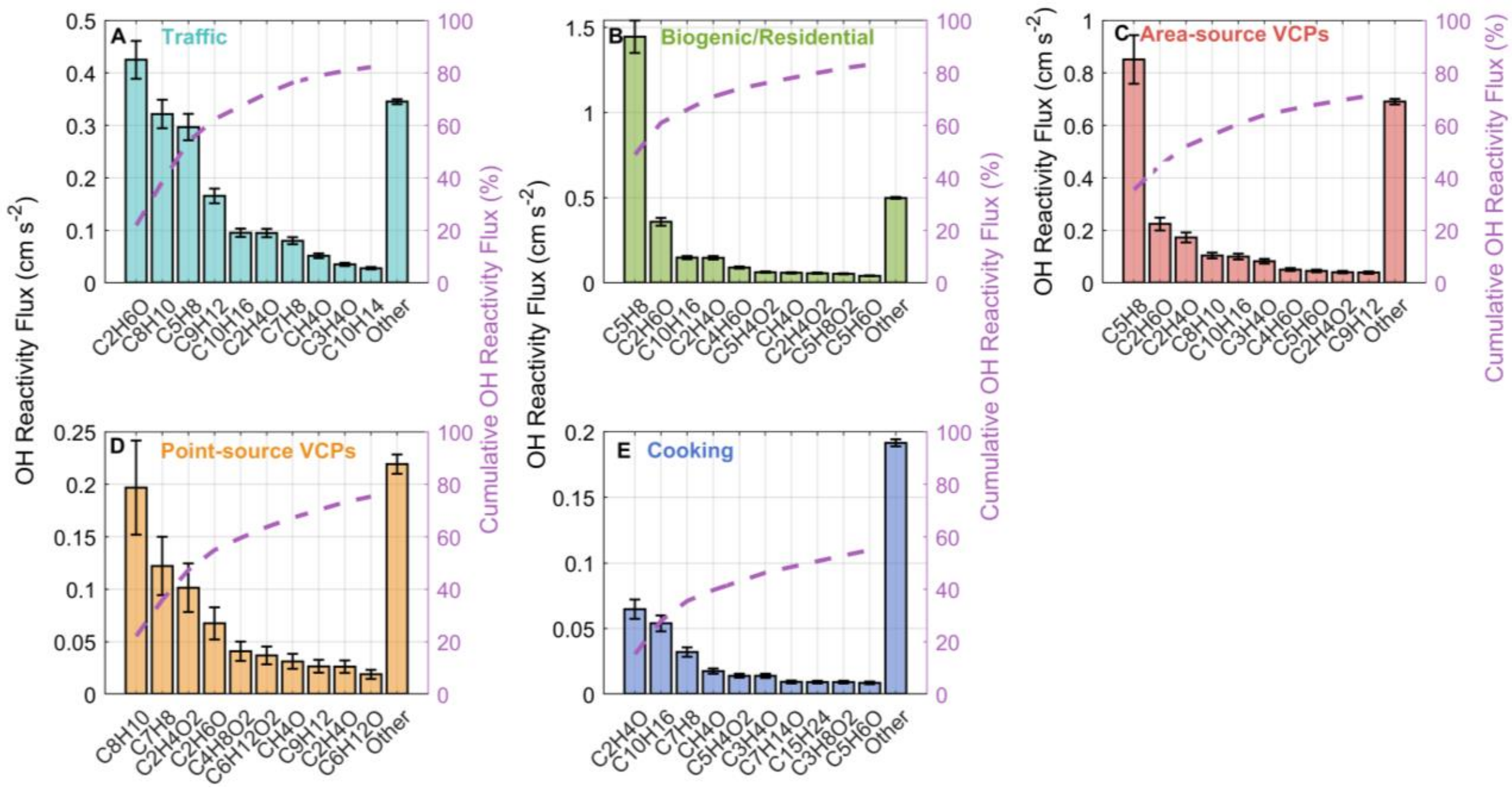

Figure S4: Top 10 VOCs contributing to the total OH reactivity fluxes for each NNMF source factor during summer 2023. Values represent measurement period averages, with error bars indicating 95% confidence intervals. The dashed line shows the cumulative contribution of the top 10 VOCs to the respective summed flux.

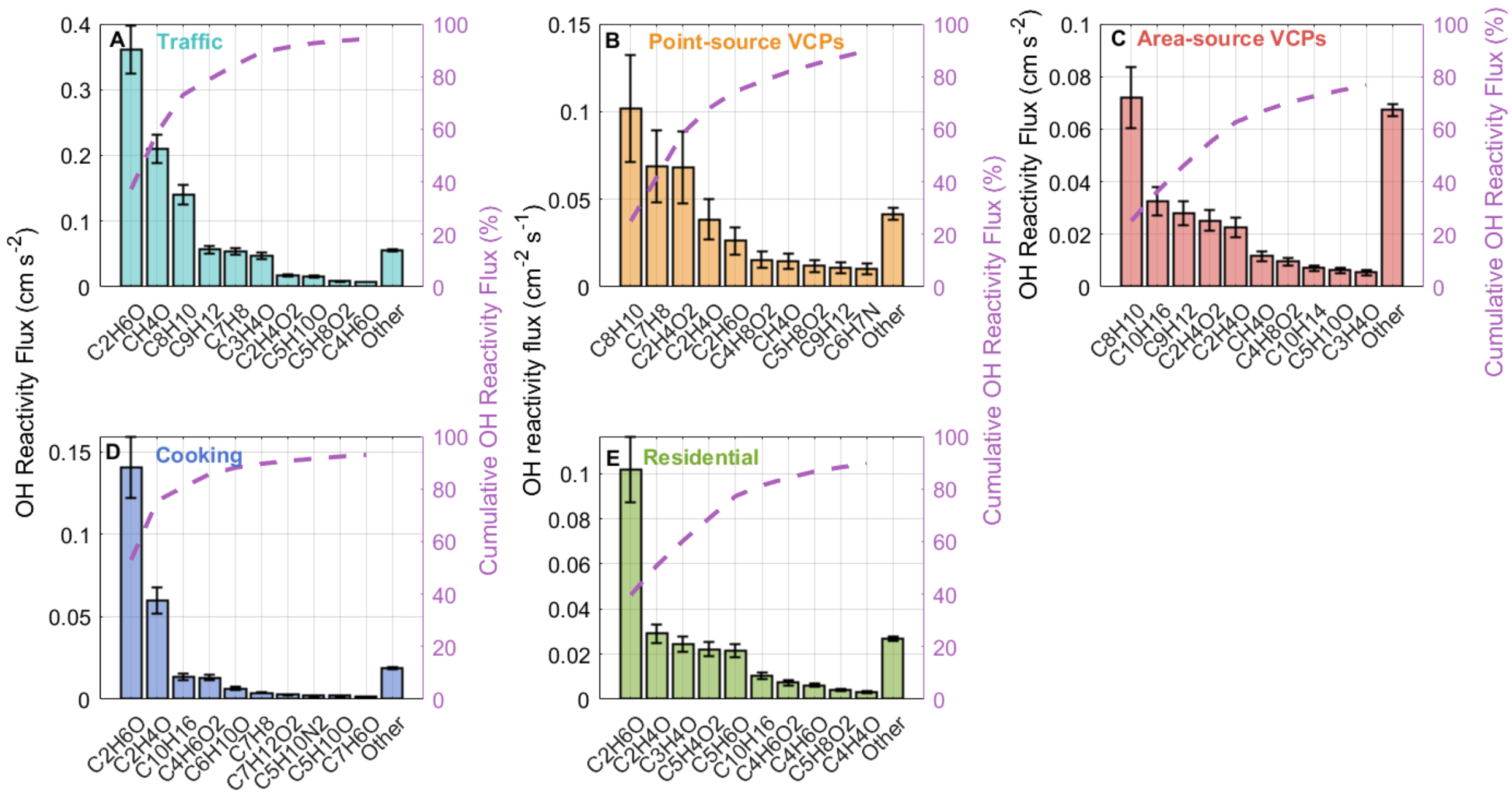

Figure S5: Top 10 VOCs contributing to the total OH reactivity fluxes for each NNMF source factor during winter 2024. Values represent measurement period averages, with error bars indicating 95% confidence intervals. The dashed line shows the cumulative contribution of the top 10 VOCs to the respective summed flux.

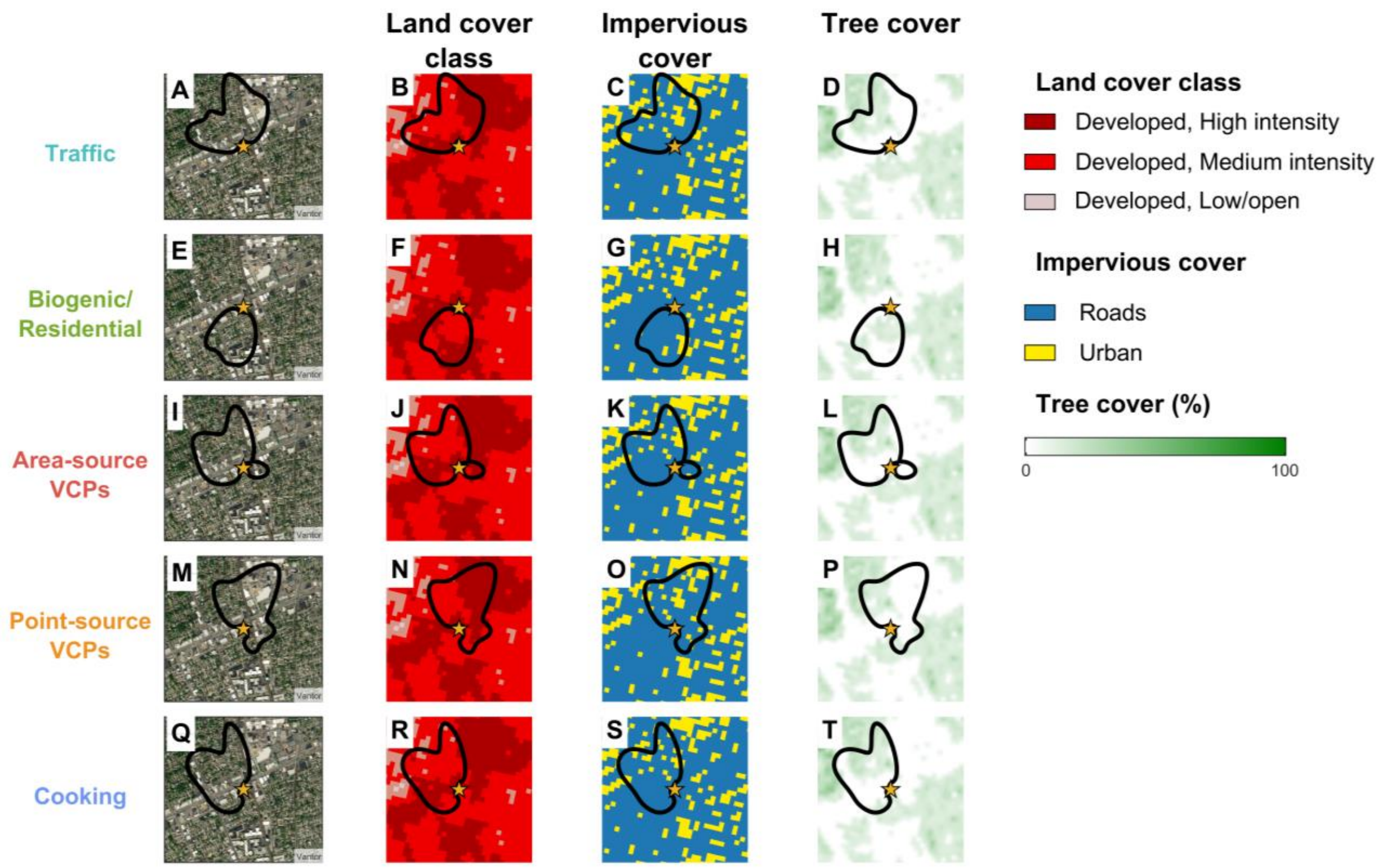

Figure S6: Cumulative 70% flux footprints during sampling intervals associated with the top 10th percentile for each NNMF source factor during summer. Footprints are overlaid on the Esri World Imagery basemap (*88*) (left column) and on National Land Cover Database 2023 products for land cover class, impervious cover, and tree cover (remaining columns) (*75*). Map image is the intellectual property of Esri and is used herein under license. Copyright © 2026 Esri and its licensors. All rights reserved.

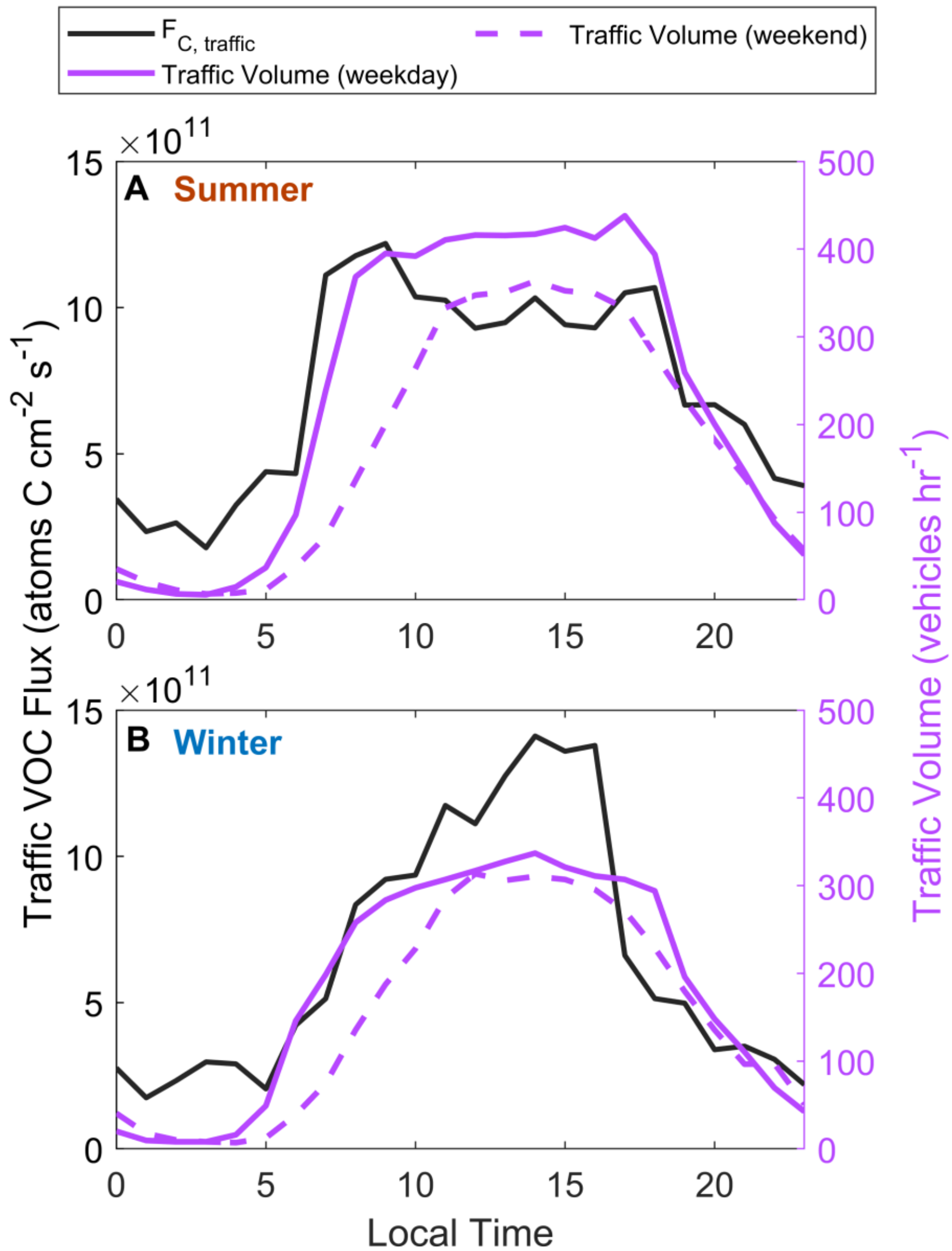

Figure S7: Measured traffic VOC fluxes as attributed by NNMF ($F_{C,traffic}$, solid black lines) compared to traffic volume for weekdays (solid purple lines) and weekends (dashed purple lines) from the U.S. Department of Transportation Travel Monitoring Analysis System (DOT TMAS Site 341) during summer (A) and winter (B).

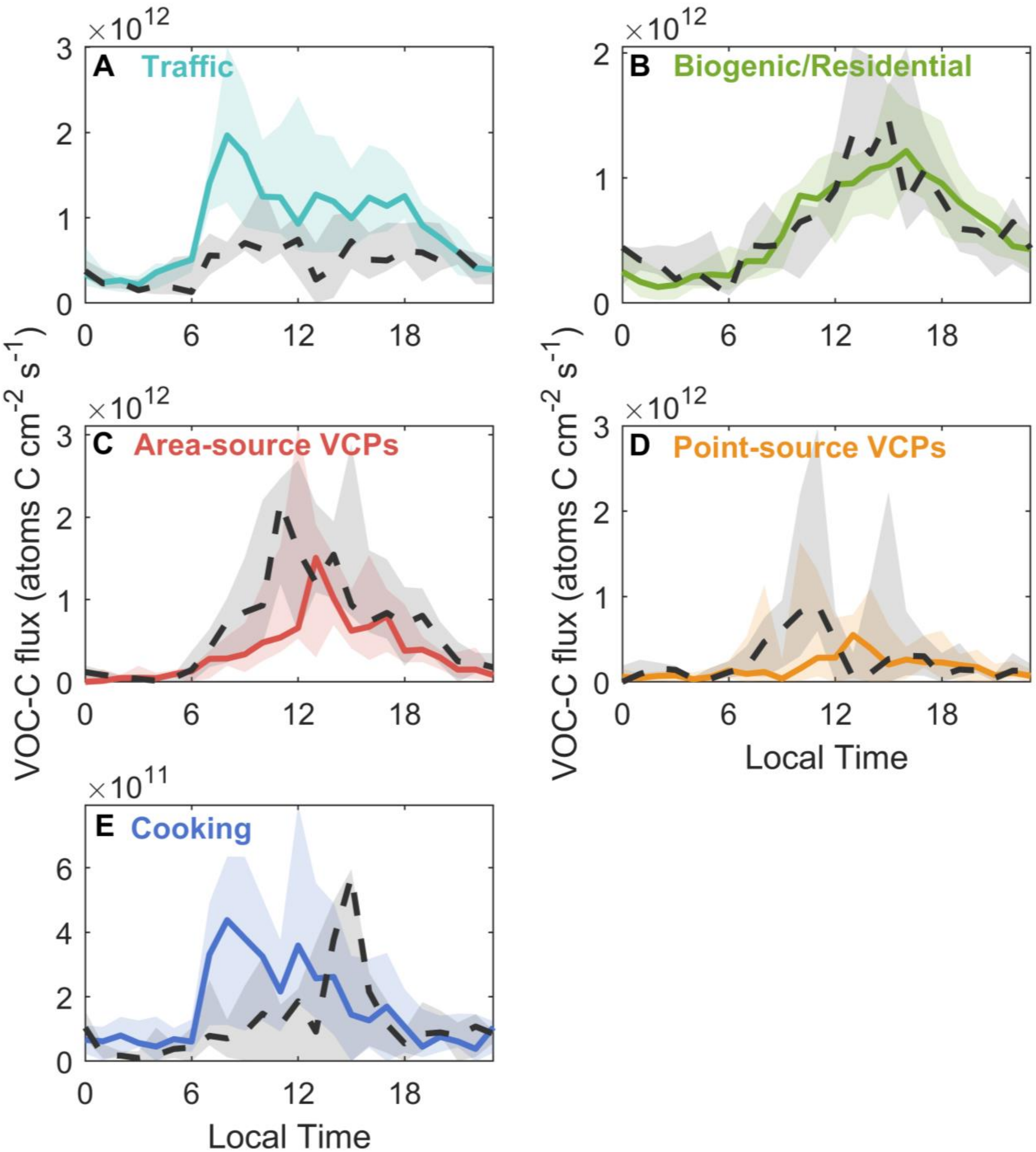

Figure S8: Median diel VOC-C flux profiles for each identified source factor on weekdays (colors, solid lines) and weekends (grey, dashed lines) during summer 2023. Shaded regions indicate interquartile ranges about the median. A winter weekday–weekend comparison was not performed due to the limited number of weekend sampling days.

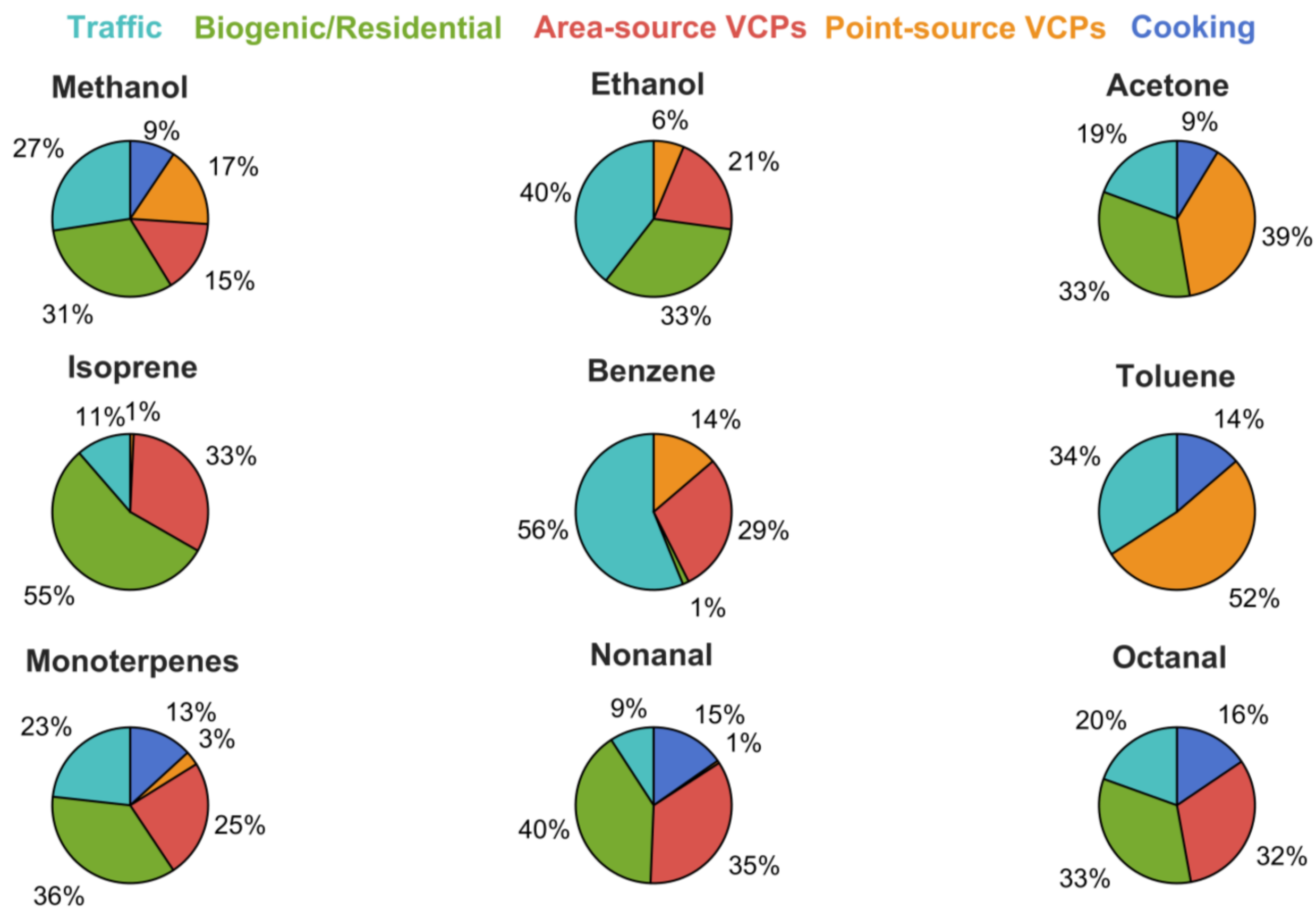

Figure S9: Mass attribution of select measured VOCs to the NNMF source factors for summer 2023.

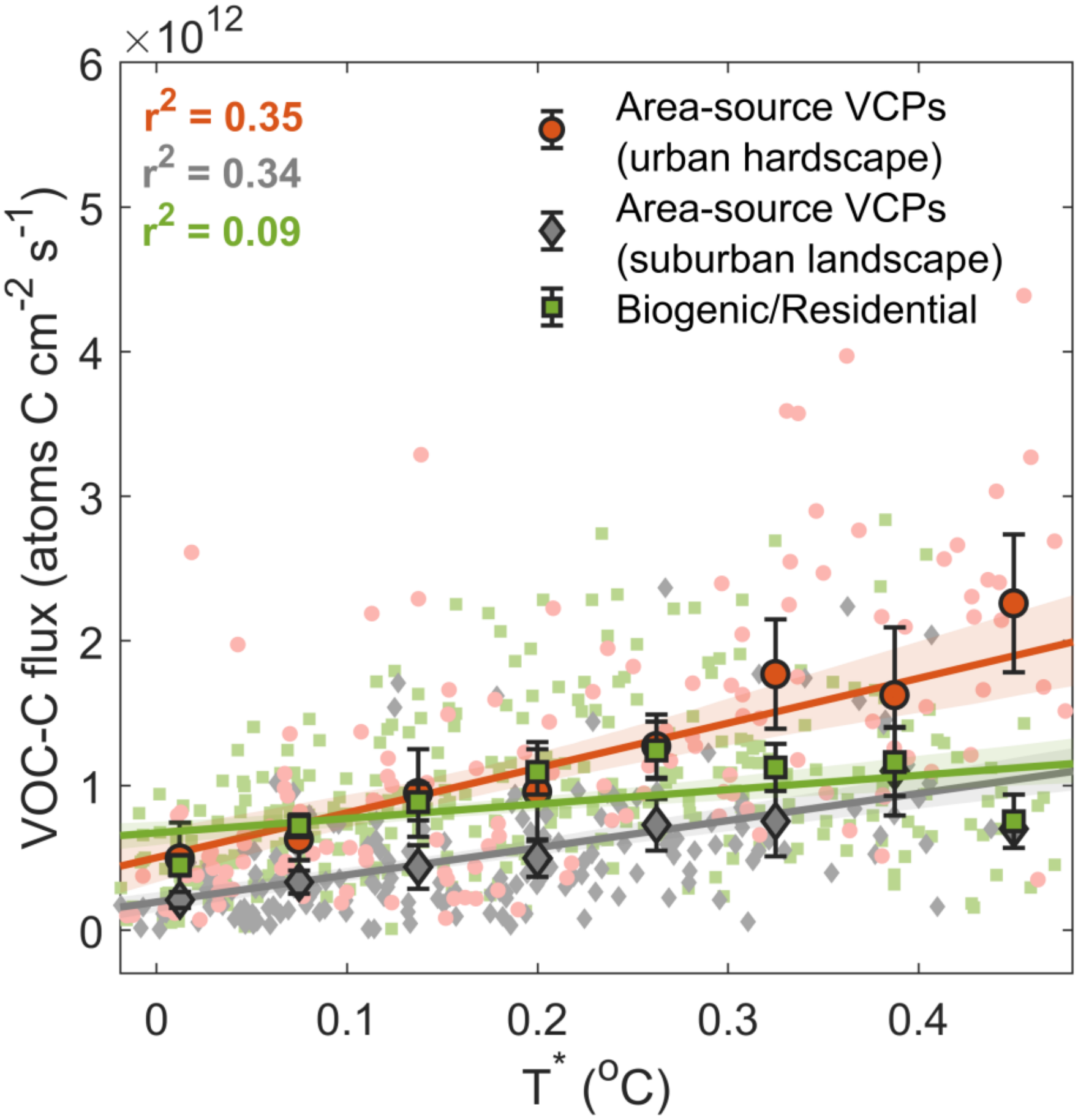

Figure S10: Area VCP ($F_{C,AVCP}$) and biogenic/residential ($F_{C,bio/res}$) VOC emissions plotted as a function of the dimensionless temperature scale (T_*, calculated as the ratio of sensible heat flux to u_*). Data are plotted separately for urban hardscapes (orange/red circles) and for suburban landscapes (grey triangles). Lines show the fitted linear relationship between fluxes and T_*, with the 95% bootstrapped confidence intervals plotted as the shaded area and the corresponding parameters listed inset. Binned data points include 95% confidence intervals around the mean.

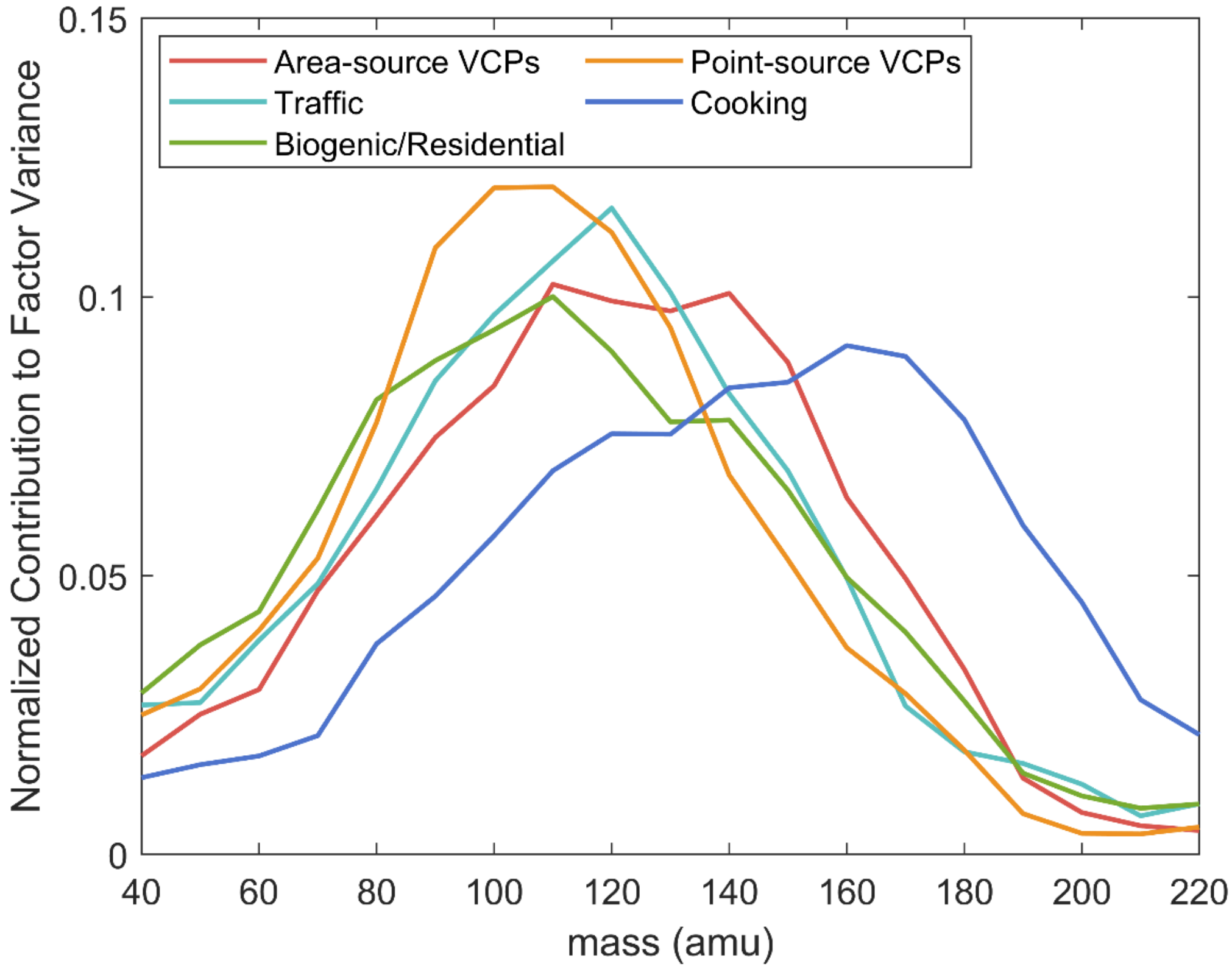

Figure S11: Normalized contribution to VOC flux factor variability by molecular weight during summer 2023.

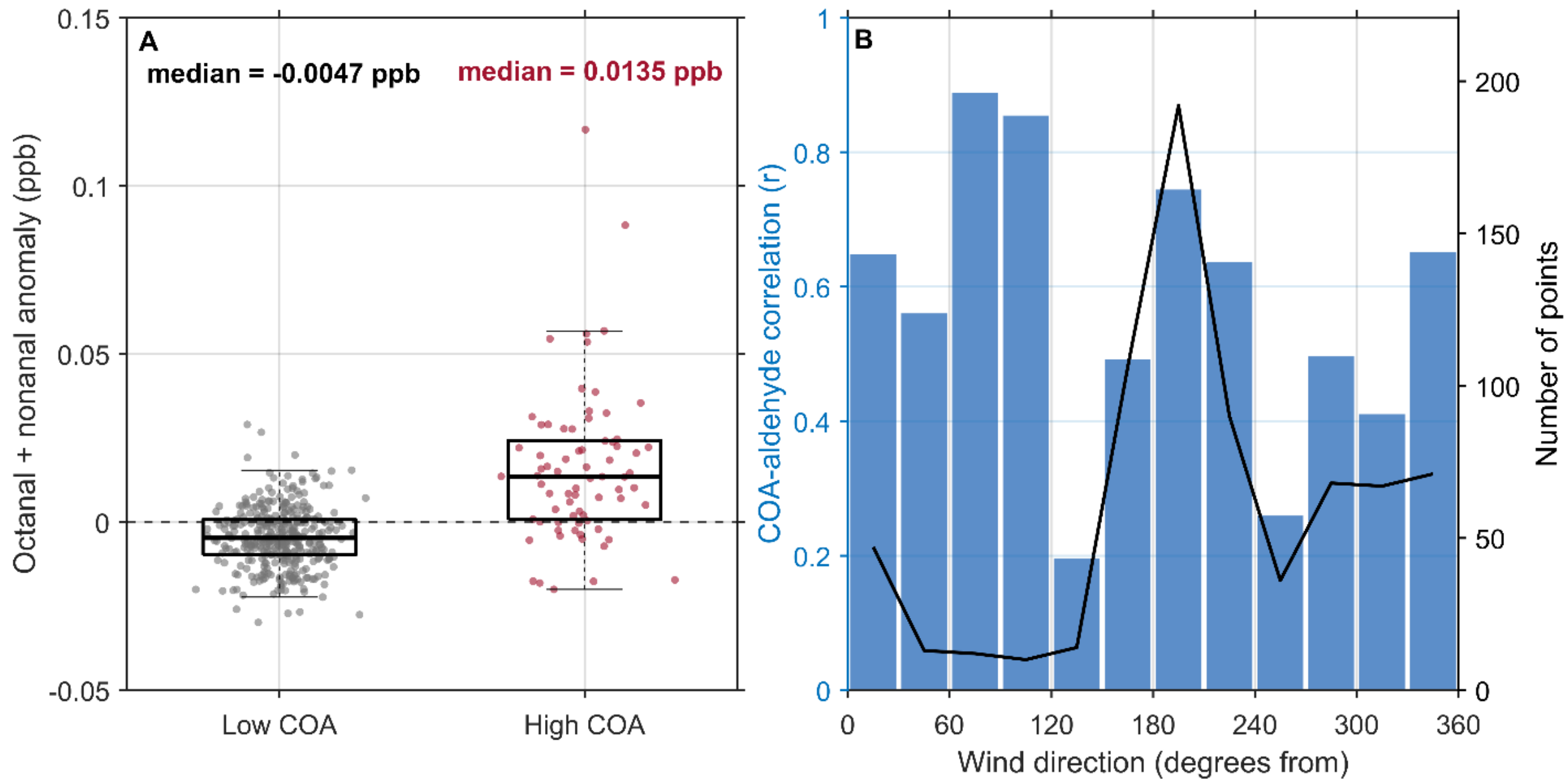

Figure S12: Relationship between cooking organic aerosol (COA) and aldehyde anomalies. (A) Octanal + nonanal anomalies during periods of low COA (≤ median anomaly) and high COA (top 10% of anomalies). (B) Correlation coefficient (r) between diurnally-detrended COA and octanal + nonanal anomalies as a function of wind direction are indicated with blue bars, while the number of observations in each wind sector is a black line.

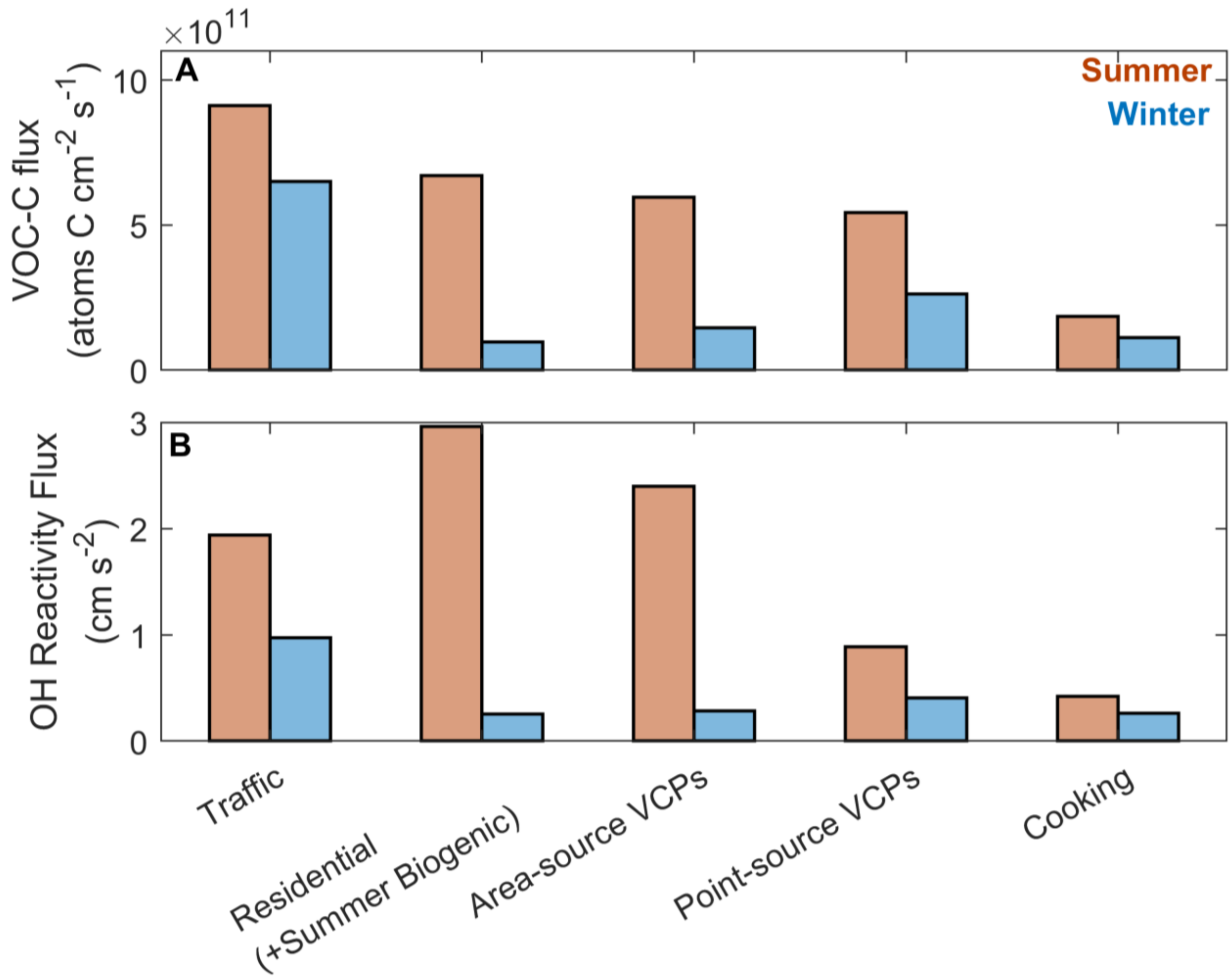

Figure S13: Contributions of traffic, residential (residential/biogenic in summer), area-source VCPs, point-source VCPs, and cooking to the total measured VOC-C fluxes (a) and OH reactivity fluxes (b) during summer (red bars) and winter (blue bars).

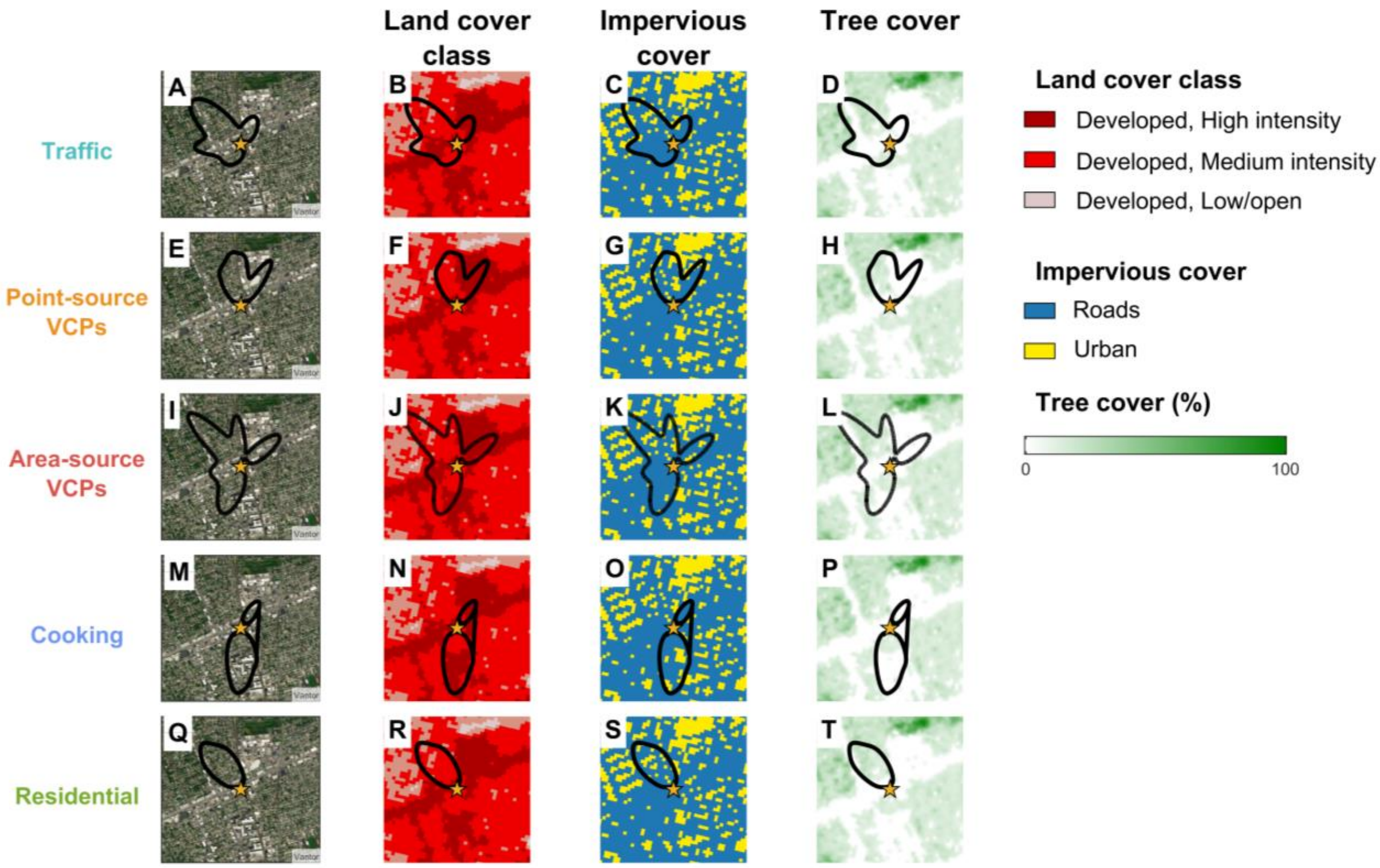

Figure S14: Cumulative 70% flux footprints during sampling intervals associated with the top 10th percentile for each NNMF source factor during winter. Footprints are overlaid on the Esri World Imagery basemap (*88*) (left column) and on National Land Cover Database 2023 products for land cover class, impervious cover, and tree cover (remaining columns) (*75*). Map image is the intellectual property of Esri and is used herein under license. Copyright © 2026 Esri and its licensors. All rights reserved.

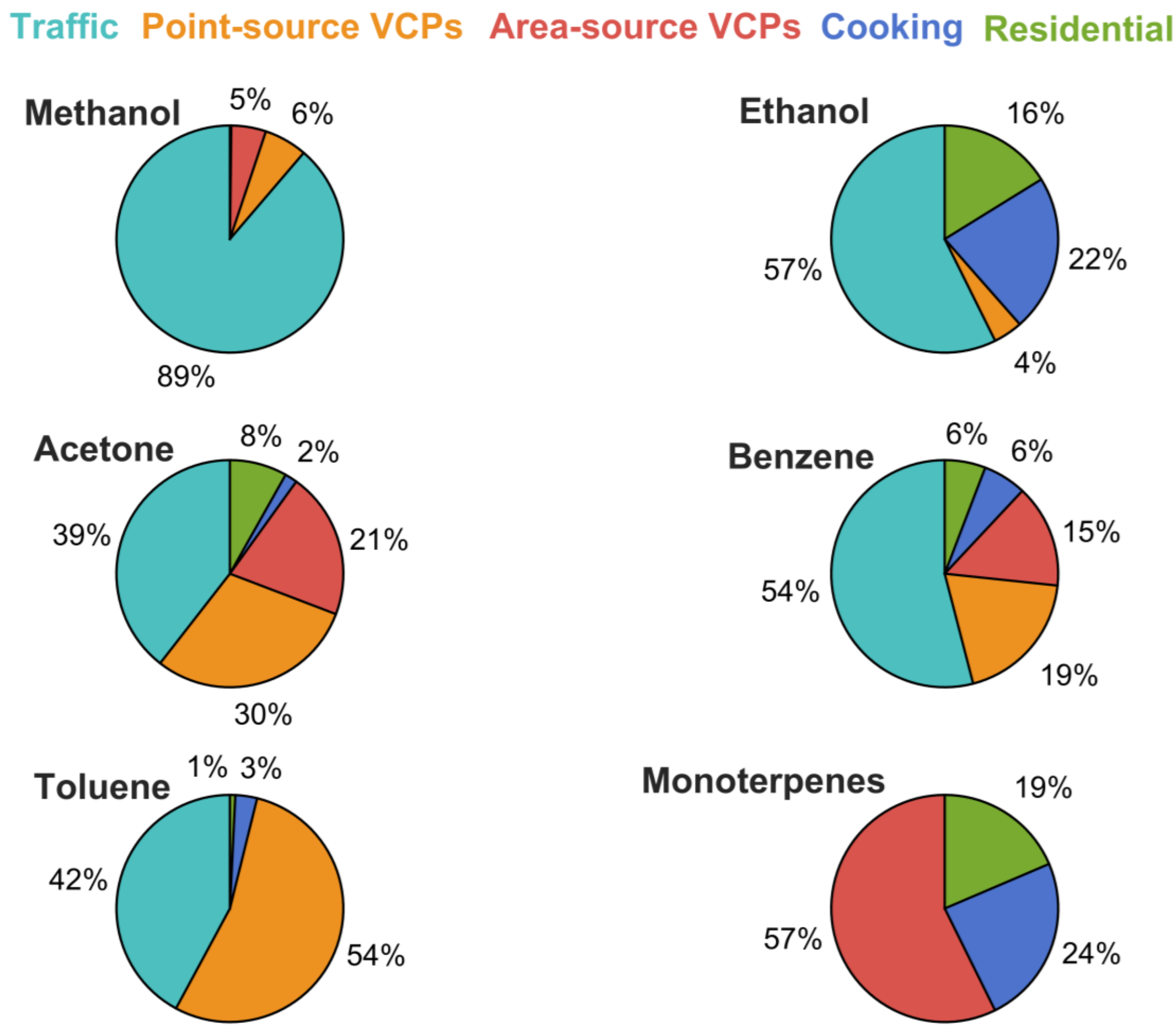

Figure S15: Mass attribution of select measured VOCs to the NNMF source factors for winter 2024.

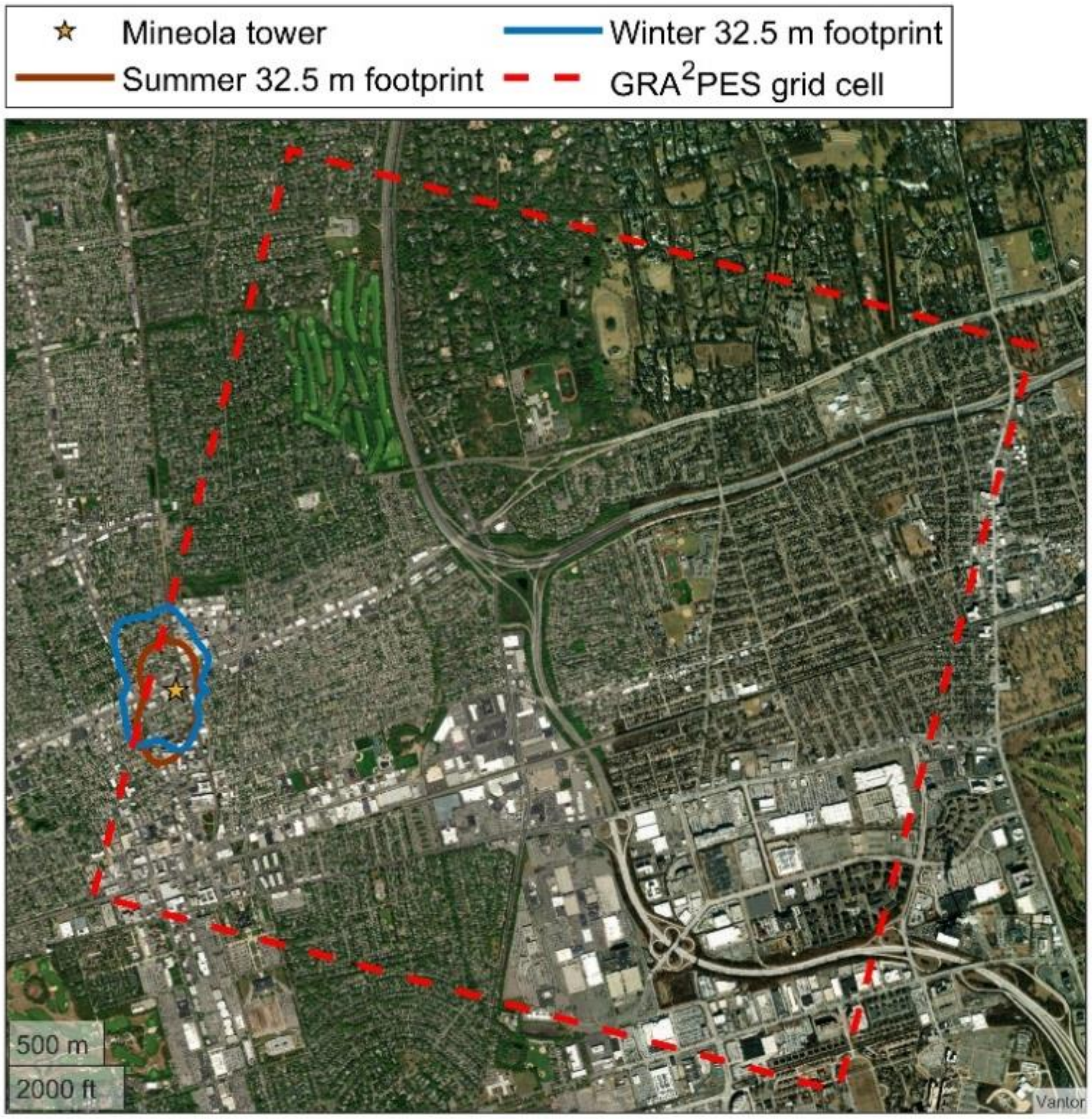

Figure S16: Mineola tower flux footprints and the GRA^{2}PES inventory grid cell. Mean 32.5 m flux footprints during summer (dark red) and winter (blue) are overlaid on the Esri World Imagery basemap (*88*). The red dashed polygon denotes the 4 km × 4 km GRA^{2}PES grid cell used for inventory comparisons, and the star indicates the tower location. Map image is the intellectual property of Esri and is used herein under license. Copyright © 2026 Esri and its licensors. All rights reserved.

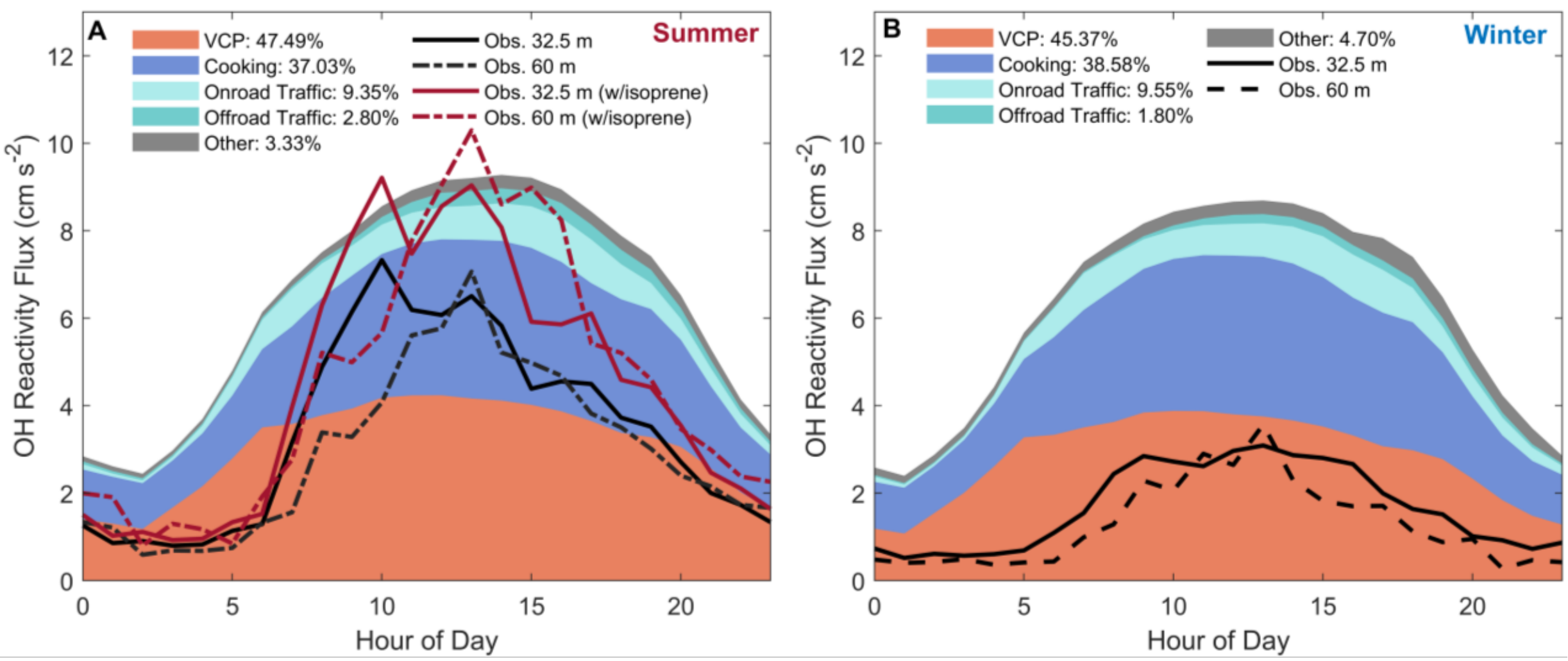

Figure S17: Diel OH reactivity flux profiles as predicted and observed during summer (A) and winter (B). In each case the GRA^{2}PES inventory estimates (stacked colors) are compared to fluxes measured at 32.5 m (solid lines) and 60 m (dashed lines) with biogenic/residential contributions removed. Measured results are shown separately with (red lines) and without (black lines) including anthropogenically-attributed isoprene.

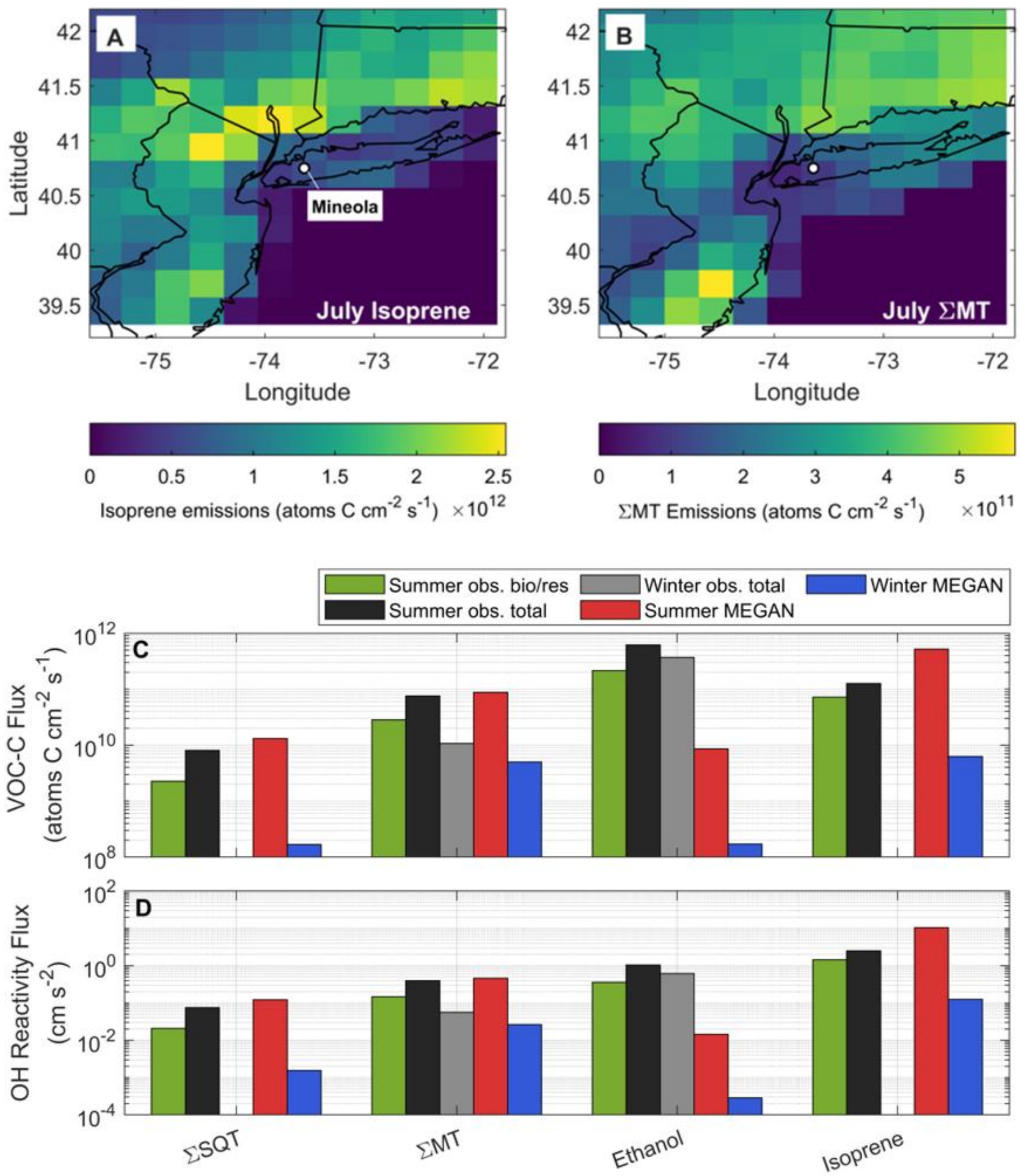

Figure S18: Comparison of observed and modeled biogenic VOC emissions. (A–B) July mean GEOS-Chem/MEGAN emissions of isoprene and total monoterpenes (ΣMT) with Mineola, NY indicated by the white circle. (C–D) Comparison of observed summer biogenic/residential fluxes (green), observed summer total fluxes (black), observed winter total fluxes (gray), and MEGAN-predicted summer (red) and winter (blue) emissions for key biogenic tracers. Fluxes are shown as VOC-C fluxes (C) and OH reactivity fluxes (D).

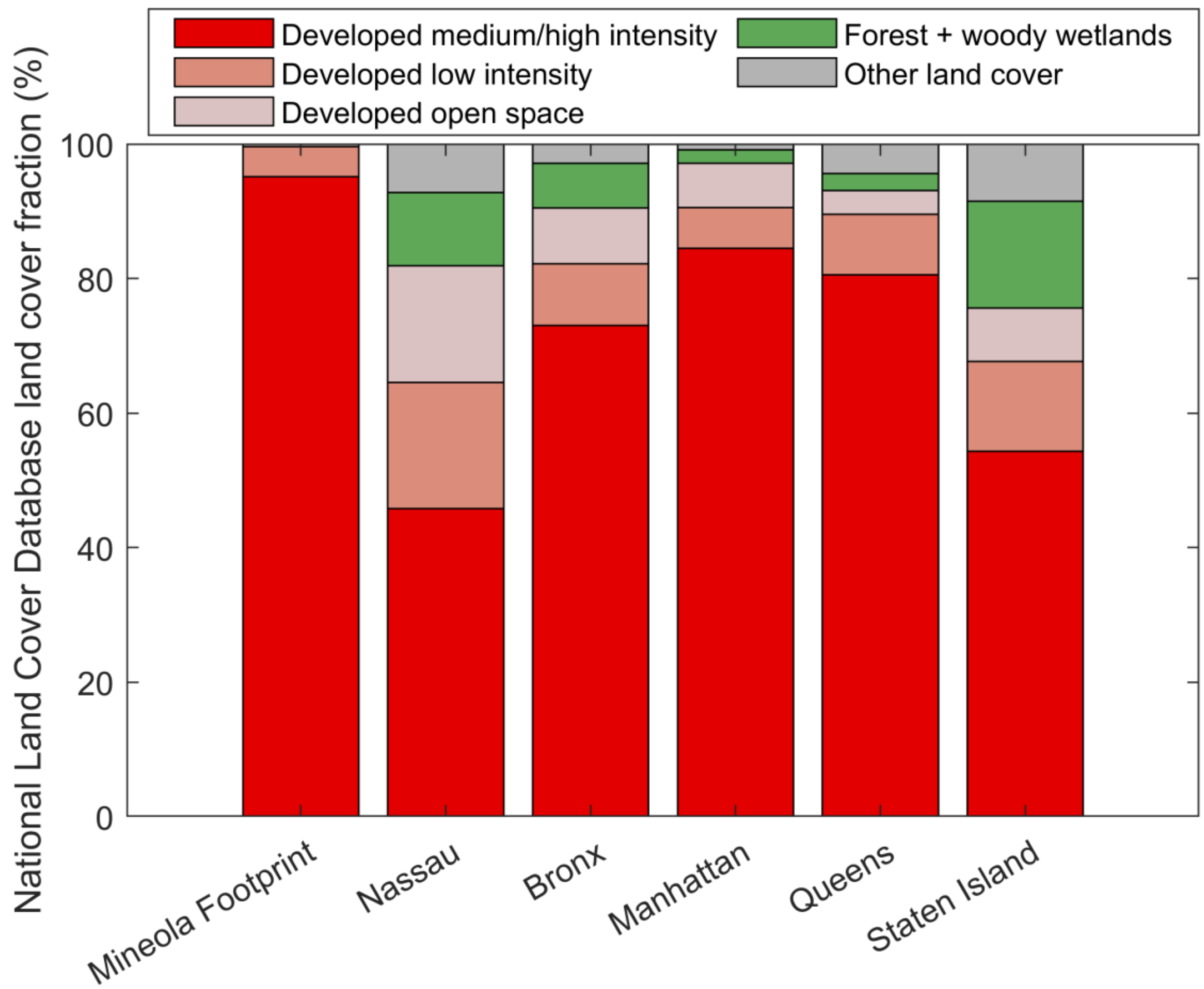

Figure S19: Land cover fractions within the Mineola flux footprint, surrounding New York City boroughs, and Nassau County, NY, which contains Mineola, from the 2023 National Land Cover Database (NLCD) (*75*).

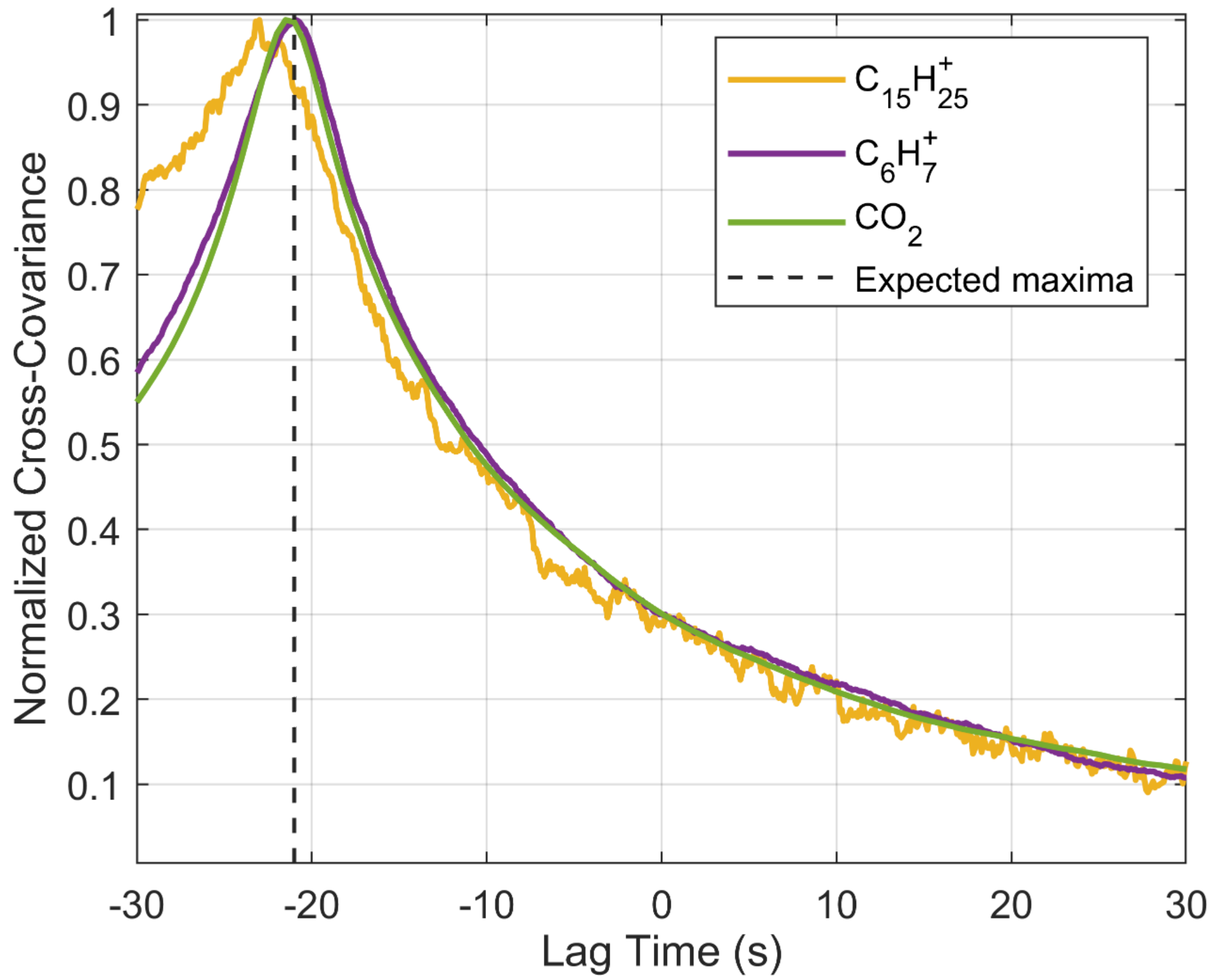

Figure S20: Normalized daytime-averaged cross-covariance for $C_{15}H_{25}^+$ (sesquiterpenes), $C_6H_7^+$ (benzene), and CO_2. The dashed line indicates the expected lag based on the calculated air residence time within the flux sampling inlet (21 s).

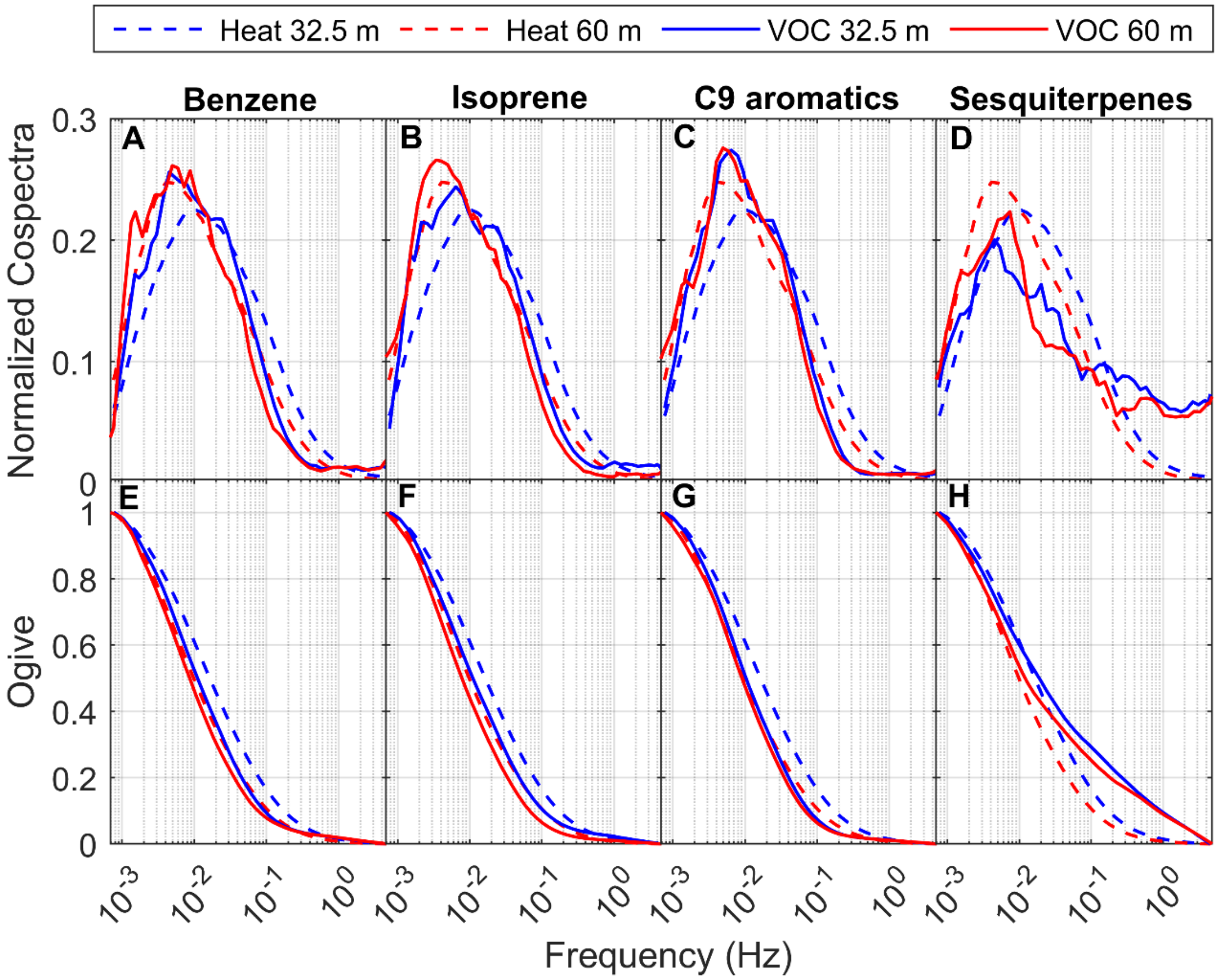

Figure S21: Frequency-normalized cospectra (A-D) and ogives (E-H) for select VOCs (solid lines) and for sensible heat (dashed lines) at 32.5 m (blue) and 60 m (red).

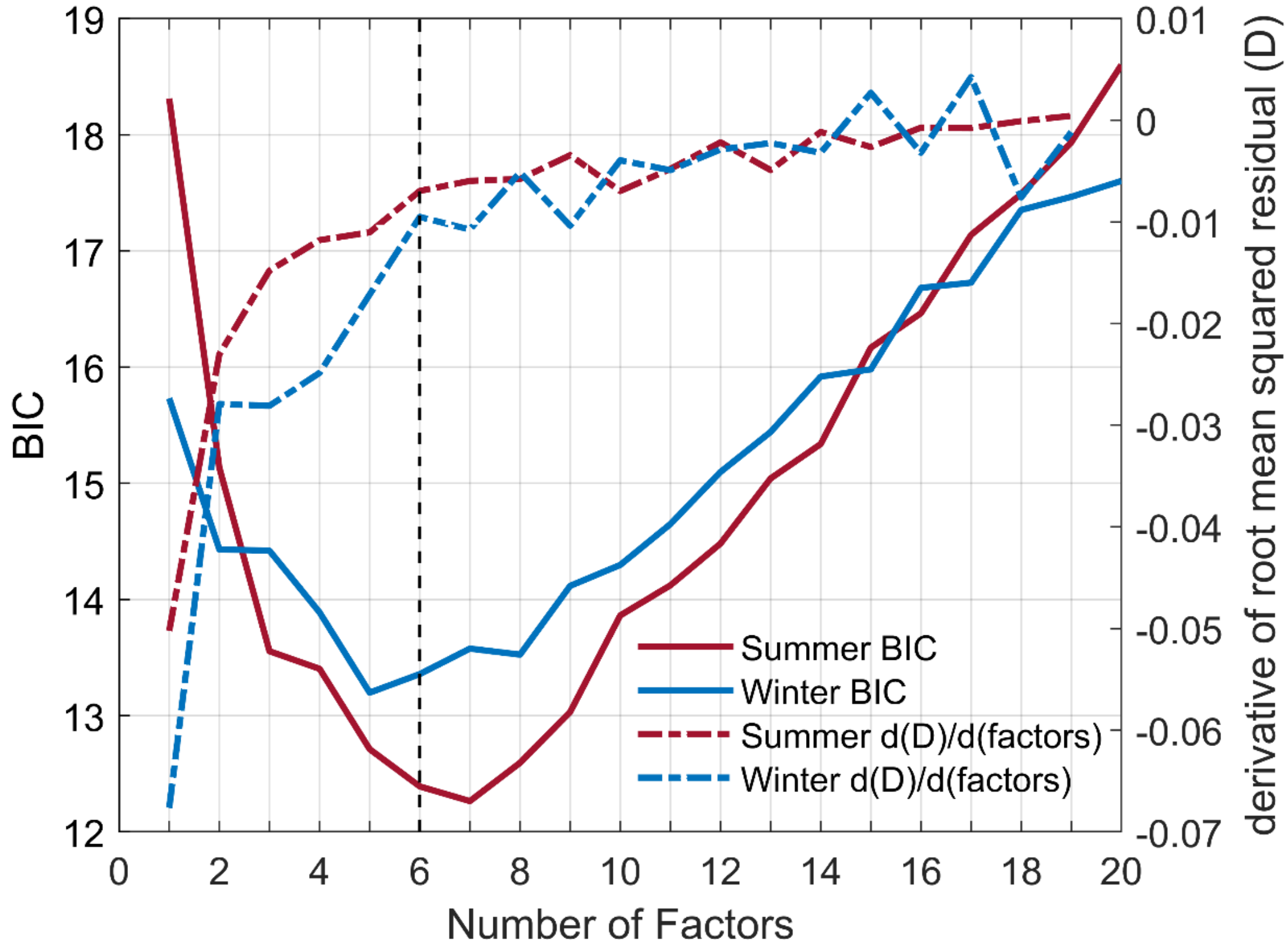

Figure S22: Bayesian Information Criterion (BIC) vs. number of NNMF factors (solid lines), and the derivative of the root mean squared residual between $\overleftrightarrow{A}$ and $\overleftrightarrow{W} \times \overleftrightarrow{H}$ vs. number of NNMF factors (dashed lines). The optimal number of factors is indicated by the black dashed line.

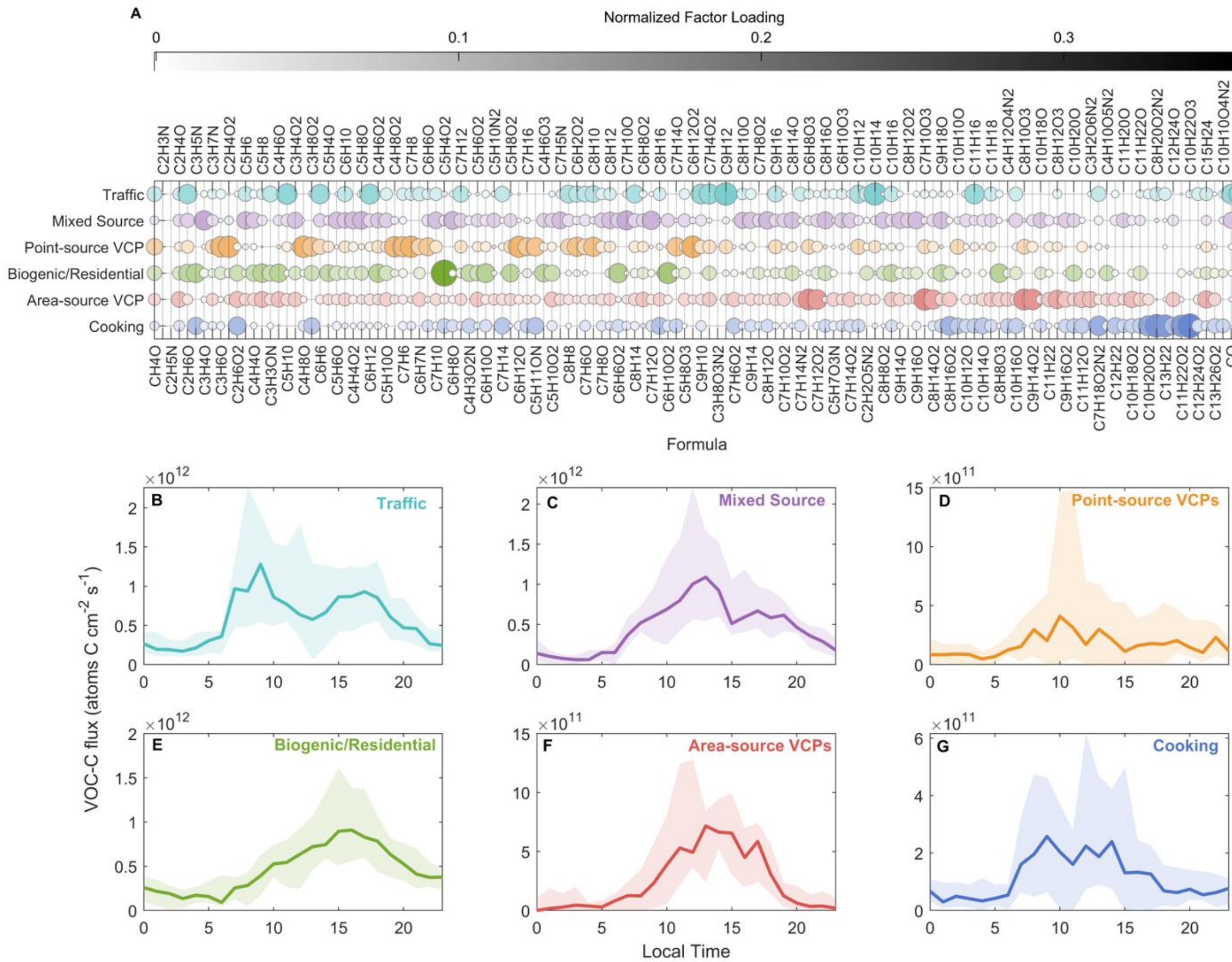

Figure S23: Sectoral contributions to the measured fluxes as determined by non-negative matrix factorization (NNMF) for summer 2023 using six factors instead of five. (A) Contribution of each species to the total variance for each factor. Circle radius and shading are proportional to the NNMF factor loading for each species within each factor. (B-G) Diel flux profiles for each factor (median and interquartile range).

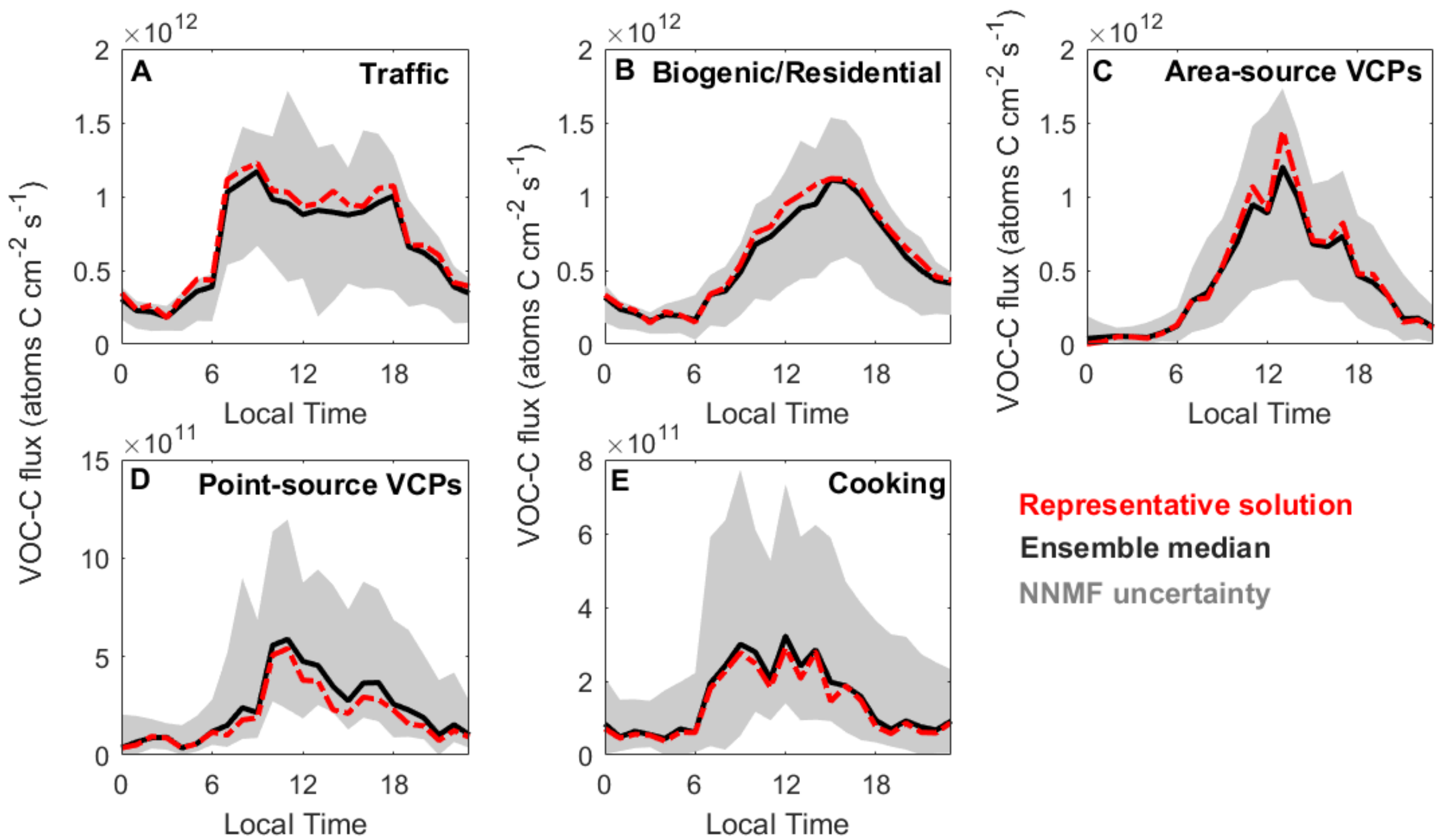

Figure S24: Comparison of the representative summer NNMF solution (red dashed line) with the median across 1000 NNMF realizations (black line). Shading indicates the central 5th-95th percentile for the combined ensemble of 500 seed initializations and 500 bootstrap resamples.

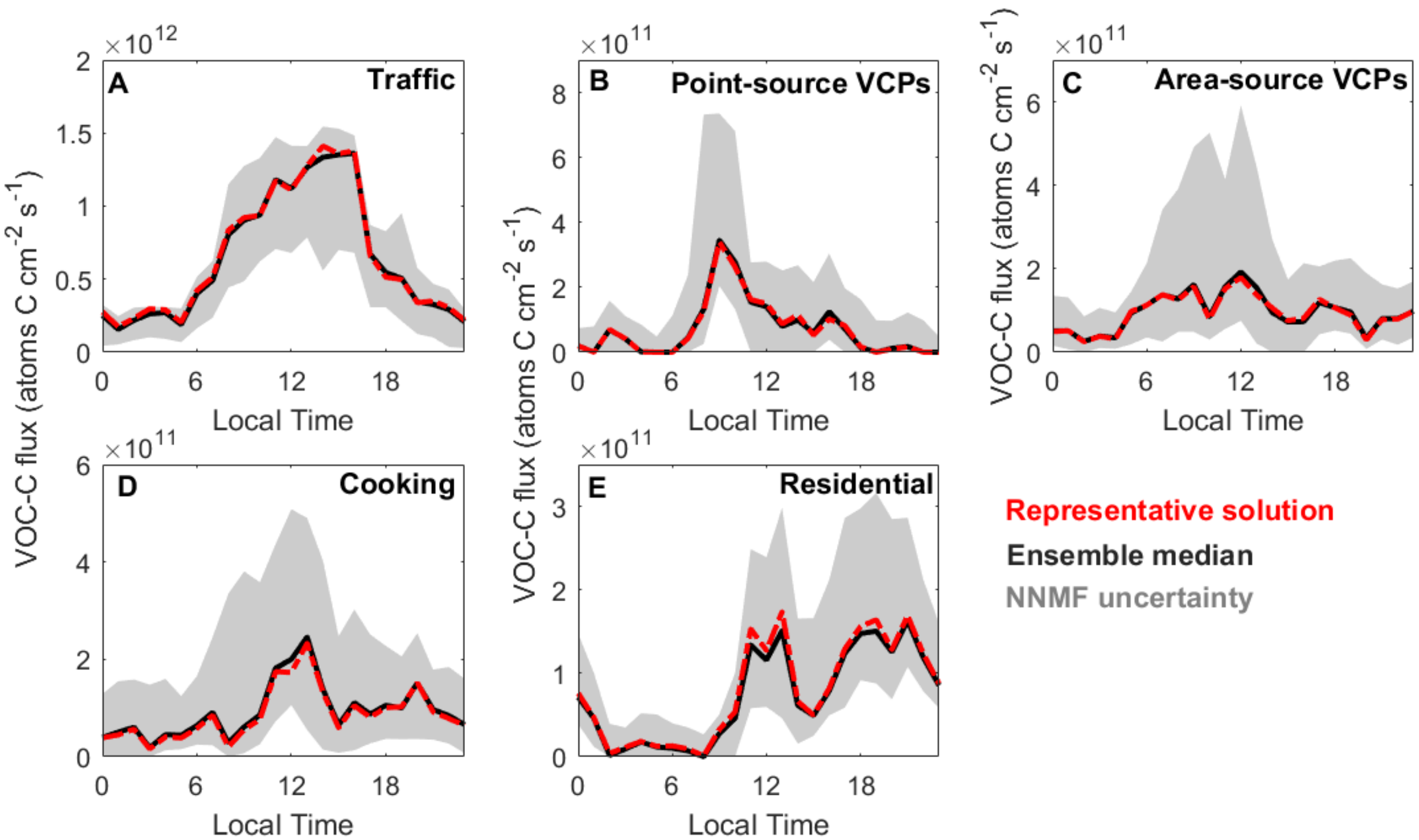

Figure S25: Comparison of the representative winter NNMF solution (red dashed line) with the median across 1000 NNMF realizations (black line). Shading indicates the central 5th-95th percentile for the combined ensemble of 500 seed initializations and 500 bootstrap resamples.

Table S1: List of GRA^{2}PES VOCs used for this analysis and detectable by PTRMS. Also included are the chemical formulae as detected by PTRMS. "Mean flux does not satisfy detection criterion" indicates that the campaign-mean flux for that compound did not exceed the corresponding campaign-mean flux uncertainty.

Category	Inventory Species	Detected Chemical Formula
Anthropogenic Isoprene	Isoprene	$C_5H_9^+$
Anthropogenic Terpenes (VCPs)	Limonene	$C_{10}H_{17}^+$
Acetaldehyde	Acetaldehyde	$C_2H_5O^+$
>C2 aldehydes	Butanal	$C_4H_9O^+$
Benzaldehyde	Benzaldehyde	$C_7H_7O^+$
Acetone	Acetone	$C_3H_7O^+$
Methylethyl ketone	Methyl ethyl ketone	$C_4H_9O^+$
PRD2 SAPRAC species (aromatic ketones)	Acetophenone	Mean flux does not satisfy detection criterion
Methanol	Methanol	CH_5O^+
Glyoxal	Glyoxal	Mean flux does not satisfy detection criterion
Methylglyoxal	Methylglyoxal	Mean flux does not satisfy detection criterion
Biacetyl	2,3-butanedione	$C_4H_7O_2^+$
Phenols	Phenol	$C_6H_7O^+$
Cresols	o,m,p-cresol	$C_7H_9O^+$
Methacrolein	Methacrolein	$C_4H_7O^+$
Methylvinyl ketone	Methyl vinyl ketone	$C_4H_7O^+$
IPRD SAPRAC species (>C4 unsaturated aldehydes)	Pentenal	Mean flux does not satisfy detection criterion
Formic Acid	Formic acid	Mean flux does not satisfy detection criterion
Acetic Acid	Acetic acid	$C_2H_5O_2^+$
>C2 Acids	Propanoic acid	$C_5H_9O^+$
Xylenols	Xylenol	$C_8H_{11}O^+$
Catechol	Catechol	$C_6H_7O_2^+$
Benzene	Benzene	$C_6H_7^+$
Toluene	Toluene	$C_7H_9^+$
m-Xylene	m-Xylene	$C_8H_{11}^+$
o-Xylene	o-Xylene	$C_8H_{11}^+$
p-Xylene	p-Xylene	$C_8H_{11}^+$
Dienes	3-methyl-1,3-pentadiene<br>1,4-hexadiene<br>1,2-pentadiene	Mean flux does not satisfy detection criterion
Styrenes	Styrene	$C_8H_9^+$
Ethanol	Ethanol	$C_2H_7O^+$
Ethylene Glycol	Ethylene glycol	$C_2H_7O_2^+$

Unidentified Unknown VOC	Mean carbon number of inventory VOCs	Mean flux does not satisfy detection criterion
Isopropyl Alcohol (OVCP)	Isopropyl alcohol	Mean flux does not satisfy detection criterion
Propylene Glycol (OVCP)	Propylene glycol	$C_3H_9O_2^+$
Glycerol (OVCP)	Glycerol	Mean flux does not satisfy detection criterion
D4-Siloxane	D4-Siloxane	Mean flux does not satisfy detection criterion
D5-Siloxane	D5-Siloxane	Mean flux does not satisfy detection criterion
Other Siloxane	Avg of D4/D5-Siloxane	Mean flux does not satisfy detection criterion
PCBTF	*p*-chlorobenzotrifluoride	Mean flux does not satisfy detection criterion
PDCBZ	*p*-dichlorobenzene	Mean flux does not satisfy detection criterion
Propanal	Propanal	Mean flux does not satisfy detection criterion
Butanal	Butanal	$C_4H_9O^+$
Pentanal	Pentanal	$C_5H_{11}O^+$
Hexanal	Hexanal	$C_6H_{13}O^+$
Heptanal	Heptanal	$C_7H_{15}O^+$
Octanal	Octanal	$C_8H_{17}O^+$
Nonanal	Nonanal	$C_9H_{19}O^+$
Unsaturated Aldehydes	Crotonaldehyde	Mean flux does not satisfy detection criterion

Table S2: Species and concentrations contained in VOC cylinders used for in-field PTRMS calibration. All standards have a concentration uncertainty of ±5%.

**Species name**	**Formula**	**Concentration (ppb)**
Acetaldehyde	CH_3CHO	1028
Methanol	CH_3OH	4969
Acetonitrile	CH_3CN	1008
Acetone	CH_3COCH_3	2439
Isoprene	C_5H_8	2477
Methyl ethyl ketone	C_4H_8O	1049
Hydroxyacetone	$C_3H_6O_2$	1280
Benzene	C_6H_6	1047
Toluene	C_7H_8	1042
m-Xylene	C_8H_{10}	1018
1,3,5-Trimethylbenzene	C_9H_{12}	950
1,2,4,5-Tetramethylbenzene	$C_{10}H_{14}$	990
Propene	C_3H_6	1001
Furan	C_4H_4O	1037

DMS	C_2H_6S	1065
Methacrolein	C_4H_6O	1047
2-Methyl-3-Buten-2-ol	$C_5H_{10}O$	1040
3-Hexanone	$C_6H_{12}O$	1003
β-pinene	$C_{10}H_{16}$	1009
Propyne	C_3H_4	970
1-Butene	C_4H_8	982
Ethanol	C_2H_6O	1032
Methyl Vinyl Ketone	C_4H_6O	979
3-Methyl Furan	C_5H_6O	973
3-Pentanone	$C_5H_{10}O$	1025
Ethyl Benzene	C_8H_{10}	991
α-pinene	$C_{10}H_{16}$	997
Nitromethane	CH_3NO_2	975
2-Vinylfuran	C_6H_6O	362
1-Chloro-4-(trifluoromethyl)benzene (PCBTF)	$C_7H_4ClF_3$	1075
1,4-Dichlorobenzene	$C_6H_4Cl_2$	1056
Octamethylcyclotetrasiloxane (D4)	$[(CH_3)_2SiO]_4$	916
Limonene	$C_{10}H_{16}$	1031
Decamethylcyclopentasiloxane (D5)	$[(CH_3)_2SiO]_5$	927

REFERENCES

1. IPCC, "Climate Change 2014: Synthesis Report. Contribution of Working Groups I, II and III to the Fifth Assessment Report of the Intergovernmental Panel on Climate Change" (IPCC, 2014).

2. U. B. Nurmatov, N. Tagieva, S. Semple, G. Devereux, A. Sheikh, Volatile organic compounds and risk of asthma and allergy: A systematic review and meta-analysis of observational and interventional studies. *Prim. Care Respir. J.* **22**, S9–S15 (2013).

3. G. St. Helen, P. Jacob, M. Peng, D. A. Dempsey, S. K. Hammond, N. L. Benowitz, Intake of toxic and carcinogenic volatile organic compounds from secondhand smoke in motor vehicles. *Cancer Epidemiol. Biomarkers Prev.* **23**, 2774–2782 (2014).

4. M. Jerrett, R. T. Burnett, C. A. Pope, K. Ito, G. Thurston, D. Krewski, Y. Shi, E. Calle, M. Thun, Long-term ozone exposure and mortality. *N. Engl. J. Med.* **360**, 1085–1095 (2009).

5. Q. Di, Y. Wang, A. Zanobetti, Y. Wang, P. Koutrakis, C. Choirat, F. Dominici, J. D. Schwartz, Air pollution and mortality in the medicare population. *N. Engl. J. Med.* **376**, 2513–2522 (2017).

6. D. R. Gentner, S. H. Jathar, T. D. Gordon, R. Bahreini, D. A. Day, I. El Haddad, P. L. Hayes, S. M. Pieber, S. M. Platt, J. De Gouw, A. H. Goldstein, R. A. Harley, J. L. Jimenez, A. S. H. Prévôt, A. L. Robinson, Review of urban secondary organic aerosol formation from gasoline and diesel motor vehicle emissions. *Environ. Sci. Technol.* **51**, 1074–1093 (2017).

7. S. W. Kim, S. A. McKeen, G. J. Frost, S. H. Lee, M. Trainer, A. Richter, W. M. Angevine, E. Atlas, L. Bianco, K. F. Boersma, J. Brioude, J. P. Burrows, J. De Gouw, A. Fried, J. Gleason, A. Hilboll, J. Mellqvist, J. Peischl, D. Richter, C. Rivera, T. Ryerson, S. Te Lintel Hekkert, J. Walega, C. Warneke, P. Weibring, E. Williams, Evaluations of NO_x and highly reactive VOC emission inventories in Texas and their implications for ozone plume simulations during the Texas Air Quality Study 2006. *Atmos. Chem. Phys.* **11**, 11361–11386 (2011).

8. C. Warneke, J. A. De Gouw, J. S. Holloway, J. Peischl, T. B. Ryerson, E. Atlas, D. Blake, M. Trainer, D. D. Parrish, Multiyear trends in volatile organic compounds in Los Angeles,

California: Five decades of decreasing emissions. *J. Geophys. Res. Atmos.* **117**, D00V17 (2012).

9. B. C. McDonald, D. R. Gentner, A. H. Goldstein, R. A. Harley, Long-term trends in motor vehicle emissions in U.S. urban areas. *Environ. Sci. Technol.* **47**, 10022–10031 (2013).

10. B. C. McDonald, J. A. De Gouw, J. B. Gilman, S. H. Jathar, A. Akherati, C. D. Cappa, J. L. Jimenez, J. Lee-Taylor, P. L. Hayes, S. A. McKeen, Y. Y. Cui, S. W. Kim, D. R. Gentner, G. Isaacman-VanWertz, A. H. Goldstein, R. A. Harley, G. J. Frost, J. M. Roberts, T. B. Ryerson, M. Trainer, Volatile chemical products emerging as largest petrochemical source of urban organic emissions. *Science* **359**, 760–764 (2018).

11. M. M. Coggon, G. I. Gkatzelis, B. C. McDonald, J. B. Gilman, R. H. Schwantes, N. Abuhassan, K. C. Aikin, M. F. Arendd, T. A. Berkoff, S. S. Brown, T. L. Campos, R. R. Dickerson, G. Gronoff, J. F. Hurley, G. Isaacman-Vanwertz, A. R. Koss, M. Li, S. A. McKeen, F. Moshary, J. Peischl, V. Pospisilova, X. Ren, A. Wilson, Y. Wu, M. Trainer, C. Warneke, Volatile chemical product emissions enhance ozone and modulate urban chemistry. *Proc. Natl. Acad. Sci. U.S.A.* **118**, e2026653118 (2021).

12. G. I. Gkatzelis, M. M. Coggon, B. C. McDonald, J. Peischl, J. B. Gilman, K. C. Aikin, M. A. Robinson, F. Canonaco, A. S. H. Prevot, M. Trainer, C. Warneke, Observations confirm that volatile chemical products are a major source of petrochemical emissions in U.S. cities. *Environ. Sci. Technol.* **55**, 4332–4343 (2021).

13. G. I. Gkatzelis, M. M. Coggon, B. C. McDonald, J. Peischl, K. C. Aikin, J. B. Gilman, M. Trainer, C. Warneke, Identifying volatile chemical product tracer compounds in U.S. cities. *Environ. Sci. Technol.* **55**, 188–199 (2021).

14. M. M. Coggon, B. C. McDonald, A. Vlasenko, P. R. Veres, F. Bernard, A. R. Koss, B. Yuan, J. B. Gilman, J. Peischl, K. C. Aikin, J. Durant, C. Warneke, S. M. Li, J. A. De Gouw, Diurnal variability and emission pattern of decamethylcyclopentasiloxane (D5) from the application of personal care products in two North American cities. *Environ. Sci. Technol.* **52**, 5610–5618 (2018).

15. D. Wei, C. Cao, A. Karambelas, J. Mak, A. Reinmann, R. Commane, High-resolution modeling of summertime biogenic isoprene emissions in New York City. *Environ. Sci. Technol.* **58**, 13783–13794 (2024).

16. M. M. Coggon, C. E. Stockwell, L. Xu, J. Peischl, J. B. Gilman, A. Lamplugh, H. J. Bowman, K. Aikin, C. Harkins, Q. Zhu, R. H. Schwantes, J. He, M. Li, K. Seltzer, B. Mcdonald, C. Warneke, Contribution of cooking emissions to the urban volatile organic compounds in Las Vegas, NV. *Atmos. Chem. Phys.* **24**, 4289–4304 (2024).

17. P. Harb, N. Locoge, F. Thevenet, Emissions and treatment of VOCs emitted from wood-based construction materials: Impact on indoor air quality. *Chem. Eng. J.* **354**, 641–652 (2018).

18. Y. S. Son, B. A. Lim, H. J. Park, J. C. Kim, Characteristics of volatile organic compounds (VOCs) emitted from building materials to improve indoor air quality: Focused on natural VOCs. *Air Qual. Atmos. Health* **6**, 737–746 (2013).

19. B. Molinier, C. Arata, D. M. Lunderberg, B. C. Singer, W. W. Nazaroff, A. H. Goldstein, Volatile organic compound composition and emissions in a residential attic. *ACS ES T Air* **1**, 559–569 (2024).

20. J. M. Mattila, C. Arata, A. Abeleira, Y. Zhou, C. Wang, E. F. Katz, A. H. Goldstein, J. P. D. Abbatt, P. F. DeCarlo, M. E. Vance, D. K. Farmer, Contrasting chemical complexity and the reactive organic carbon budget of indoor and outdoor air. *Environ. Sci. Technol.* **56**, 109–118 (2022).

21. D. B. Millet, H. D. Alwe, X. Chen, M. J. Deventer, T. J. Griffis, R. Holzinger, S. B. Bertman, P. S. Rickly, P. S. Stevens, T. Léonardis, N. Locoge, S. Dusanter, G. S. Tyndall, S. L. Alvarez, M. H. Erickson, J. H. Flynn, Bidirectional ecosystem-atmosphere fluxes of volatile organic compounds across the mass spectrum: How many matter? *ACS Earth Space Chem.* **2**, 764–777 (2018).

22. M. P. Vermeuel, D. B. Millet, D. K. Farmer, M. A. Pothier, M. F. Link, M. Riches, S. Williams, L. A. Garofalo, Closing the reactive carbon flux budget: Observations from dual

mass spectrometers over a coniferous forest. *J. Geophys. Res. Atmos.* **128**, e2023JD038753 (2023).

23. M. P. Vermeuel, G. A. Novak, D. B. Kilgour, M. S. Claflin, B. M. Lerner, A. M. Trowbridge, J. Thom, P. A. Cleary, A. R. Desai, T. H. Bertram, Observations of biogenic volatile organic compounds over a mixed temperate forest during the summer to autumn transition. *Atmos. Chem. Phys.* **23**, 4123–4148 (2023).

24. L. Kaser, T. Karl, A. Guenther, M. Graus, R. Schnitzhofer, A. Turnipseed, L. Fischer, P. Harley, M. Madronich, D. Gochis, F. N. Keutsch, A. Hansel, Undisturbed and disturbed above canopy ponderosa pine emissions: PTR-TOF-MS measurements and MEGAN 2.1 model results. *Atmos. Chem. Phys.* **13**, 11935–11947 (2013).

25. C. Sarkar, A. B. Guenther, J. H. Park, R. Seco, E. Alves, S. Batalha, R. Santana, S. Kim, J. Smith, J. Tóta, O. Vega, PTR-TOF-MS eddy covariance measurements of isoprene and monoterpene fluxes from an eastern Amazonian rainforest. *Atmos. Chem. Phys.* **20**, 7179–7191 (2020).

26. A. B. Guenther, X. Jiang, C. L. Heald, T. Sakulyanontvittaya, T. Duhl, L. K. Emmons, X. Wang, The Model of Emissions of Gases and Aerosols from Nature version 2.1 (MEGAN2.1): An extended and updated framework for modeling biogenic emissions. *Geosci. Model Dev.* **5**, 1471–1492 (2012).

27. E. Velasco, S. Pressley, R. Grivicke, E. Allwine, T. Coons, W. Foster, B. T. Jobson, H. Westberg, R. Ramos, F. Hernández, L. T. Molina, B. Lamb, Eddy covariance flux measurements of pollutant gases in urban Mexico City. *Atmos. Chem. Phys.* **9**, 7325–7342 (2009).

28. E. Y. Pfannerstill, C. Arata, Q. Zhu, B. C. Schulze, R. Ward, R. Woods, C. Harkins, R. H. Schwantes, J. H. Seinfeld, A. Bucholtz, R. C. Cohen, A. H. Goldstein, Temperature-dependent emissions dominate aerosol and ozone formation in Los Angeles. *Science* **384**, 1324–1329 (2024).

29. M. Qin, Y. She, M. Wang, H. Wang, Y. Chang, Z. Tan, J. An, J. Huang, Z. Yuan, J. Lu, Q. Wang, C. Liu, Z. Liu, X. Xie, J. Li, H. Liao, H. O. T. Pye, C. Huang, S. Guo, M. Hu, Y. Zhang, D. J. Jacob, J. Hu, Increased urban ozone in heatwaves due to temperature-induced emissions of anthropogenic volatile organic compounds. *Nat. Geosci.* **18**, 50–56 (2025).

30. J. I. Rubin, A. J. Kean, R. A. Harley, D. B. Millet, A. H. Goldstein, Temperature dependence of volatile organic compound evaporative emissions from motor vehicle. *J. Geophys. Res. Atmos.* **111**, D03305 (2006).

31. K. Na, K. C. Moon, P. K. Yong, Source contribution to aromatic VOC concentration and ozone formation potential in the atmosphere of Seoul. *Atmos. Environ.* **39**, 5517–5524 (2005).

32. T. Karl, E. Apell, A. Hodzic, D. D. Riemer, D. R. Blake, C. Wiedinmyer, Emissions of volatile organic compounds inferred from airborne flux measurements over a megacity. *Atmos. Chem. Phys.* **9**, 271–285 (2009).

33. J. Lasne, A. Lostier, M. N. Romanias, S. Vassaux, D. Lesueur, V. Gaudion, M. Jamar, R. G. Derwent, S. Dusanter, T. Salameh, VOC emissions by fresh and old asphalt pavements at service temperatures: Impacts on urban air quality. *Environ. Sci. Atmos.* **3**, 1601–1619 (2023).

34. W. Wu, T. M. Fu, S. R. Arnold, D. V. Spracklen, A. Zhang, W. Tao, X. Wang, Y. Hou, J. Mo, J. Chen, Y. Li, X. Feng, H. Lin, Z. Huang, J. Zheng, H. Shen, L. Zhu, C. Wang, J. Ye, X. Yang, Temperature-dependent evaporative anthropogenic VOC emissions significantly exacerbate regional ozone pollution. *Environ. Sci. Technol.* **58**, 5430–5441 (2024).

35. T. Karl, M. Striednig, M. Graus, A. Hammerle, G. Wohlfahrt, Urban flux measurements reveal a large pool of oxygenated volatile organic compound emissions. *Proc. Natl. Acad. Sci. U.S.A.* **115**, 1186–1191 (2018).

36. E. Y. Pfannerstill, C. Arata, Q. Zhu, B. C. Schulze, R. Woods, C. Harkins, R. H. Schwantes, B. C. McDonald, J. H. Seinfeld, A. Bucholtz, R. C. Cohen, A. H. Goldstein, Comparison between spatially resolved airborne flux measurements and emission inventories of volatile organic compounds in Los Angeles. *Environ. Sci. Technol.* **57**, 15533–15545 (2023).

37. C. Warneke, S. A. McKeen, J. A. de Gouw, P. D. Goldan, W. C. Kuster, J. S. Holloway, E. J. Williams, B. M. Lerner, D. D. Parrish, M. Trainer, F. C. Fehsenfeld, S. Kato, E. L. Atlas, A. Baker, D. R. Blake, Determination of urban volatile organic compound emission ratios and comparison with an emissions database. *J. Geophys. Res. Atmos.* **112**, D10S47 (2007).

38. A. Borbon, J. B. Gilman, W. C. Kuster, N. Grand, S. Chevaillier, A. Colomb, C. Dolgorouky, V. Gros, M. Lopez, R. Sarda-Esteve, J. Holloway, J. Stutz, H. Petetin, S. McKeen, M. Beekmann, C. Warneke, D. D. Parrish, J. A. De Gouw, Emission ratios of anthropogenic volatile organic compounds in northern mid-latitude megacities: Observations versus emission inventories in Los Angeles and Paris. *J. Geophys. Res. Atmos.* **118**, 2041–2057 (2013).

39. K. M. Seltzer, E. Pennington, V. Rao, B. N. Murphy, M. Strum, K. K. Isaacs, H. O. T. Pye, Reactive organic carbon emissions from volatile chemical products. *Atmos. Chem. Phys.* **21**, 5079–5100 (2021).

40. W. Zhao, P. K. Hopke, T. Karl, Source identification of volatile organic compounds in Houston, Texas. *Environ. Sci. Technol.* **38**, 1338–1347 (2004).

41. B. Yuan, M. Shao, J. De Gouw, D. D. Parrish, S. Lu, M. Wang, L. Zeng, Q. Zhang, Y. Song, J. Zhang, M. Hu, Volatile organic compounds (VOCs) in urban air: How chemistry affects the interpretation of positive matrix factorization (PMF) analysis. *J. Geophys. Res. Atmos.* **117**, D24302 (2012).

42. B. Langford, B. Davison, E. Nemitz, C. N. Hewitt, Mixing ratios and eddy covariance flux measurements of volatile organic compounds from an urban canopy (Manchester, UK). *Atmos. Chem. Phys.* **9**, 1971–1987 (2009).

43. W. J. F. Acton, Z. Huang, B. Davison, W. S. Drysdale, P. Fu, M. Hollaway, B. Langford, J. Lee, Y. Liu, S. Metzger, N. Mullinger, E. Nemitz, C. E. Reeves, F. A. Squires, A. R. Vaughan, X. Wang, Z. Wang, O. Wild, Q. Zhang, Y. Zhang, C. Nicholas Hewitt, Surface-atmosphere fluxes of volatile organic compounds in Beijing. *Atmos. Chem. Phys.* **20**, 15101–15125 (2020).

44. E. Y. Pfannerstill, C. Arata, Q. Zhu, B. C. Schulze, R. Woods, J. H. Seinfeld, A. Bucholtz, R. C. Cohen, A. H. Goldstein, Volatile organic compound fluxes in the agricultural San Joaquin Valley—Spatial distribution, source attribution, and inventory comparison. *Atmos. Chem. Phys.* **23**, 12753–12780 (2023).

45. A. Peron, M. Graus, M. Striednig, C. Lamprecht, G. Wohlfahrt, T. Karl, Deciphering anthropogenic and biogenic contributions to selected non-methane volatile organic compound emissions in an urban area. *Atmos. Chem. Phys.* **24**, 7063–7083 (2024).

46. F. Barbano, E. Brattich, S. Di Sabatino, Characteristic scales for turbulent exchange processes in a real urban canopy. *Boundary Layer Meteorol.* **178**, 119–142 (2021).

47. R. Atkinson, D. L. Baulch, R. A. Cox, J. N. Crowley, R. F. Hampson, R. G. Hynes, M. E. Jenkin, M. J. Rossi, J. Troe, IUPAC Subcommittee, Evaluated kinetic and photochemical data for atmospheric chemistry: Volume II-gas phase reactions of organic species. *Atmos. Chem. Phys.* **6**, 3625–4055 (2006).

48. R. Atkinson, S. M. Aschmann, J. Arey, Rate constants for the gas-phase reactions of OH and NO_3 radicals and O_3 with sabinene and camphene at 296±2 K. *Atmos. Environ. Part A Gen. Top.* **24**, 2647–2654 (1990).

49. R. Atkinson, J. Arey, Gas-phase tropospheric chemistry of biogenic volatile organic compounds: A review. *Atmos. Environ.* **37**, 197–219 (2003).

50. N. M. Donahue, W. Chuang, S. A. Epstein, J. H. Kroll, D. R. Worsnop, A. L. Robinson, P. J. Adams, S. N. Pandis, N. M. Donahue, W. Chuang, S. A. Epstein, J. H. Kroll, D. R. Worsnop, A. L. Robinson, P. J. Adams, S. N. Pandis, Why do organic aerosols exist? Understanding aerosol lifetimes using the two-dimensional volatility basis set. *Environ. Chem.* **10**, 151–157 (2013).

51. J. A. de Gouw, J. B. Gilman, S. W. Kim, B. M. Lerner, G. Isaacman-VanWertz, B. C. McDonald, C. Warneke, W. C. Kuster, B. L. Lefer, S. M. Griffith, S. Dusanter, P. S. Stevens, J. Stutz, Chemistry of volatile organic compounds in the Los Angeles basin: Nighttime

removal of alkenes and determination of emission ratios. *J. Geophys. Res. Atmos.* **122**, 11,843–11,861 (2017).

52. A. Borbon, P. Dominutti, A. Panopoulou, V. Gros, S. Sauvage, M. Farhat, C. Afif, N. Elguindi, A. Fornaro, C. Granier, J. R. Hopkins, E. Liakakou, T. Nogueira, T. Corrêa dos Santos, T. Salameh, A. Armangaud, D. Piga, O. Perrussel, Ubiquity of anthropogenic terpenoids in cities worldwide: Emission ratios, emission quantification and implications for urban atmospheric chemistry. *J. Geophys. Res. Atmos.* **128**, e2022JD037566 (2023).

53. L. Miller, X. Xu, A. Wheeler, D. O. Atari, A. Grgicak-Mannion, I. Luginaah, Spatial variability and application of ratios between BTEX in two Canadian cities. *ScientificWorldJournal* **11**, 2536–2549 (2011).

54. L. Cui, D. Wu, S. Wang, Q. Xu, R. Hu, J. Hao, Measurement report: Ambient volatile organic compound (VOC) pollution in urban Beijing: Characteristics, sources, and implications for pollution control. *Atmos. Chem. Phys.* **22**, 11931–11944 (2022).

55. C. Arata, P. K. Misztal, Y. Tian, D. M. Lunderberg, K. Kristensen, A. Novoselac, M. E. Vance, D. K. Farmer, W. W. Nazaroff, A. H. Goldstein, Volatile organic compound emissions during HOMEChem. *Indoor Air* **31**, 2099–2117 (2021).

56. Y. Liu, P. K. Misztal, C. Arata, C. J. Weschler, W. W. Nazaroff, A. H. Goldstein, Observing ozone chemistry in an occupied residence. *Proc. Natl. Acad. Sci. U.S.A.* **118**, e2018140118 (2021).

57. T. J. Carter, D. G. Poppendieck, D. Shaw, N. Carslaw, A modelling study of indoor air chemistry: The surface interactions of ozone and hydrogen peroxide. *Atmos. Environ.* **297**, 119598 (2023).

58. R. B. Stull, *An Introduction to Boundary Layer Meteorology* (Springer Science & Business Media, 1988).

59. D. Helmig, R. W. Daly, J. Milford, A. Guenther, Seasonal trends of biogenic terpene emissions. *Chemosphere* **93**, 35–46 (2013).

60. J. Zheng, Y. Yu, Z. Mo, Z. Zhang, X. Wang, S. Yin, K. Peng, Y. Yang, X. Feng, H. Cai, Industrial sector-based volatile organic compound (VOC) source profiles measured in manufacturing facilities in the Pearl River Delta, China. *Sci. Total Environ.* **456-457**, 127–136 (2013).

61. B. Yuan, M. Shao, S. Lu, B. Wang, Source profiles of volatile organic compounds associated with solvent use in Beijing, China. *Atmos. Environ.* **44**, 1919–1926 (2010).

62. H. Wang, Y. Qiao, C. Chen, J. Lu, H. Dai, L. Qiao, S. Lou, C. Huang, L. Li, S. Jing, J. Wu, Source profiles and chemical reactivity of volatile organic compounds from solvent use in Shanghai, China. *Aerosol Air Qual. Res.* **14**, 301–310 (2014).

63. F. Klein, S. M. Platt, N. J. Farren, A. Detournay, E. A. Bruns, C. Bozzetti, K. R. Daellenbach, D. Kilic, N. K. Kumar, S. M. Pieber, J. G. Slowik, B. Temime-Roussel, N. Marchand, J. F. Hamilton, U. Baltensperger, A. S. H. Prévôt, I. El Haddad, Characterization of gas-phase organics using proton transfer reaction time-of-flight mass spectrometry: Cooking emissions. *Environ. Sci. Technol.* **50**, 1243–1250 (2016).

64. E. Reyes-Villegas, T. Bannan, M. Le Breton, A. Mehra, M. Priestley, C. Percival, H. Coe, J. D. Allan, Online chemical characterization of food-cooking organic aerosols: Implications for source apportionment. *Environ. Sci. Technol.* **52**, 5308–5318 (2018).

65. J. C. Ditto, J. P. D. Abbatt, A. W. H. Chan, Gas- and particle-phase amide emissions from cooking: Mechanisms and air quality impacts. *Environ. Sci. Technol.* **56**, 7741–7750 (2022).

66. C. E. Stockwell, M. M. Coggon, R. H. Schwantes, C. Harkins, B. Verreyken, C. Lyu, Q. Zhu, L. Xu, J. B. Gilman, A. Lamplugh, J. Peischl, M. A. Robinson, P. R. Veres, M. Li, A. W. Rollins, K. Zuraski, S. Baidar, S. Liu, T. Kuwayama, S. S. Brown, B. C. McDonald, C. Warneke, Urban ozone formation and sensitivities to volatile chemical products, cooking emissions, and NO_x upwind of and within two Los Angeles Basin cities. *Atmos. Chem. Phys.* **25**, 1121–1143 (2025).

67. E. B. Franklin, R. K. Rossell, M. P. Vermeuel, A. De Groodt, K. Rediger, L. D. Yee, J. Marcantonio, T. Maddaleno, C. Osburn, R. E. O'Brien, R. Commane, J. E. Mak, A. H.

Goldstein, D. B. Millet, D. K. Farmer, Emerging drivers of urban aerosol increase global change vulnerability in a US megacity. *NPJ Clim. Atmos. Sci.* **8**, 333 (2025).

68. C. Lyu, C. Harkins, M. Li, K. Mueller, J. Prothero, B. Verreyken, J. B. Miller, S. J. Lehman, J. Peischl, J. B. Gilman, C. Warneke, M. M. Coggon, C. E. Stockwell, S. S. Brown, K. Zuraski, A. Lamplugh, K. R. Gurney, L. Gawuc, P. Dass, R. M. Hoesly, S. J. Smith, T. Oda, L. R. Hutyra, C. K. Gately, C. Granier, J. R. Whetstone, B. C. McDonald, Development of the United States GReenhouse Gas and Air Pollutants Emissions System (GRA^2PES). *J. Geophys. Res. Atmos.* **130**, e2025JD043597 (2025).

69. A. Kumar, C. O'Leary, R. Winkless, W. Dighriri, M. Shaw, D. Shaw, N. Carslaw, T. Dillon, Chemical fingerprints of cooking emissions and their impact on indoor air quality. *Environ. Sci. Process Impacts* **27**, 3665–3682 (2025).

70. Z. Zhang, H. Man, J. Zhao, Y. Jiang, M. Zeng, Z. Cai, C. Huang, W. Huang, H. Zhao, S. Jing, X. Shi, K. He, H. Liu, Primary organic gas emissions in vehicle cold start events: Rates, compositions and temperature effects. *J. Hazard. Mater.* **435**, 128979 (2022).

71. X. He, B. Yuan, Y. Huangfu, S. Wang, Y. Chen, X. Zhang, Y. Sun, T. Karl, M. Shao, Comprehensive characterization and source analysis of VOC fluxes in a Chinese megacity. *Environ. Sci. Technol.* **60**, 6477–6488 (2026).

72. H. Weng, J. Lin, R. Martin, D. B. Millet, L. Jaeglé, D. Ridley, C. Keller, C. Li, M. Du, J. Meng, Global high-resolution emissions of soil NO_x, sea salt aerosols, and biogenic volatile organic compounds. *Sci. Data* **7**, 148 (2020).

73. H. G. Semerjian, T. M. Wong, J. R. Whetstone, NIST Greenhouse Gas Measurements Program: A Decade of Critical Accomplishments (2024).

74. D. Wei, A. Reinmann, L. D. Schiferl, R. Commane, High resolution modeling of vegetation reveals large summertime biogenic CO_2 fluxes in New York City. *Environ. Res. Lett.* **17**, 124031 (2022).

75. U.S. Geological Survey (USGS), Annual NLCD Collection 1 Science Products: U.S. Geological Survey data release (2024); https://doi.org/https://doi.org/10.5066/P94UXNTS.

76. M. Mauder, M. Cuntz, C. Drüe, A. Graf, C. Rebmann, H. P. Schmid, M. Schmidt, R. Steinbrecher, A strategy for quality and uncertainty assessment of long-term eddy-covariance measurements. *Agric. For. Meteorol.* **169**, 122–135 (2013).

77. J. J. Finnigan, A re-evaluation of long-term flux measurement techniques part II: Coordinate systems. *Boundary Layer Meteorol.* **113**, 1–41 (2004).

78. B. Langford, W. Acton, C. Ammann, A. Valach, E. Nemitz, Eddy-covariance data with low signal-to-noise ratio: Time-lag determination, uncertainties and limit of detection. *Atmos. Meas. Tech.* **8**, 4197–4213 (2015).

79. T. W. Horst, A simple formula for attenuation of eddy fluxes measured with first-order-response scalar sensors. *Boundary Layer Meteorol.* **82**, 219–233 (1997).

80. T. Foken, B. Wichura, Tools for quality assessment of surface-based flux measurements. *Agric. For. Meteorol.* **78**, 83–105 (1996).

81. N. Kljun, P. Calanca, M. W. Rotach, H. P. Schmid, A simple two-dimensional parameterisation for Flux Footprint Prediction (FFP). *Geosci. Model Dev.* **8**, 3695–3713 (2015).

82. R. Lucchesi, "File specification for GEOS-5 FP (Forward processing)" (2013).

83. C. Lyu, C. Harkins, M. Li, B. McDonald, J. Prothero, K. Mueller, The U.S. Greenhouse Gas and Air Pollutant Emissions System (GRA^{2}PES) (2024); https://doi.org/10.18434/mds2-3520.

84. U.S. Environmental Protection Agency, 2017 National Emissions Inventory (NEI) Data [Dataset] (2021); https://epa.gov/air-emissions-inventories/2017-national-emissions-inventory-nei-data.

85. A. M. Gorchov Negron, B. C. McDonald, S. A. McKeen, J. Peischl, R. Ahmadov, J. A. De Gouw, G. J. Frost, M. G. Hastings, I. B. Pollack, T. B. Ryerson, C. Thompson, C. Warneke, M. Trainer, Development of a fuel-based oil and gas inventory of nitrogen oxides emissions. *Environ. Sci. Technol.* **52**, 10175–10185 (2018).

86. B. C. McDonald, Z. C. McBride, E. W. Martin, R. A. Harley, High-resolution mapping of motor vehicle carbon dioxide emissions. *J. Geophys. Res. Atmos.* **119**, 5283–5298 (2014).

87. U.S. EPA, Air Pollutant Emissions Trends Data (2024).

88. Esri, World Imagery [basemap] (2026); https://arcgis.com/home/item.html?id=10df2279f9684e4a9f6a7f08febac2a9#.

89. M. P. Vermeuel, D. B. Millet, Emissions of volatile organic compounds from the Fluxes of Reactive Organic Gases in New York (FROG-NY) study at Mineola, NY [Dataset] (2026); https://doi.org/10.13020/p0xq-8q46.

90. M. Müller, T. Mikoviny, W. Jud, B. D'Anna, A. Wisthaler, A new software tool for the analysis of high resolution PTR-TOF mass spectra. *Chemom. Intel. Lab. Syst.* **127**, 158–165 (2013).

91. R. Holzinger, PTRwid: A new widget tool for processing PTR-TOF-MS data. *Atmos. Meas. Tech.* **8**, 3903–3922 (2015).

92. X. Lee, W. J. Massman, B. Law, *Handbook of Micrometeorology* (Springer Dordrecht, 2005), vol. 29, 250 pp.
